# PRMT1‐Mediated LDHA Methylation Drives STAT3 Lactylation to Orchestrate Intestinal Inflammation and Tumorigenesis

**DOI:** 10.1002/advs.202516577

**Published:** 2026-06-01

**Authors:** Hui Wang, Mengyu Zhang, Weipeng Gong, Jiaxuan Wu, Junping Zhang, Wenxiu Zhang, Yue Liu, Kui Wang, Canhua Huang, Jun Zhou, Sijin Wu, Yan Li, Tianliang Li

**Affiliations:** ^1^ School of Basic Medical Sciences Shandong Second Medical University Weifang Shandong China; ^2^ Center for Cell Structure and Function, College of Life Sciences Shandong Normal University Jinan China; ^3^ Department of Gastrointestinal Surgery, Shandong Cancer Hospital and Institute Shandong First Medical University and Shandong Academy of Medical Sciences Jinan Shandong China; ^4^ Wisdom Lake Academic of Pharmacy Xi'an‐Jiaotong Liverpool University Suzhou China; ^5^ Department of Pathology Liangshan County People's Hospital Jining China; ^6^ West China School of Basic Medical Sciences & Forensic Medicine, and State Key Laboratory of Biotherapy and Cancer Center West China Hospital, Sichuan University, and Collaborative Innovation Center for Biotherapy Chengdu China; ^7^ State Key Laboratory of Medicinal Chemical Biology College of Life Sciences, Nankai University Tianjin China

**Keywords:** interleukin‐10, intestinal inflammation, lactylation, PRMT1, STAT3

## Abstract

Signal transducer and activator of transcription 3 (STAT3) activation is crucial in intestinal inflammation and tumorigenesis. However, its metabolic regulation is not well understood. Herein, we identified a macrophage‐dependent methionine‐S‐adenosylmethionine (SAM)‐protein arginine methyltransferase 1 (PRMT1)‐lactate dehydrogenase A (LDHA)‐lactate axis that controls intestinal inflammation through STAT3 regulation. Specifically, SAM promoted STAT3 Y705 phosphorylation and upregulated anti‐inflammatory interleukin‐10 expression in macrophages. Additionally, genetic ablation of PRMT1 in myeloid cells not only impairs STAT3 activation but also exacerbates colitis and promotes inflammation‐associated tumorigenesis. Mechanistically, PRMT1 directly methylates LDHA at R268/R269, thereby enhancing its activity and lactate production. Subsequently, the resulting lactate induces STAT3 lactylation at K709, stabilizing an open conformation that facilitates Y705 phosphorylation. Importantly, disruption of this modification through K709‐specific inhibition effectively blocks STAT3 activation and, consequently, exacerbates colitis progression. Overall, this study reveals STAT3 lactylation as a novel post‐translational modification that integrates methionine metabolism with glycolytic flux to regulate intestinal inflammation, highlighting the critical role of immunometabolism in colonic inflammation.

## Graphical abstract

This study identifies an immunometabolic axis wherein SAM‐driven PRMT1 methylates LDHA, enhancing its activity. The resultant lactate induces STAT3 K709 lactylation, which stabilizes an active conformation to promote STAT3 phosphorylation and IL‐10 expression. Disruption of this circuit exacerbates colitis and associated tumorigenesis, revealing a potential therapeutic target for IBD.

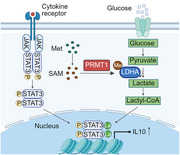

## 1 Introduction

Inflammatory bowel disease (IBD), comprising ulcerative colitis (UC) and Crohn's disease (CD) [1], is a chronic inflammatory condition associated with an increased risk of colorectal cancer [2, 3]. Signal transducer and activator of transcription 3 (STAT3) plays a central role in IBD pathogenesis, with genetic variations in the STAT3 gene linked to heightened susceptibility [4]. Myeloid‐derived STAT3 exhibits strong anti‐inflammatory properties in experimental colitis, highlighting its therapeutic potential [5]. Additionally, STAT3 is essential for the survival and proliferation of intestinal epithelial cells, which are critical for maintaining intestinal homeostasis [6]. Understanding the interaction between STAT3 and IBD is vital for developing targeted therapies to manage IBD.

Immunometabolism connects metabolic reprogramming to hyperinflammation. Immune cell activation triggers metabolic shifts, including increased glucose uptake and glycolysis [7]. Anaerobic glycolysis produces lactate, which exerts immunomodulatory effects on macrophage function and innate immunity [8, 9, 10, 11]. Notably, lactate can induce protein lactylation, a post‐translational modification that alters protein structure and function, thereby influencing immune responses and inflammation [9, 10, 11]. While protein lactylation is emerging as a potential therapeutic target, its precise role in regulating STAT3 activity remains poorly understood.

Protein arginine methyltransferases (PRMTs) catalyze the methylation of arginine residues, influencing key cellular processes such as gene expression and signal transduction [12]. Among these enzymes, PRMT1 is critical for macrophage function and innate immune responses [13, 14, 15, 16]. However, its role in STAT3 activation has not been fully elucidated.

In this study, we identified a close relationship to S‐adenosylmethionine (SAM) in driving STAT3 activation following lipopolysaccharide (LPS) stimulation. Both PRMT1 and lactate dehydrogenase A (LDHA) were shown to promote STAT3 activation, leading to increased interleukin‐10 (IL‐10) production in macrophages and reduced dextran sulfate sodium (DSS)‐induced colitis. Mechanistically, PRMT1 interacts with LDHA, catalyzing asymmetric dimethylation at arginine residues 268 and 269, which enhances LDHA activity and lactate production. Elevated lactate levels induce STAT3 lactylation at lysine 709, facilitating Tyr705 phosphorylation and STAT3 activation. Inhibition of STAT3 lactylation using a trans‐activator of transcription (TAT)‐tagged non‐lactylated peptide blocked STAT3 activation and IL‐10 production, exacerbating DSS‐induced colitis. These findings underscore the regulatory role of the SAM–PRMT1–LDHA–lactate axis in STAT3 activation during intestinal inflammation.

## 2 Results

### 2.1 S‐Adenosylmethionine is Associated With STAT3 Activation

**FIGURE 1:**
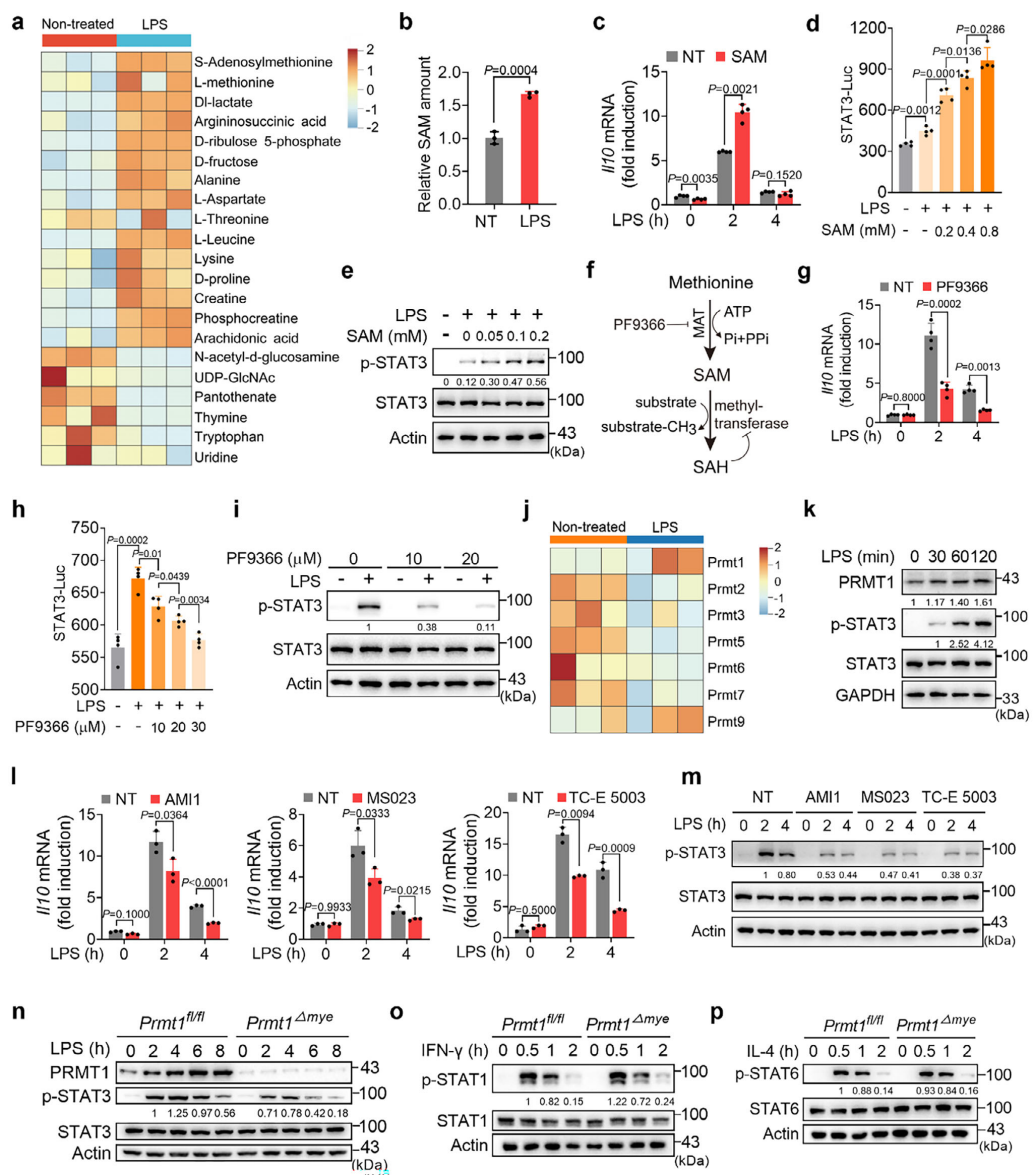
SAM and PRMT1 promotes STAT3 activation. (a) Heatmap of metabolites in BMDMs stimulated with or without LPS (100 ng/mL) for 4 h. (b) SAM levels in BMDMs treated with or without LPS (100 ng/mL) for 4 h (*n* = 3). The data are derived from the metabolome. (c) Transcripts of *Il10* in BMDMs pretreated with SAM (200 µM) for 16 h, followed by LPS (100 ng/mL) stimulation for 2 or 4 h, measured by RT‐qPCR (*n* = 4). (d) STAT3 luciferase activity in THP‐1 cells transfected with STAT3 luciferase reporter plasmids, pretreated with SAM for 12 h, followed by LPS (100 ng/mL) stimulation for 10 h (*n* = 4). (e) Immunoblot analysis of phosphorylated STAT3 (Y705) in BMDMs pretreated with different concentrations of SAM for 12 h, followed by LPS stimulation (100 ng/mL) for 2 h. (f) Schematic of SAM generation from methionine. (g) *Il10* transcripts in BMDMs pretreated with PF9366 (10 µM) for 12 h, followed by with LPS (100 ng/mL) stimulation for 2 or 4 h (*n* = 4). (h) STAT3 luciferase activity in THP‐1 cells transfected with STAT3 luciferase reporter plasmids, treated with PF9366 for 12 h, followed by LPS stimulation for 10 h (*n* = 4). (i) Immunoblotting of phosphorylated STAT3 (Y705) in BMDMs pretreated with PF9366 for 12 h, followed by LPS (100 ng/mL) stimulation for 2 h or left untreated. (j) Heatmap of gene expression in BMDMs treated with LPS (100 ng/mL) for 2 h. (k) Immunoblotting of PRMT1, phosphorylated STAT3 (Y705) and total STAT3 in BMDMs stimulated with LPS (100 ng/mL) for the indicated times. (l, m) *Il10* transcripts (l) and immunoblotting of phosphorylated STAT3 (Y705) and total STAT3 (m) in BMDMs pretreated with AMI1 (10 µM), MS023 (50 µM), or TC‐E 5003 (2 µM) for 16 h, followed by LPS (100 ng/mL) stimulation for the indicated time points (*n* = 3). (n–p), Immunoblotting of PRMT1, phosphorylated STAT3 (Y705) and total STAT3 (n), phosphorylated STAT1 (Tyr701) and total STAT1 (o), and phosphorylated STAT6 (Tyr641) and total STAT6 (p) in *Prmt1^fl/fl^* and *Prmt1^Δmye^* BMDMs stimulated with LPS (100 ng/mL) (n), IFN‐γ (20 ng/mL) (o), or IL‐4 (20 ng/mL) (p) for the indicated times. The error bars represent the SD. Statistical significance was determined using unpaired *t*‐test (b, c, d, g, h, l). The data presented in (e, i, k, m, n–p) are representative of three independent experiments. *p* < 0.05 is considered statistically significant. Original blot can be found in Figure S9.

The intestine is a complex ecosystem where Toll‐like receptors (TLRs) detect microbial signals to initiate immune responses, triggering inflammatory pathways and modulating intestinal immunity through cytokine and chemokine production [17, 18]. To explore metabolic responses to microbial challenges, we conducted unbiased metabolomics profiling in mouse bone marrow‐derived macrophages (BMDMs) stimulated with LPS for 4 h (Data S1). Heat map analysis revealed significant upregulation of amino acid and glucose metabolites (Figure 1a), including S‐adenosylmethionine (SAM), a key intermediate in methionine metabolism (Figure 1b). Consistent with prior evidence that extracellular SAM enters macrophages and elevates intracellular levels [19], SAM treatment substantially elevated intracellular SAM concentrations in BMDMs relative to vehicle controls (Figure S1a). We investigated SAM's role in STAT3 activation by examining its effects on the transcription of STAT3 target genes *Il10*, which were significantly upregulated in LPS‐stimulated BMDMs (Figure 1c). A STAT3 luciferase reporter assay in THP‐1 cells confirmed that SAM induces STAT3 activation and potentiates LPS‐induced STAT3 activation in a dose‐dependent manner (Figure 1d). Additionally, SAM enhanced STAT3 phosphorylation at Y705, a hallmark of STAT3 activation, in response to LPS (Figure 1e). SAM, synthesized from methionine by methionine adenosyltransferase (MAT), serves as a universal co‐substrate and essential methyl donor for transmethylation reactions involved in one‐carbon metabolism and epigenetic regulation (Figure 1f) [19, 20, 21]. Inhibition of MAT using the allosteric inhibitor PF9366 reduced LPS‐induced *Il10* transcription (Figure 1g), decreased LPS‐induced STAT3 luciferase activity in a dose‐dependent manner (Figure 1h), and significantly attenuated STAT3 phosphorylation (Figure 1i). These findings establish SAM as a critical regulator of STAT3 activation.

SAM's role in methyl transfer reactions includes facilitating protein methylation and post‐translational modifications essential for cellular function [19, 21]. Among methyltransferases, PRMTs are key regulators of immunological processes. PRMTs are categorized into three types: type I enzymes (PRMT1, PRMT2, PRMT3, CARM1/PRMT4, PRMT6, and PRMT8) catalyze asymmetric dimethylation; type II enzymes (PRMT5 and PRMT9) perform symmetric dimethylation; and PRMT7, the sole type III enzyme, mediates monomethylation of arginines [22]. To investigate PRMT involvement in STAT3 activation, we exposed BMDMs to LPS and conducted RNA sequencing (Figure S1b, Data S2). Among PRMT family members, PRMT1 showed the most significant upregulation (Figure 1j and Figure S1c). Validation experiments confirmed increased PRMT1 expression and elevated STAT3 phosphorylation in LPS‐stimulated BMDMs (Figure 1k), implicating PRMT1 in STAT3 activation.

To confirm PRMT1's role in STAT3 activation, BMDMs pre‐treated with PRMT inhibitors (AMI1, MS023, and TC‐E 5003, targeting type I‐III, type I, and PRMT1 specifically, respectively) before LPS stimulation exhibited decreased *Il10* transcription (Figure 1l) and reduced STAT3 phosphorylation (Figure 1m), with effects resembling those of TC‐E 5003 alone. These results highlight PRMT1's pivotal role in STAT3 activation. Collectively, our findings establish a strong link between SAM, PRMT1, and STAT3 activation.

### 2.2 PRMT1 Mediates STAT3 Activation

To further explore PRMT1's role in the innate immune system, we generated myeloid‐specific *Prmt1* knockout mice (*Prmt1^Δmye^*) by crossing *Prmt1^fl/fl^* mice with *Lyz2*‐Cre mice [15]. *Prmt*1*^fl/fl^* mice served as wild‐type (WT) controls. STAT3 phosphorylation was significantly reduced in *Prmt1^Δmye^ BMDMs* compared to that in *Prmt1^fl/fl^ BMDMs* in response to both LPS (Figure 1n) and IL‐6 (Figure S2a). However, STAT1 phosphorylation in response to IFN‐γ (Figure 1o) and STAT6 phosphorylation following IL‐4 stimulation (Figure 1p) remained unaffected, suggesting a specific role of PRMT1 in modulating STAT3 signaling in macrophages.

In *Prmt1^Δmye^* macrophages, *Il10* transcripts were decreased, while *Il6*, *Tnfa*, and *Il12a*, transcripts were elevated (Figure S2b). This was accompanied by altered protein levels of IL‐10, IL‐6, and tumor necrosis factor (TNF)‐α after LPS stimulation (Figure S2c). Similar hyper‐inflammatory phenotypes were observed with TLR2 (Pam3CSK4) and TLR9 (CpG‐ODN) agonists (Figure S2d,e).

Consistent with elevated pro‐inflammatory cytokine production, in BMDMs from *Prmt1^Δmye^*, LPS stimulation induced faster IκBα degradation, stronger phosphorylation of IκBα (Ser32/36) and p65 (Ser536), and greater p65 nuclear translocation than in *Prmt1^fl/fl^* controls (Figure S2f,g). Additionally, PRMT1 knockdown in HEK293T cells significantly increased NF‐κB‐driven luciferase reporter activity, which was dose‐dependently suppressed by re‐expression of wild‐type PRMT1 (Figure S2h,i). Furthermore, pretreatment of BMDMs with the NF‐κB inhibitor BAY 11–7082 prior to LPS stimulation largely abolished the increased TNF‐α and IL‐6 mRNA and protein production in PRMT1‐deficient cells, normalizing levels to those of inhibitor‐treated *Prmt1^fl/fl^* controls (Figure S2j,k). These findings collectively indicate that PRMT1 normally restrains NF‐κB pathway activation in myeloid cells, thereby limiting excessive inflammatory responses upon innate immune stimulation.

### 2.3 PRMT1‐Deficient Mice are More Susceptible to DSS‐Induced Colitis

**FIGURE 2:**
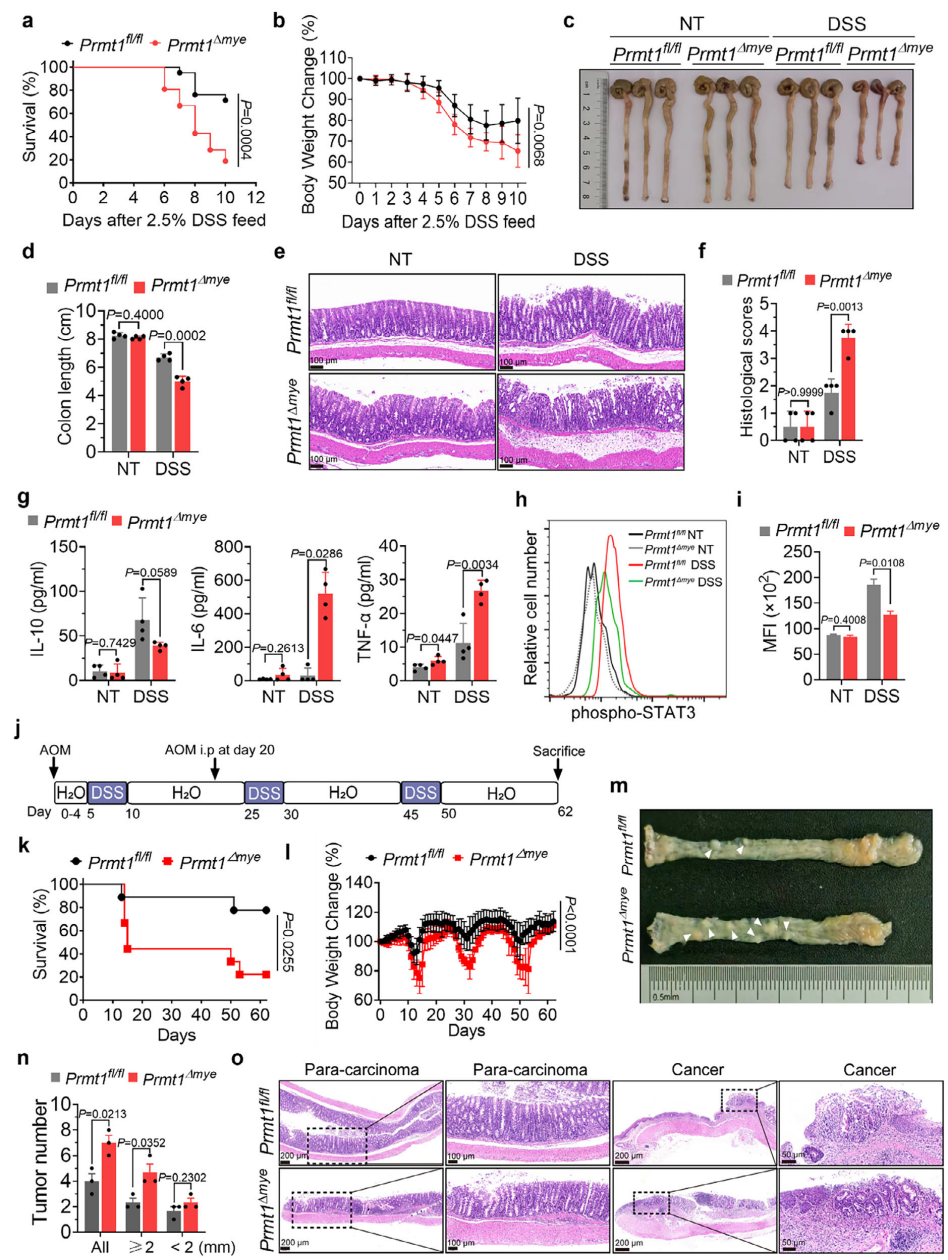
PRMT1‐deficient mice are more susceptible to experimental colitis and CAC. (a–i) *Prmt1^fl/fl^* and *Prmt1^Δmye^* mice received 2.5% DSS in drinking water for 5 days, followed by regular water for 5 days. (a, b) Kaplan–Meier plot of animal survival (a) and body weight loss (b) in *Prmt1^fl/fl^* and *Prmt1^Δmye^* mice (*n* = 21). (c, d) Representative colon images (c) and quantification of colon length (d) in DSS‐treated *Prmt1^fl/fl^* and *Prmt1^Δmye^* mice. (e) H&E staining showing crypt loss and immune cell infiltration in colon tissue sections (Scale bar, 100 µm). (f) Histological analysis of colon tissues with scores determined in a double‐blind manner (*n* = 4). (g) IL‐10, IL‐6 and TNF‐α levels in colon explant cultures, measured by ELISA (*n* = 4). (h, i) STAT3 phosphorylation in colon macrophages (CD11b^+^F4/80^+^), measured by FACS, with histogram (h) and quantification (i). NT, not treated (*n* = 4). (j) Schematic of the AOM/DSS model of CAC. (k, l) Kaplan–Meier survival plot (k) and body weight loss (l) in *Prmt1^fl/fl^* and *Prmt1^Δmye^* mice after CAC induction (*n* = 9). (m) Macroscopic polyps (arrows) were identified in the distal and mid‐colons isolated from *Prmt1^fl/fl^* and *Prmt1^Δmye^* mice that had completed the entire course of the CAC model (*n* = 9). (n) The number of macroscopic polyps was quantified. (o) H&E staining of colon tissues showing histopathology such as crypt loss, immune cell infiltration, and polyp formation in *Prmt1^fl/fl^* and *Prmt1^Δmye^* mice that had completed the entire course of the CAC model (*n* = 9). Error bars represent the SD. Statistical significance were determined using Mantel‐Cox test (a, k) or unpaired *t*‐test (b, d, f, g, i, l, n). *p* < 0.05 is considered statistically significant.

Several studies highlight the important roles of STAT3 and IL‐10 in regulating intestinal inflammation and tumorigenesis [23, 24, 25]. For instance, myeloid‐specific deletion of *Stat3* worsens chemically induced colitis [5], while IL‐10‐deficient mice are more prone to spontaneous colitis [26]. To evaluate the role of myeloid‐derived PRMT1 in intestinal inflammation, *Prmt1^fl/fl^* and *Prmt1^Δmye^* mice were subjected to 2.5% DSS for 5 days, with survival monitored for 10 days. *Prmt1^Δmye^* mice showed significantly higher mortality (Figure 2a), severe weight loss (Figure 2b), and pronounced colon shortening (Figure 2c,d) compared to *Prmt1^fl/fl^* mice. Histological analysis revealed increased immune cell infiltration (Figure 2e) and higher histological scores (Figure 2f) in DSS‐treated *Prmt1^Δmye^* mice. Colon explants from *Prmt1^Δmye^* mice produced elevated pro‐inflammatory cytokines IL‐6, TNF‐α, while the anti‐inflammatory cytokine IL‐10 was significantly reduced (Figure 2g). To establish the physiological relevance of the PRMT1‐STAT3‐IL‐10 axis in intestinal inflammation, flow cytometry of isolated colon CD11b+F4/80+ macrophages (Figure S3a, gating strategy) revealed significantly decreased phosphorylated STAT3 levels in *Prmt1^Δmye^* mice compared with *Prmt1^fl/fl^* controls (Figure 2h,i), with a consistent reduction also observed in colon tissue immunoblots (Figure S3b), together confirming impaired STAT3 activation in the intestinal myeloid compartment during colitis. These findings suggest that PRMT1 deficiency exacerbates DSS‐induced colitis.

The impact of myeloid‐derived PRMT1 on colorectal cancer (CAC) was assessed using an AOM/DSS model (Figure 2j). *Prmt1^Δmye^* mice exhibited reduced survival (Figure 2k), exacerbated weight loss (Figure 2l), and increased tumor burden (Figure 2m, n). Histological analysis revealed larger and more numerous dysplastic lesions with heightened immune cell infiltration (Figure 2o). Colon macrophages from *Prmt1^Δmye^* mice exhibited diminished STAT3 phosphorylation (Figure S3c), and colon explants showed elevated pro‐inflammatory cytokine levels with reduced IL‐10 (Figure S3d). These results indicate that myeloid‐specific PRMT1 deletion disrupts STAT3–IL‐10 signaling, exacerbating colonic inflammation and tumorigenesis.

To validate the translational relevance of our findings in human inflammatory bowel disease (IBD), we performed tyramide signal amplification (TSA)‐based multiplex immunofluorescence staining on formalin‐fixed paraffin‐embedded (FFPE) colon mucosa samples from clinically annotated IBD patients with active disease or remission and non‐IBD controls. A four‐plex panel detecting CD68, PRMT1, LDHA, and phospho‐STAT3 (Y705) revealed cytoplasmic co‐localization of PRMT1 and LDHA in CD68^+^ macrophages within active IBD lesions, whereas phospho‐STAT3 (Y705) was predominantly nuclear with minimal overlap with cytoplasmic PRMT1 (Figure S3e). Active IBD samples showed higher cytoplasmic PRMT1/LDHA co‐localization intensity and elevated nuclear phospho‐STAT3 (Y705) signal intensity in CD68^+^ macrophages compared with remission and control tissues. Cytoplasmic PRMT1/LDHA co‐localization degree and nuclear phospho‐STAT3 (Y705) intensity in CD68^+^ macrophages correlated positively with clinical disease activity indices (Figure S3f). In a complementary four‐plex panel on the same cohort, simultaneous detection of PRMT1, phospho‐LDHA (Y10), phospho‐STAT3 (Y705), and IL‐10 revealed elevated expression of all four markers in active IBD mucosa compared with remission and control samples (Figure S3g). Cytoplasmic staining was observed for PRMT1, phospho‐LDHA (Y10), and IL‐10, with concomitant nuclear phospho‐STAT3 (Y705). Signal intensities of these markers correlated positively with histologic disease activity scores (Figure S3h).

Given the critical role of STAT3 in Th17 differentiation [27, 28], we examined whether myeloid PRMT1 contributes to this process. Naïve CD4^+^ T cells (CD4^+^ CD44^−^ CD62L^+^) were sorted from spleens of *Prmt1^fl/fl^* and *Prmt1^Δmye^* mice (Figure S3i) and polarized under Th17 conditions. Myeloid‐specific *Prmt1* deletion significantly reduced IL‐17A secretion, as measured by ELISA (Figure S3j). These data demonstrate that PRMT1 in myeloid cells provides cell‐extrinsic support for Th17 differentiation.

### 2.4 STAT3 Activation by PRMT1 Is Dependent on Its Enzymatic Activity

PRMT1 is characterized by an N‐terminal methyltransferase domain, a C‐terminal β‐barrel domain, and an α‐helical dimerization arm connecting these domains [29]. Beyond its enzymatic role, PRMT1 also performs enzymatic‐independent functions [30]. To assess whether PRMT1‐mediated STAT3 activation depends on its enzymatic activity, we used genetic and pharmacological approaches. A SAM‐binding‐deficient PRMT1 mutant (E162Q) was generated [16, 31, 32]. Reintroduction of wild‐type PRMT1 into PRMT1‐knockdown THP‐1 cells restored STAT3 activation and IL‐10 production at both mRNA and protein levels after LPS stimulation. However, the E162Q mutant failed to rescue STAT3 activation or IL‐10 production (Figure S4a,b). TC‐E 5003 significantly impairs STAT3 activation and IL‐10 production in bone marrow‐derived macrophages (BMDMs) upon pre‐treatment and subsequent LPS stimulation (Figure S4c,d). Collectively, these findings highlight PRMT1's role in promoting STAT3 activation and mitigating DSS‐induced colitis through its enzymatic activity.

### 2.5 PRMT1 Interacts With LDHA and Regulates Its Activity

**FIGURE 3:**
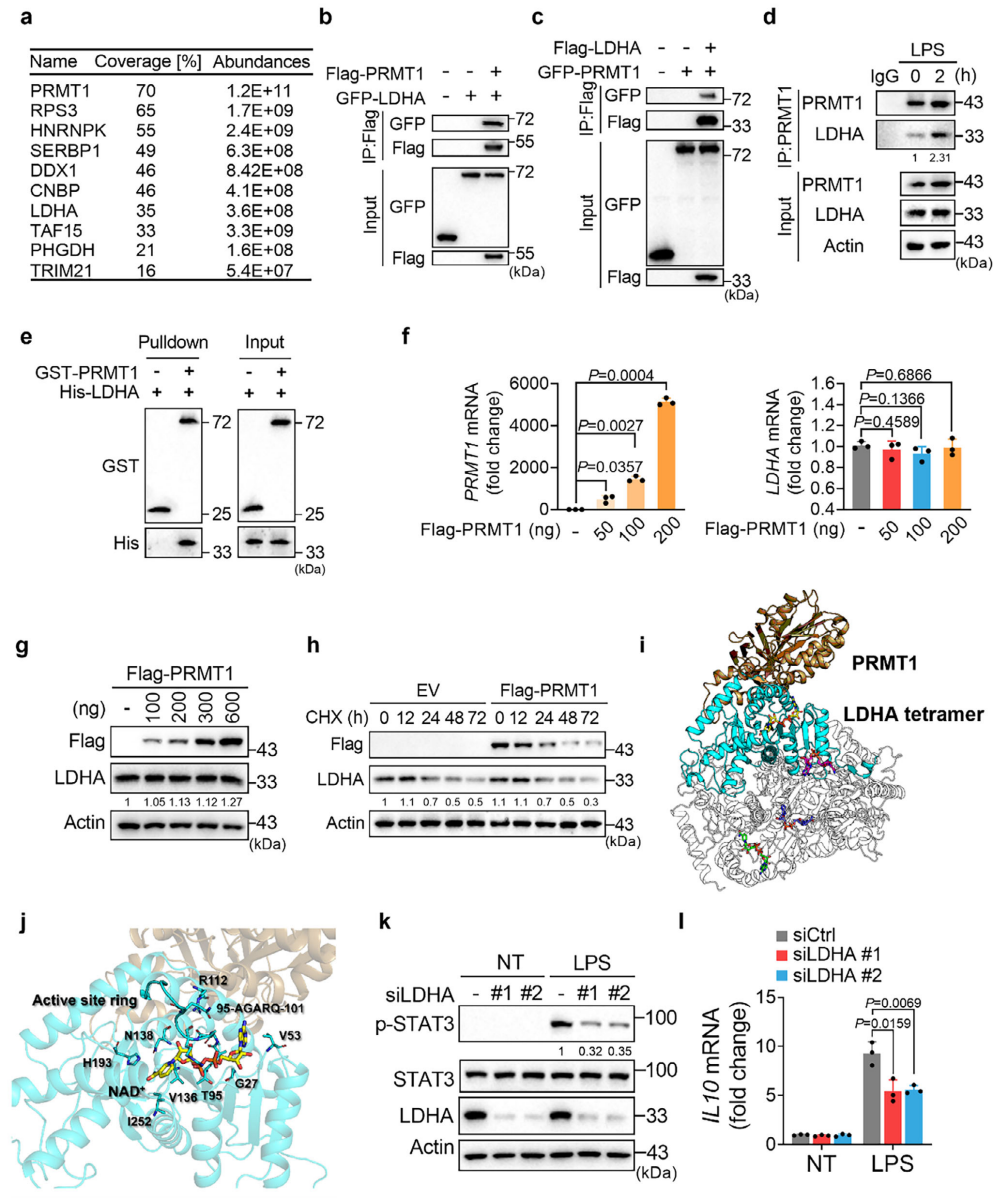
PRMT1 interacts with LDHA and regulates its activity. (a) PRMT1 binding partners with high coverage were identified through mass spectrometry. Coverage refers to the percentage of a protein's amino acid sequence that was detected and matched to peptide sequences during mass spectrometry analysis, while abundance represents the relative quantity of the protein based on the signal intensity of its corresponding peptides. (b,c) 293T cells were transfected for 30 h with the indicated plasmids. Immunoprecipitated proteins were analyzed using the specified antibodies. (d) Total PRMT1 was immunoprecipitated from BMDMs left untreated or stimulated with LPS (100 ng/mL) for 2 h, followed by immunoblotting with anti‐LDHA antibody. (e) Pulldown assay of purified GST‐PRMT1 and His‐LDHA in vitro. (f) The transcripts of *PRMT1* and *LDHA* in HT29 cells transfected with different amounts of PRMT1 expression plasmids (*n* = 3). (g) Immunoblotting of LDHA in HT29 cells transfected with different amounts of PRMT1 expression plasmids. (h) HT29 cells transfected with PRMT1 expression plasmids and then treated with CHX (10 µg/mL) for the indicated times before immunoblotting analysis was performed. (i) Protein‐protein interaction modeling result between PRMT1 (colored wheat) and chain A (colored cyan) within the LDHA tetramer structure. (j) The active site ring structure (cyan cartoon) and key residues (cyan sticks) for NAD^+^ (yellow sticks) binding in PRMT1 (wheat cartoon) interaction system, and the yellow dash lines indicate the hydrogen‐bond networks between key residues and NAD^+^. (k, l) Immunoblotting of phosphorylated STAT3 (Y705), total STAT3, and LDHA (k), transcripts of *IL10* (l) (*n* = 3) in THP‐1 cells transfected with either non‐targeting control siRNA or siRNA against LDHA, followed by LPS stimulation. Error bars represent the SD. Statistical significance was determined using unpaired *t*‐test (f, l). The data presented in (b–e, g, h, k) are representative of three independent experiments. *p* < 0.05 is considered statistically significant. Original blot can be found in Figure S10.

To elucidate PRMT1's role in STAT3 activation, we used mass spectrometry‐based proteomics to identify its interactome. Flag‐tagged PRMT1 was expressed in HEK293T cells, and co‐immunoprecipitation with an anti‐Flag antibody identified interacting proteins (Data S3). LDHA, whose substrate lactate is elevated during LPS stimulation (Figure 1a), emerged as a key binding partner (Figure 3a). LDHA was selected as a candidate interactor for validation based on its central role in lactate production and the observed increase in lactate levels upon LPS stimulation (Figure 1a), which positioned it as a plausible link between PRMT1 activity and downstream STAT3 lactylation. PRMT1–LDHA interaction was validated through reciprocal immunoprecipitation (Figure 3b,c). Notably, endogenous PRMT1–LDHA binding increased upon LPS stimulation (Figure 3d). To further confirm a direct physical interaction between PRMT1 and LDHA, we performed GST pull‐down assays using purified recombinant proteins. As shown in Figure 3e, LDHA was specifically pulled down by GST‐PRMT1, but not by GST alone, confirming a robust and specific direct interaction. Overexpression of PRMT1 did not affect LDHA expression at either the transcript (Figure 3f and Figure S5a) or protein level (Figure 3g and Figure S5b), nor did it alter LDHA protein stability (Figure 3h). Consistently, LDHA protein levels remained comparable between *Prmt1^fl/fl^* and *Prmt1^Δmye^* BMDMs (Figure S5c).

To investigate PRMT1's impact on LDHA activity, we performed molecular docking of PRMT1 with the LDHA tetramer (chain A) and conducted molecular dynamics (MD) simulations (Figure 3i). PRMT1 binding restricted the dynamics of LDHA's active pocket, particularly residues 95–105, which regulate catalytic activity. Residue fluctuations in chain A were significantly reduced compared to those in other chains (Figure S5d), leading to tighter NAD^+^ binding (Figure S5e) and enhanced LDHA activity. In the LDHA‐A chain, residues 95–99 and 136–138 formed a robust interaction network with NAD^+^ (Figure 3j). Energy contribution analysis confirmed these residues as key contributors to NAD^+^ binding in the LDHA‐A chain (Figure S5f).

Next, we assessed LDHA's role in STAT3 activation. LDHA knockdown in THP‐1 cells significantly impaired LPS‐induced STAT3 phosphorylation (Figure 3k) and reduced transcription of *IL10* during LPS stimulation (Figure 3l). These results demonstrate that LDHA is crucial for modulating STAT3 activation.

### 2.6 PRMT1 Activates STAT3 by Methylating LDHA at R268 and R269

**FIGURE 4:**
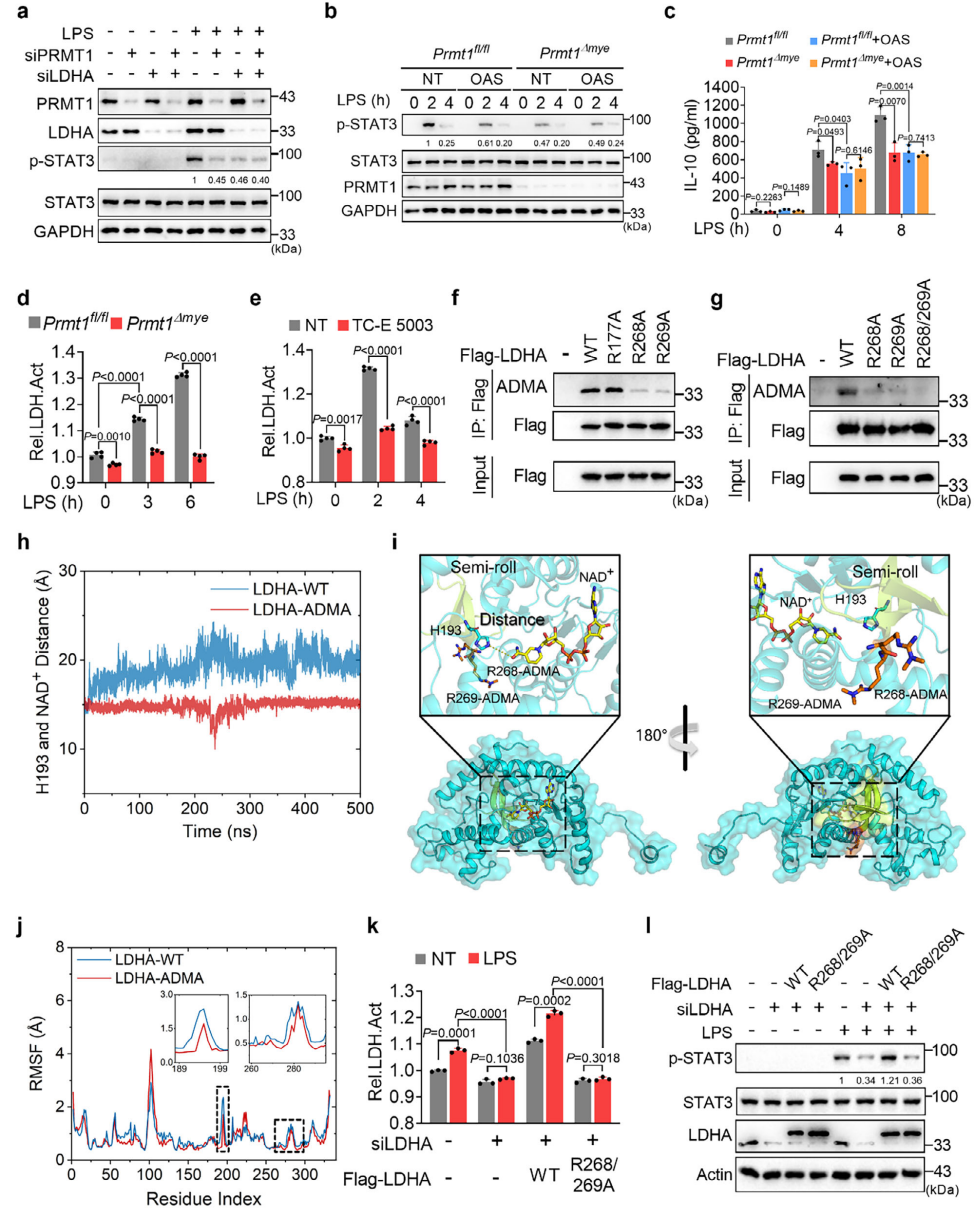
PRMT1 regulates STAT3 activation through LDHA. (a) Immunoblotting of phosphorylated STAT3 (Y705), total STAT3, PRMT1 and LDHA in THP‐1 cells transfected with control siRNA (siCtrl), siRNA targeting LDHA (siLDHA), siRNA targeting PRMT1 (siPRMT1), or a combination of siLDHA and siPRMT1, followed by LPS stimulation. (b,c) Immunoblot analysis of phosphorylated STAT3 (Y705), total STAT3 and PRMT1 (b), and quantification of IL‐10 protein levels in the supernatant (c) from *Prmt1^fl/fl^* and *Prmt1^Δmye^* BMDMs pretreated with OAS (10 mM), followed by LPS stimulation for the indicated times (*n* = 3). (d, e) The intracellular LDH activity in *Prmt1^fl/fl^* and *Prmt1^Δmye^* BMDMs (d) or in BMDMs left untreated or pretreated with TC‐E 5003 (2 µM) for 16 h (e), followed by LPS (100 ng/mL) stimulation for the indicated times (*n* = 4). (f, g), ADMA modification of LDHA or the specified LDHA R‐to‐A mutants in HT29 cells transfected with the relevant expression plasmids, followed by immunoprecipitation and subsequent immunoblotting analysis. (h) Distance between H193 and NAD^+^, with LDHA WT system shown in blue curve and ADMA‐modified LDHA system shown in red curve. (i) Front and back view of ADMA modification structure: the semi‐roll structure is depicted as a yellow cartoon, NAD^+^ is represented in yellow sticks, His193 is shown in cyan sticks, the ADMA‐modified residues R268 and R269 are highlighted in orange, and the yellow dash line indicates the distance between H193. (j) RMSF curves of the LDHA structure under various ADMA‐modified conditions, with the two inset figures highlighting the fluctuations in specific regions: residues 188–200 and 258–288, respectively, offering a detailed view of the fluctuations surrounding the catalytic residue H193 and the α/β complex structure in LDHA. (k,l), Intracellular LDHA activity (k) (*n* = 3) and immunoblotting of phosphorylated STAT3 (Y705), total STAT3, and LDHA (l) in THP‐1 cells transfected with control siRNA (siCtrl), siRNA targeting LDHA (siLDHA), or siLDHA in combination with LDHA WT or LDHA R268/269A mutant expression plasmids, followed by either untreated or LPS stimulation for 2 h. Error bars represent the SD. Statistical significance was determined using unpaired *t*‐test (c–e, k). The data presented in (a, b, l) are representative of three independent experiments. *p* < 0.05 is considered statistically significant. Original blot can be found in Figure S11.

Given the PRMT1‐LDHA interaction, we examined whether PRMT1 mediates STAT3 activation through LDHA. Notably, double knockdown of PRMT1 and LDHA did not further reduce STAT3 luciferase activity (Figure S6a), and reduce the phosphorylation levels of STAT3 (Figure 4a). These results indicate that PRMT1 facilitates STAT3 activation through LDHA.

To examine the role of LDHA enzymatic activity in STAT3 activation, the LDHA inhibitor sodium oxamate (OAS) was utilized. Pretreatment with OAS significantly inhibited lactate dehydrogenase (LDH) activity in both *Prmt1^fl/fl^* and *Prmt1^Δmye^* BMDMs (Figure S6b). In WT BMDMs, OAS treatment resulted in a dose‐dependent reduction in STAT3 phosphorylation (Figure S6c). Conversely, LPS stimulation led to markedly diminished STAT3 activation in *Prmt1^Δmye^* BMDMs, with levels comparable to those in *Prmt1^fl/fl^* BMDMs treated with OAS (Figure 4b). Similarly, OAS treatment significantly reduced IL‐10 protein levels in *Prmt1^fl/fl^* BMDMs to levels comparable to those in *Prmt1^Δmye^* BMDMs, regardless of OAS pretreatment (Figure 4c). Collectively, these findings demonstrate that PRMT1 promotes STAT3 activation via LDHA activity.

To investigate whether PRMT1 interacts with LDHA and regulates its activity, intracellular LDH activity was measured in *Prmt1^fl/fl^* and *Prmt1^Δmye^* BMDMs following LPS stimulation. LPS induced a time‐dependent increase in LDH activity in *Prmt1^fl/fl^* BMDMs, whereas activity remained significantly lower in *Prmt1^Δmye^* BMDMs (Figure 4d). Additionally, treatment with TC‐E 5003 markedly inhibited intracellular LDH activity in LPS‐stimulated WT BMDMs compared to controls (Figure 4e). Further analysis revealed that PRMT1 mediates asymmetric dimethylarginine (ADMA) modification of LDHA (Figure S6d). To identify specific arginine residues undergoing ADMA modification, the RGG‐rich region of the LDHA sequence was analyzed, identifying residues R177, R268, and R269 as potential modification sites. Mutating these residues to alanine (A) in a series of LDHA constructs revealed that mutations at R268 and R269 significantly reduced the ADMA signal without affecting LDHA protein abundance, indicating these residues are modified by ADMA (Figure 4f). Double mutations confirmed that R268 and R269 are the ADMA modification sites on LDHA (Figure 4g). To further validate the functional importance of arginine methylation at these residues while preserving local charge, we generated the R268/269K double mutant. This charge‐preserving mutant exhibited a marked decrease in ADMA signal, comparable to the R268/269A mutant (Figure S6e), confirming that methylation at R268 and R269 is critical for LDHA modification by PRMT1.

To directly test whether PRMT1‐catalyzed methylation increases LDHA enzymatic activity, we established a cell‐free methylation system using purified recombinant proteins. Wild‐type LDHA was incubated with PRMT1 and SAM (methylated group), with SAM alone (no PRMT1), or with PRMT1 alone (no SAM). After removal of PRMT1 and residual SAM by Ni‐NTA purification, LDHA activity was measured by a colorimetric assay. LDHA that had been methylated by PRMT1 exhibited markedly higher catalytic activity compared to LDHA from the two control groups, which did not differ significantly from each other (Figure S6g). This result provides direct biochemical evidence that PRMT1‐mediated methylation enhances LDHA activity, supported by the direct interaction confirmed by GST pull‐down (Figure 3e). To determine whether the decreased activity of the R268K/R269K mutant reflects loss of methylation or simply disruption of critical arginine residues, we compared the intrinsic activities of purified recombinant wild‐type LDHA and the methylation‐resistant R268K/R269K double mutant under identical cell‐free conditions. The mutant exhibited significantly lower basal activity than wild‐type LDHA (Figure S6h). Importantly, both proteins were expressed and purified under identical conditions and showed comparable purity by SDS‐PAGE (Figure S6i), arguing against the possibility that the reduced activity of the mutant is due to global misfolding or degradation. These results indicate that R268 and R269 are important for the intrinsic catalytic function of LDHA, and that loss of methylation at these sites directly impairs its enzymatic activity. To directly test whether the PRMT1‑induced increase in LDHA activity depends on methylation of R268/R269, we compared the enzymatic activity of WT and R268/269K LDHA before and after in vitro methylation. As expected, WT LDHA exhibited a robust increase in activity following incubation with PRMT1 and SAM, confirming the enhancement shown in Figure S6g. In contrast, the R268/269K mutant showed no significant change in activity under the same conditions, and its post‑methylation activity remained substantially lower than that of methylated WT LDHA (Figure S6j). These results provide direct evidence that PRMT1 enhances LDHA activity specifically via methylation of R268/R269, and that the impaired activity of the R268/269K mutant is attributable to its inability to be methylated at these residues.

To explore how ADMA modification influences LDHA activity, molecular simulations were conducted. MD simulations showed that ADMA modification significantly reduced fluctuations in residues 187–195 compared to the WT LDHA system (Figure 4h). Furthermore, ADMA‐modified R268 and R269 stabilized the α/β complex near LDHA's C‐terminus, particularly the β‐sheet semi‐roll structure (residues 269–304). This stabilization was attributed to the hydrophobic groups at ADMA‐modified R268 and R269 near the LDHA tetramer interface (Figure 4i), causing crowding in the interchain space and altering the dynamics of the semi‐roll structure. Consequently, the stabilization of the LDHA catalytic site at H193 reduced the H193‐NAD^+^ distance (Figure 4j), enhancing catalytic activity. Supporting these findings, the LDHA R268/269A mutant significantly reduced enzymatic activity upon LPS stimulation (Figure 4k). Importantly, the charge‐preserving R268/269K mutant, which retains positive charge but cannot be methylated, similarly displayed markedly decreased enzymatic activity similar to the R268/269A mutant (Figure S6f). Additionally, reconstitution of LDHA‐knockdown THP‐1 cells with wild‐type LDHA restored STAT3 activation, while the R268/269A mutant failed to induce significant activation (Figure 4l).

Although these MD simulations also revealed modestly increased flexibility in two adjacent loop regions—the Active Ring (residues 98–110) and the peripheral Loop2 (residues 220–230)—upon ADMA modification (Figure 4j), we sought to determine whether this enhanced flexibility functionally contributes to upregulated LDHA activity. As shown in Figure S7A, the Active Ring forms part of the catalytic pocket, whereas Loop2 is distant from the active site. To test the functional relevance of increased Active Ring flexibility, we introduced conservative alanine substitutions at flexible, non‐catalytic residues E104 and S105. When expressed in HEK293T cells, these mutants LDHA E104A, LDHA S105A, and LDHA E104A/S105A retained lactate production. Critically, co‐expression of wild‐type PRMT1 enhanced enzymatic activity of all Active Ring mutants to a similar extent as wild‐type LDHA, whereas the methylation‐deficient LDHA R268K/R269K control showed no significant response (Figure S7b,c). Complementary in silico analyses confirmed that ADMA modification preserves key spatial relationships between R99 and both H193 and NAD^+^, while reducing the catalytically favorable H193–NAD^+^ distance (Figure S7d). Radius of gyration calculations further indicated no substantial differences in overall compactness of the Active Ring or Loop2 between wild‐type and ADMA‐modified systems (Figure S7e). Taken together, these findings establish that ADMA modification at residues R268 and R269 is essential for LDHA enzymatic activity and STAT3 activation, with the primary mechanism involving stabilization of the catalytic H193 center rather than secondary changes in adjacent loop flexibility.

To test potential contributions from other PRMTs, we performed siRNA knockdown of *Prmt5* or *Prmt1* (positive control) in peritoneal macrophages, with scrambled siRNA as negative control. Knockdown efficiency was confirmed (Figure S7f). After LPS stimulation, p‐STAT3 (Y705) levels were reduced in *Prmt1* knockdown cells, but unchanged in *Prmt5* knockdown cells (Figure S7f). Parallel knockdown of *PRMT3* or *PRMT1* showed that asymmetric dimethylation of LDHA was reduced only in *PRMT1*‐deficient cells, with no change in *PRMT3*‐deficient cells (Figure S7g). The LDHA R268K/R269K mutant abolished detectable asymmetric dimethylation (Figure S7g) and reduced p‐STAT3 levels compared to wild‐type (Figure S7h). These data indicate that PRMT5 and PRMT3 do not significantly contribute to LDHA asymmetric dimethylation at R268/R269 or STAT3 activation in this model.

### 2.7 Lactylation of STAT3 at K709 Facilitates Its Phosphorylation at Y705

**FIGURE 5:**
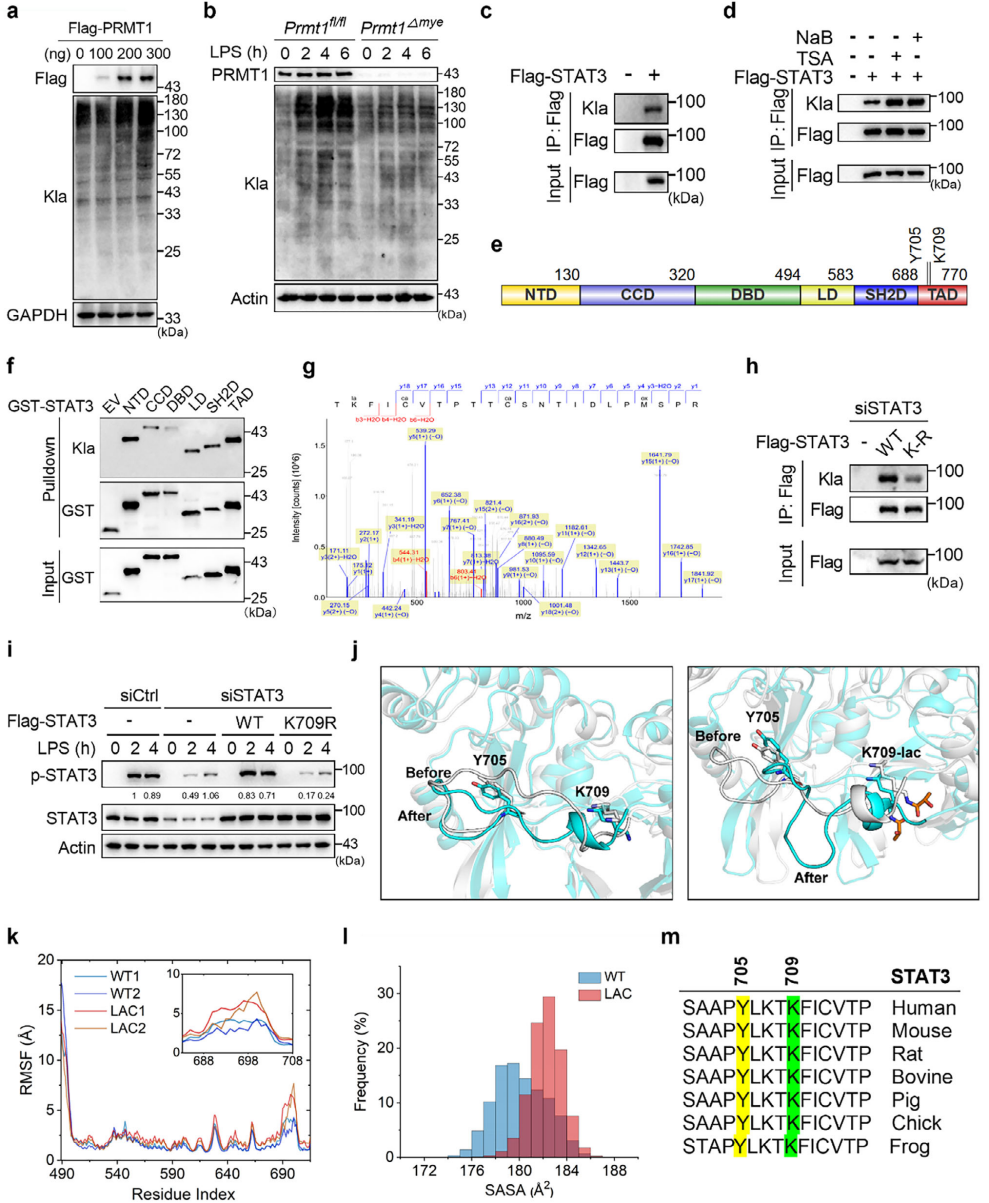
Lactylation of STAT3 at K709 facilitates its phosphorylation at 705. (a,b) Immunoblotting of total lactylation in HEK293T cells transfected with different amounts of Flag tagged PRMT1 expression plasmids (a), or in *Prmt1^fl/fl^* and *Prmt1^Δmye^* BMDMs left untreated or LPS (100 ng/mL) stimulation for the indicated times (b). (c,d) Lactylation of overexpressed STAT3 in HEK293T cells left untreated (c) or treated with sodium butyrate (1 mM) or TSA (100 nM) (d). (e) Schematic of STAT3 domains. (f) Lactylation mapping in STAT3 truncation mutants through pulldown using HEK293T cells. (g) LC‐MS/MS analysis of Flag‐tagged STAT3 TAD identified K709 as the STAT3 lactylation site. (h) Lactylation of reconstituted STAT3 WT or K709R mutant in THP‐1 cells following siRNA‐mediated STAT3 knockdown. (i) Immunoblotting of p‐STAT3 (Y705) in THP‐1 cells treated as follows: siCtrl, siSTAT3, or siSTAT3‐transfected cells rescued with STAT3 WT or K709R, then stimulated with LPS (100 ng/mL) for indicated times. (j) Conformational changes in the STAT3 C‐terminal loop, featuring Y705 and K709 in sticks, are shown for two states: white for pre‐simulation and cyan for post‐simulation. The left panel shows WT, and the right panel shows the lactylation‐modified structure. (k) RMSF curves of the STAT3 homodimer structure under various lactylation modified conditions, with the inset Figure highlighting the fluctuations in residues 685–708, offering a detailed view of the fluctuations surrounding the C‐terminal loop structure in STAT3. (l) The SASA distribution of residue Y705 under different modification conditions is represented with blue columns for the WT and red columns for the lactylation‐modified system. (m) Cross‐species sequence alignment of STAT3 revealed a conserved lactylation site at K709 and a phosphorylation site at Y705. The data presented in (a–d, f, h, i) are representative of three independent experiments. Original blot can be found in Figure S12.

LDHA catalyzes the conversion of pyruvate and NADH to lactate and NAD^+^, with lactyl‐CoA acting as a donor for lysine lactylation (Kla; Figure S8a)—a posttranslational modification implicated in inflammatory and immune regulation [10, 11, 33, 34]. Overexpression of PRMT1 in HEK293T cells enhanced protein Kla in a dose‐dependent manner (Figure 5a). In *Prmt1^fl/fl^* and *Prmt1^Δmye^* BMDMs, LPS stimulation significantly increased total lactylation in *Prmt1^fl/fl^* cells compared to *Prmt1^Δmye^* BMDMs (Figure 5b), indicating PRMT1 as a positive regulator of protein Kla.

To assess whether this effect is specific to lactylation rather than a general influence on related lysine modifications such as acetylation, we examined global acetylation using the same lysates. In HEK293T cells, PRMT1 overexpression did not alter overall protein acetylation in a dose‐dependent manner (Figure S8b). Similarly, neither basal nor LPS‐stimulated global acetylation levels showed consistent or significant differences between *Prmt1^fl/fl^* and *Prmt1^Δmye^* BMDMs (Figure S8c). These results collectively demonstrate that PRMT1 selectively enhances protein lactylation without substantially affecting global lysine acetylation.

To investigate whether PRMT1 enzymatic activity governs Kla, *PRMT1*‐knockdown cells were reconstituted with plasmids encoding dominant‐negative (DN) mutations (G98R, E162Q, E171A, or 63–VLD–65 to AAA), which significantly reduced Kla compared to WT PRMT1 reconstitution (Figure S8d). Similarly, treatment with TC‐E 5003 attenuated total Kla in a dose‐dependent manner upon LPS stimulation (Figure S8e), confirming that PRMT1 enzymatic activity positively regulates protein Kla.

To determine whether STAT3 undergoes lactylation, immunoprecipitation assays identified STAT3 as a lactylated protein (Figure 5c). Lactylation levels of STAT3 were significantly increased upon treatment with sodium butyrate or trichostatin A (TSA; Figure 5d), both they were known to enhance total Kla [35]. STAT3 comprises six domains: N‐terminal, coiled‐coil, DNA‐binding, linker region Src Homology 2 (SH2), and transactivation domains (TAD; Figure 5e) [36]. To map the lactylation sites, we performed truncation mutagenesis followed by anti‐Kla immunoprecipitation. Although multiple truncated constructs exhibited lactylation signals (Figure 5f), we prioritized the transactivation domain (TAD) for further analysis due to its proximity to the critical activation residue Y705. Subsequent LC‐MS/MS identified Lysine 709 (K709) as a lactylation site, with mutagenesis of K709R significantly reducing lactylation (Figure 5g). Reconstitution of K709R or WT STAT3 into THP‐1 STAT3‐knockout cells revealed that the K709R mutation abolished the Kla signal compared to WT STAT3 (Figure 5h), highlighting K709 as a critical lactylation site. Given the close proximity of K709 to Y705, a key phosphorylation site for STAT3 activation, we examined how K709 lactylation influences Y705 phosphorylation. Reconstitution experiments in THP‐1 STAT3‐knockout cells confirmed that the K709R mutation significantly disrupted the STAT3 phosphorylation (Figure 5i), and substantially reduced downstream IL‐10 mRNA expression compared to wild‐type STAT3 reconstitution (Figure S8f).

Consistent with previous reports, STAT3 undergoes acetylation, and treatment with sodium butyrate or trichostatin A increased STAT3 acetylation levels (Figure S8g,h). To exclude potential contributions of acetylation to the functional effects observed at STAT3 K709, we performed immunoprecipitation of wild‐type, charge‐preserving K709R, and acetyl‐mimetic K709Q STAT3 constructs, followed by immunoblotting with pan‐acetyl‐lysine antibody. This analysis revealed no significant differences in overall STAT3 acetylation among the three variants (Figure S8i), indicating that K709 is not a major site of acetylation and that mutations at this residue do not substantially affect global STAT3 acetylation. In parallel reconstitution experiments in *STAT3*‐knockdown THP‐1 cells, both the lactylation‐deficient K709R and the acetyl‐mimetic K709Q mutants failed to restore Y705 phosphorylation to the level achieved with wild‐type STAT3 (Figure S8j). These results were comparable to the impairment previously observed with the K709R mutant (Figure 5i). Thus, disruption of lactylation at K709 impairs STAT3 Y705 phosphorylation, irrespective of charge preservation or mimicry of constitutive acetylation.

MD simulations revealed that lactylation at K709 altered residue properties and disrupted the K709–E690 interaction, causing conformational rearrangements in the loop region (Figure 5j). Fluctuation analyses highlighted dynamic differences in the region encompassing residues 685–708 between WT and lactylated systems (Figure 5k). This conformational shift exposed Y705, increasing its solvent‐accessible surface area (SASA) and making it more susceptible to LDHA modification (Figure 5l). Cross‐species sequence alignment further demonstrated the conservation of K709, underscoring its functional importance (Figure 5m). These findings collectively demonstrate that lactylation of STAT3 at K709 enhances its activation, providing mechanistic insight into the regulation of STAT3 signaling by lactylation.

To further validate that the functional effects are specifically driven by lactylation rather than by the lysine residue itself or potential acetylation at K709, we performed exogenous lactate and acetate treatment experiments in THP‐1 cells. Endogenous *STAT3* was knocked down by siRNA, followed by reconstitution with empty vector, STAT3 WT, or STAT3 K709R. Cells were then treated with lactate, acetate, or vehicle control. As shown in Figures S8k,l, lactate treatment specifically induced STAT3 K709 lactylation, which was required for increased p‐STAT3 (Y705) and IL‐10 expression. Acetate treatment neither induced K709 lactylation nor affected STAT3 activation or IL‐10 production. These findings establish that the functional effects of lactate on STAT3 and IL‐10 are mediated by K709 lactylation, not by the lysine residue itself or alternative acetylation events.

To exclude the possibility that PRMT1 directly methylates STAT3, we immunoprecipitated STAT3 from PRMT1‐overexpressing or *PRMT1*‐knockdown THP‐1 cells and immunoblotted with a pan‐asymmetric dimethylarginine (ADMA) antibody. No ADMA modification was detected on STAT3 under either condition, whereas robust ADMA modification of LDHA was detected in parallel samples as a positive control (Figure S8m,n). Additionally, PRMT1 has been reported to methylate STAT3 at arginine 688 in certain contexts [37]. To determine whether this modification contributes to STAT3 activation in LPS‐stimulated myeloid cells, we knocked down endogenous STAT3 in THP‐1 cells and reconstituted expression with wild‐type or methylation‐resistant R688A mutant STAT3. Following LPS stimulation, the R688A mutation did not reduce Y705 phosphorylation and instead resulted in slightly higher p‐STAT3 levels compared to wild‐type reconstitution (Figure S8o). Consistent with these findings, unbiased proteomic analysis of PRMT1‐interacting proteins in the same cellular context did not identify STAT3 as a high‐confidence interactor (Data S3). Together, these data indicate that PRMT1 does not directly methylate STAT3 in human myeloid cells and support an indirect mechanism of STAT3 activation via PRMT1‐mediated metabolic reprogramming of LDHA.

### 2.8 Blocking STAT3 K709 Lactylation With a Synthesized Peptide Exacerbates DSS‐Induced Colitis

**FIGURE 6:**
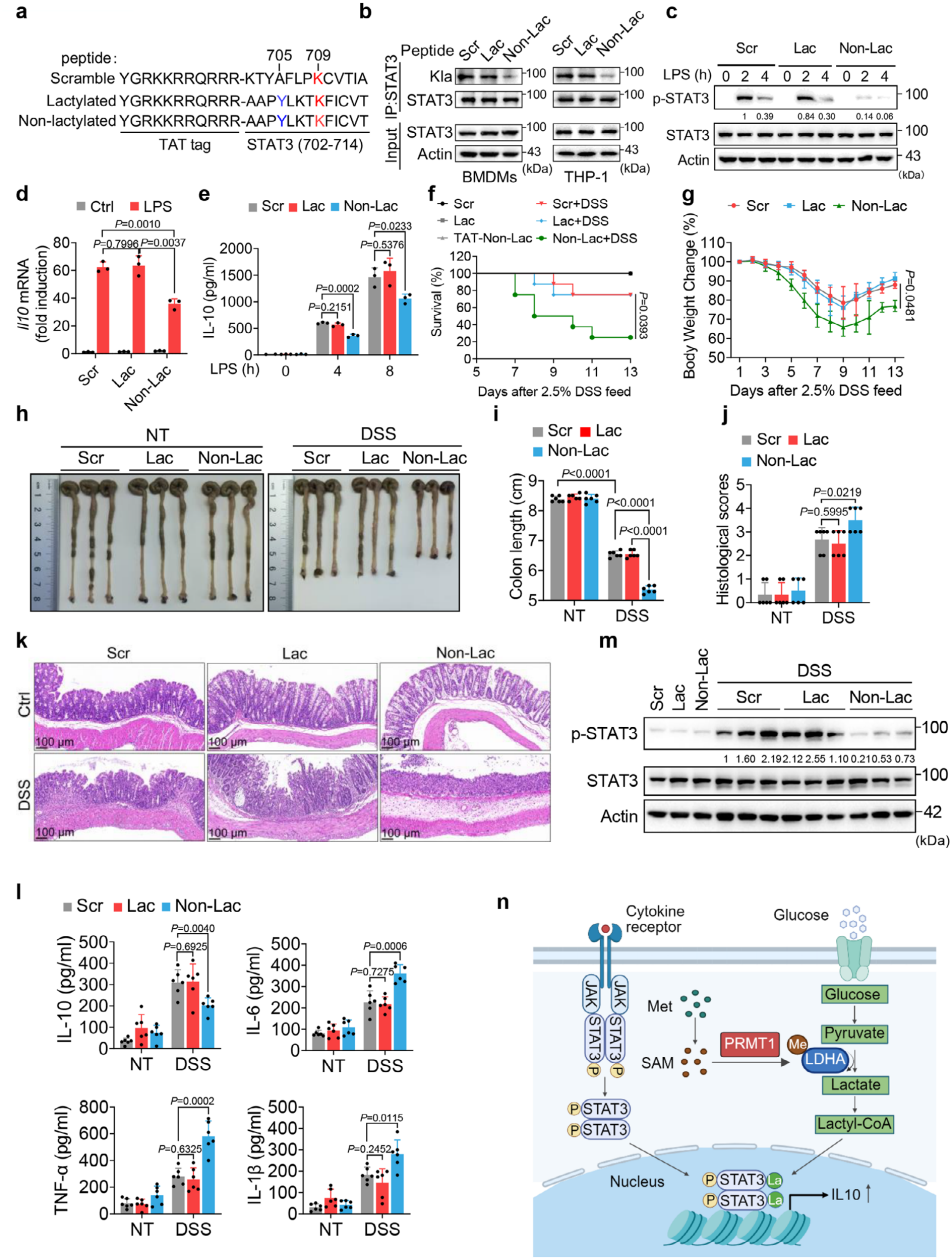
Blocking STAT3 K709 lactylation exacerbates DSS‐induced colitis. (a) Schematic representation of the synthesized cell‐permeable TAT‐tagged STAT3 (702–714) peptides: TAT‐scramble peptide (randomized sequence with identical amino acid composition to the STAT3 702–714 region; sequence: YGRKKRRQRRR‐KTYAFLPKCVTIA), TAT‐non‐lactylated peptide (unmodified at K709), and TAT‐lactylated peptide (K709 lactylated). (b) Lactylation of endogenous STAT3 immunoprecipitated from BMDMs (left) or THP‐1 cells (right) treated with lactylated or non‐lactylated peptides (20 µM) for 24 h. (c–e) Immunoblotting of phosphorylated STAT3 (Y705) and total STAT3 (c), transcripts of *Il10* (d), and supernatant IL‐10 protein levels (e) in cells treated with lactylated or non‐lactylated peptides (20 µM) for 24 h, followed by LPS (100 ng/mL) for the indicated times (*n* = 3). (f–m) C57BL/6J mice were administered intravenous injections via the tail vein with either TAT‐scramble peptide, TAT‐lactylated peptide or TAT non‐lactylated peptide (10 mg/kg) for 3 days, followed by 5 days of 2.5% DSS in drinking water, then 8 days of regular water. Intravenous injections were continued every other day throughout the experimental period. Kaplan–Meier survival analysis (f) (*n* = 8), body weight changes (g) (*n* = 8), representative colon images (h), quantification of colon length (i), and histopathological evaluation of colon tissues with scoring performed in a double‐blind manner (j), H&E staining showing crypt degeneration and immune cell infiltration in colon sections (k), protein levels of IL‐10, IL‐6, TNFα, and IL‐1β in colon explant cultures (l) (*n* = 6), and immunoblotting of phosphorylated STAT3 (Y705) and total STAT3 in colon tissues from C57BL/6J mice treated with TAT‐scramble peptide, TAT‐lactylated peptide, or TAT‐non‐lactylated peptide (m). (n) Schematic representation of the mechanism by which S‐adenosylmethionine regulates STAT3 activation through lactate metabolism. The cartoon was created in BioRender. Hui, W. (2026) https://BioRender.com/k3xohvu. Error bars represent the SD. Statistical significance was determined using unpaired *t*‐test (d, e, g, i, j, l) or Mantel‐Cox test (f). The data presented in (b, c, m) are representative of three independent experiments. *p* < 0.05 is considered statistically significant. Original blot can be found in Figure S13.

Given the pivotal role of PRMT1‐mediated enhancement of STAT3 K709 lactylation in STAT3 activation, we investigated the functional significance of STAT3 K709 lactylation in a DSS‐induced colitis mouse model. STAT3 peptides encompassing amino acids 702–714 were synthesized, including a non‐lactylated peptide at K709 and a K709 lactylated peptide, both tagged with a TAT peptide at the N‐terminus, as well as a TAT‐scrambled peptide with randomized sequence but identical amino acid composition as an additional negative control (Figure 6a). The non‐lactylated peptide demonstrated superior inhibition of K709 lactylation over the lactylated peptide and showed no inhibition with the scrambled peptide (Figure 6b). Mechanistically, the non‐lactylated peptide functions as a competitive inhibitor, outcompeting endogenous STAT3 for the lactyltransferase enzyme(s). This reduces K709 lactylation of native STAT3, which in turn suppresses Y705 phosphorylation and downstream IL‐10 production. The lactylated peptide, being already modified, cannot act as a substrate for the enzyme and therefore does not inhibit lactylation of endogenous STAT3. Furthermore, it significantly reduced STAT3 phosphorylation and IL‐10 production at both transcript and protein levels in response to LPS stimulation, relative to both the lactylated and scrambled control peptides (Figure 6c–e). These findings collectively highlight the potent inhibitory effect of the non‐lactylated peptide on STAT3 activation.

To assess the pro‐inflammatory potential of the non‐lactylated peptide in vivo, its effects were evaluated in a DSS‐induced colitis mouse model. Mice treated with the non‐lactylated peptide exhibited markedly increased mortality (Figure 6f), pronounced weight loss (Figure 6g), reduced colon length (Figure 6h,i), and exacerbated histological damage (Figure 6j) compared to both the lactylated peptide‐treated and scrambled peptide‐treated groups. Histological analysis revealed a significant increase in immune cell infiltration in the non‐lactylated peptide group (Figure 6k). These mice also displayed elevated levels of pro‐inflammatory cytokines, including IL‐6, TNF‐α, and IL‐1β, alongside reduced IL‐10 levels (Figure 6l). Additionally, phosphorylated STAT3 levels were substantially lower in the non‐lactylated peptide‐treated mice than in controls mice (Figure 6m). Together, these findings underscore the critical role of STAT3 K709 lactylation in mitigating DSS‐induced colitis. These results suggest that lactate enhances STAT3 activation and STAT3 lactylation attenuates DSS‐induced colitis, suppression of intestinal inflammation. This study identified a synergistic interaction between amino acid and glucose metabolism, orchestrated by the SAM–PRMT1–LDHA–lactate–lactylation axis, which regulates STAT3 activation and IL‐10 production to modulate intestinal inflammation (Figure 6n).

## 3 Discussion

STAT3 serves as a pivotal regulator of intestinal inflammation and colitis‐associated carcinogenesis, yet the metabolic pathways modulating its activity remain poorly defined. This study uncovers a synergistic interplay between amino acid and glucose metabolism that orchestrates STAT3 activation via the SAM–PRMT1–LDHA–lactate–lactylation axis. LPS stimulation elevates intracellular levels of both SAM and lactate (Figure 1a), directly driving STAT3 activation and enhancing the expression of the anti‐inflammatory cytokine IL‐10. Our work establishes lactylation as a critical post‐translational modification that activates the STAT3–IL‐10 pathway, thereby expanding the known regulatory network governing STAT3.

### 3.1 Lactylation of STAT3 at K709 is Essential for its Activation and Anti‐Inflammatory Function

We demonstrate that lactylation of STAT3 at lysine 709 (K709) in the transactivation domain is essential for robust Y705 phosphorylation and downstream anti‐inflammatory IL‐10 production. Lactylation‐deficient mutants markedly impaired these processes in reconstituted STAT3‐knockout cells, while cell‐permeable competitive peptides selectively blocking K709 lactylation suppressed STAT3 activation in vitro and substantially exacerbated DSS‐induced colitis in vivo. Complementary cellular, biochemical, and physiological experiments, together with evidence of minimal acetylation at this site, collectively establish the critical and specific role of K709 lactylation in promoting anti‐inflammatory STAT3 signaling and attenuating experimental colitis. These observations are consistent with the protective effects of exogenous lactate administration in colitis [38], and its broader roles in barrier repair and immune modulation [39, 40]. Although truncation mapping detected lactylation signals across multiple domains, we prioritized the C‐terminal transactivation domain—known to be critical for Y705 and S727 phosphorylation [41] —due to the close proximity of K709 to Y705, suggesting a potential structural mechanism that facilitates kinase access or phospho‐stabilization. Future studies employing myeloid‐specific conditional manipulation of LDHA and/or STAT3 will provide additional insight into the physiological relevance of this lactylation axis in vivo.

### 3.2 PRMT1 Deficiency Exacerbates Inflammation via NF‐κB Hyperactivation and its Myeloid‐Specific Role

Mechanistically, PRMT1 emerged as the key PRMT family member regulating the STAT3–IL‐10 signaling axis in macrophages, enhancing resistance to colitis (Figure 2). Myeloid‐specific PRMT1 deficiency significantly elevated pro‐inflammatory cytokines via NF‐κB pathway hyperactivation (Figure S2). We established direct causality through multiple complementary approaches: PRMT1 loss led to accelerated IκBα degradation, enhanced p65 phosphorylation and nuclear translocation in LPS‐stimulated BMDMs; increased NF‐κB transcriptional activity in a reporter assay, reversible by PRMT1 re‐expression; and excessive cytokine production largely rescued by pharmacological NF‐κB inhibition with BAY 11–7082. This aligns with prior reports that PRMT1 suppresses NF‐κB via asymmetric dimethylation of RelA/p65 [13, 42]. The cytokine profile in Prmt1*^Δmye^* mice—characterized by a significant yet modest reduction in IL‐10 alongside a dramatic surge in IL‐6—underscores the specialized, non‐redundant role of myeloid‐derived IL‐10 as a gatekeeper of intestinal immune tolerance [43, 44]. Its production, dependent on PRMT1‐mediated STAT3 regulation, operates at a critical threshold. Breach of this checkpoint permits uncontrolled activation of resident immune cells and hyperactivation of pro‐inflammatory pathways, including STAT1 and NF‐κB, culminating in amplified IL‐6 production.

### 3.3 PRMT1 Regulates STAT3 Indirectly by Methylating LDHA, Not Through Direct STAT3 Methylation

Critically, PRMT1 does not directly methylate STAT3 in this context. STAT3 immunoprecipitation followed by pan‐ADMA immunoblotting detected no asymmetric dimethylation, even under PRMT1 overexpression conditions (Figure S8m,n). Consistently, the R688A mutation did not impair IL‐6‐induced Tyr705 phosphorylation and yielded p‐STAT3 levels comparable to or slightly higher than wild‐type STAT3 (Figure S8o). Furthermore, unbiased proteomic profiling failed to identify STAT3 among high‐confidence PRMT1 interactors (Data S3). These findings preclude a significant role for direct PRMT1‐mediated arginine methylation of STAT3.

PRMT1 binds to LDHA, enhancing its activation without affecting its stability, thereby promoting lactate production. Instead, PRMT1 asymmetrically dimethylates LDHA at residues R268/R269, enhancing its catalytic activity and lactate production. The specificity of PRMT1 for this modification in inflammatory macrophages was confirmed by the lack of effect of PRMT5 or PRMT3 knockdown on LDHA asymmetric dimethylation or downstream STAT3 activation (Figure S7f,g), in contrast to prior reports of PRMT5‐mediated regulation via SMAD7 in epithelial cells and PRMT3‐mediated methylation of LDHA at R112 in hepatocellular carcinoma cells [45, 46]. These observations indicate cell type‐ and context‐dependent roles for different PRMT family members.

We attempted to confirm the ADMA modification sites on LDHA by high‐resolution LC‐MS/MS under PRMT1 overexpression and knockdown conditions; however, no modified peptides were detected (Data S5), consistent with the known technical challenges in capturing low‐stoichiometry arginine methylation, including precursor ion competition, absence of methylarginine‐specific enrichment, methyl group lability, and ion suppression in complex mixtures [47, 48, 49].Nevertheless, the functional importance of PRMT1‐dependent arginine methylation at R268/R269 is robustly substantiated by convergent orthogonal lines of evidence: (i) PRMT1‐regulated global ADMA signal on LDHA, (ii) near‐total loss of ADMA in R268/269 mutants, (iii) equivalent catalytic impairment in both methylation‐resistant and charge‐preserving mutants, (iv) molecular dynamics simulations revealing ADMA‐mediated stabilization of the catalytic pocket, and (v) complete failure of the R268/269A or R268/269K mutant to restore STAT3 activation.

The in vitro methylation assay (Figure S6g) shows that PRMT1‐catalyzed methylation enhances LDHA activity. This is supported by the reduced intrinsic activity of the methylation‐resistant R268K/R269K mutant (Figure S6h) and the comparable purity of the wild‐type and mutant proteins (Figure S6i). Moreover, direct comparison of WT and R268/269K activity before and after in vitro methylation (Figure S6j) revealed that only WT, not the methylation‐dead mutant, responds to PRMT1 treatment with increased activity. Although we did not detect ADMA‐modified peptides by mass spectrometry—likely due to low methylation stoichiometry and technical limitations of conventional DDA‐MS—the cumulative functional evidence from multiple orthogonal approaches strongly supports that PRMT1 methylates LDHA at R268/R269 to promote its enzymatic function, thereby reinforcing the proposed metabolic‐inflammatory axis.

### 3.4 Lactate Regulates STAT3 via Specific Lactylation, Not Acetylation

The resultant lactate regulates STAT3 through site‐specific lactylation, not acetylation. PRMT1 manipulation did not alter global protein acetylation levels (Figure S8b,c), and STAT3 K709 was not a predominant acetylation site (Figure S8i). Both the charge‐preserving K709R and acetyl‐mimetic K709Q mutants, which prevent lactylation, exhibited similar deficits in restoring Y705 phosphorylation (Figure S8j), establishing lactylation as the essential mechanism. This refines the paradigm that global lactylation promotes anti‐inflammatory responses [10, 11, 33, 34] by identifying a precise, functional node on STAT3. The anti‐inflammatory effects of histone deacetylase inhibitors like sodium butyrate and trichostatin A may involve concurrent enhancement of both acetylation and lactylation (Figure 5c,d).

### 3.5 Validation in Human IBD Tissue and Clinical Implications

Multiplex immunofluorescence analysis of human IBD colon mucosa validated the PRMT1‐mediated metabolic‐inflammatory axis identified in preclinical models. In CD68^+^ macrophages, PRMT1 and LDHA showed cytoplasmic co‐localization, while phospho‐STAT3 (Y705) was predominantly nuclear with minimal overlap with PRMT1, supporting an indirect mechanism whereby PRMT1 stabilizes LDHA to enhance glycolysis and subsequent STAT3 phosphorylation/nuclear translocation. Complementary analysis within the same cohort revealed coordinated upregulation of PRMT1, phospho‐LDHA (Y10), phospho‐STAT3 (Y705), and IL‐10 in active disease mucosa, with signal intensities correlating with clinical and histologic activity scores. This links PRMT1‐driven metabolic reprogramming to STAT3 activation and IL‐10 expression in vivo, underscoring the pathway's clinical relevance.

## 4 Conclusion

In conclusion, we delineate a metabolic‐transcriptional circuit wherein SAM fuels PRMT1‐mediated LDHA activation, increased lactate production drives STAT3 K709 lactylation and phosphorylation, and ultimately enhances IL‐10 transcription. This pathway represents a promising therapeutic target for inflammatory bowel disease.

## 5 Methods

### 5.1 Mice

*Prmt1^fl/fl^* mice on a C57BL6/J background were generously provided by Dr. Shilai Bao (Institute of Genetics and Developmental Biology, Chinese Academy of Sciences, China). *Lyz2*‐Cre mice (004781) were obtained from Jackson Laboratories. *Prmt1^Δmye^* mice were generated by crossing *Prmt1^fl/fl^* mice with *Lyz2*‐Cre mice. All mice were housed in standard specific‐pathogen‐free conditions at 22°C with circulating air, a constant humidity of 60 ± 10%, and a 12 h light/dark cycle. Mice were fed a standard diet and had ad libitum access to sterilized water. Age‐ and sex‐matched mice, 6–8 weeks old, were used in all experiments. Genotyping of *Prmt1^fl/fl^* mice was confirmed by polymerase chain reaction PCR) assay using the following primers: Forward 5′‐GTGCTTGCCATACAAGAGATCC‐3′ and Reverse 5′‐ACAGCCGAGTAGCAAGGAGG‐3′. PCR conditions included an initial denaturation at 94°C for 5 min, followed by 35 cycles of 94°C for 30 s, 62°C for 30 s, and 72°C for 30 s. For TAT‐peptide treatment, TAT‐scramble, TAT‐lactylated or TAT‐nonlactylated peptides (10 mg/kg) were administered via intravenous tail vein injections every other day. Euthanasia was performed using CO_2_ inhalation at a flow rate of 30%–70% of the chamber volume per minute, followed by cervical dislocation to confirm death. Carcasses were stored at −20°C post‐euthanasia. Mice were euthanized if their weight loss exceeded 20%. All animal experiments were performed according to protocols approved by the Animal Care and Use Committee of Shandong Normal University (AEECSDNU2021048).

### 5.2 Cell Culture and Stimulation

HEK293T (ATCC CRL‐11268) and L929 (ATCC CCL‐1) cells were obtained from ATCC and cultured in a humidified atmosphere (5% CO_2_, 37°C) in Dulbecco's Modified Eagle's Medium (DMEM, Macgene, CM15019) supplemented with 10% fetal bovine serum (FBS, 40130ES76, Yeasen). THP‐1 cells (SCSP‐567, ATCC) and HT‐29 (ATCC HTB‐38) were maintained in RPMI 1640 medium (CM10041, Macgene) with 10% FBS. BMDMs were generated from *Prmt1^fl/fl^*, *Prmt1^Δmye^*, or WT mice using L929‐conditioned medium. To generate BMDMs, cells were cultured in L929 cell–conditioned DMEM/F‐12 (CM10090, Macgene) supplemented with 10% FBS, 1% nonessential amino acids (C0332, Beyotime), and 1% penicillin‐streptomycin (ST488, Beyotime) for 5 days as previously described. Macrophages were stimulated with LPS (100 ng/mL, tlrl‐peklps, Invivogen) or IL‐6 (20 ng/mL, Ag23467, proteintech) for varying durations, as detailed in Figure legends. Cell culture supernatants were used for enzyme‐linked immunosorbent assay ELISA), while cells were harvested for RT‐PCR or immunoblotting. The compounds used in cell experiments include PF9366 (72882‐78‐1, aladdin), AMI 1 (20324‐87‐2, aladdin), MS023 (C3503, Apexbio), TC‐E 5003 (B7750, Apexbio), Cycloheximide (CHX, A356844, Sangon Biotech, Taiwan), oxamic acid sodium (OAS, 565‐73‐1, MCE), sodium butyrate (NaB, B5887, Sigma‐Aldrich), Trichostatin A (TSA, A8183, Apexbio), BAY 11–7082 (19542‐67‐7, yuanye, shanghai), lactate (867‐56‐1, Sigma‐Aldrich), acetate (127‐09‐3, Psaitong).

### 5.3 Cell Transfection

HEK293T and THP‐1 cells were transfected for 30 h with specified expression plasmids using Liposomal Transfection Reagent (40802ES03, Yeasen Biotechnology). Immunoprecipitation and immunoblotting assays were performed afterward. Gene knockdown was achieved by transfecting siRNAs (Tsingke, beijing, China) using INTERFERin siRNA transfection reagent (101000028, PolyPlus, France) according to the manufacturer's protocol. siRNA sequences are provided in Table S1.

### 5.4 Quantitative Reverse Transcription PCR, RT‐qPCR

Total RNA was extracted from cultured cells using Trizol reagent (R401‐01, Vazyme) and reverse‐transcribed into cDNA with HiScript II Q RT SuperMix for qPCR (R223‐01, Vazyme). Quantitative PCR (qPCR) was conducted using SYBR Green PCR Master Mix (11201ES08, Yeasen Biotechnology) on a LightCycler 480 II System (Roche). Fold changes in mRNA expression were calculated using the ΔΔCt method, with β‐actin as the internal control. Primer sequences are listed in Table S2.

### 5.5 ELISA

Cytokine levels in cultured BMDMs and colon explants were measured using ELISA kits for mouse IL‐10 (4311411), IL‐1β (432601), IL‐6 (431301), and TNF‐α (430901) from BioLegend, following the manufacturer's protocols.

### 5.6 Intracellular LDH Activity Measurement

LDH activity was measured using a previously described method [50]. Briefly, LDH activity was quantified by monitoring NADH oxidation in a reaction mixture containing 20 mM HEPES (pH 7.2), 20 µM NADH, 0.05% bovine serum albumin, and 2 mM pyruvate. Fluorescence was recorded using a microplate reader at excitation/emission wavelengths of 340/460 nm.

### 5.7 Plasmids and Molecular Cloning

Expression plasmids for LDHA were obtained from WZ Biosciences. Constructs for STAT3 truncations and mutants as well as PRMT1 and LDHA mutants were generated using the ClonExpress II One Step Cloning Kit (C112‐01, Vazyme) and the Mut Express MultiS Fast Mutagenesis Kit (C215‐01, Vazyme), following the manufacturers’ protocols. The nucleotide sequences of all PRMT1, LDHA, and STAT3 mutants and truncations were confirmed through sequencing. The primers used for cloning are listed in Table S3. The Primer sequences for site‐directed mutagenesis are listed in Table S4. The nucleotide sequences of all PRMT1, LDHA, and STAT3 mutants were fully verified by sequencing.

### 5.8 Lentiviral Transfer Plasmids Construction and Transduction

A short hairpin RNA sequence (5′‐CCGGCAGTACAAAGACTACAA‐3′) targeting human PRMT1 was inserted into the pPLK‐GFP‐Puro vector. Lentiviruses carrying gene‐specific or empty vectors were packaged in HEK293T cells co‐transfected with pMDL and VSV‐G. THP‐1 cells were transduced with the lentiviral vectors in the presence of polybrene (H9268, Sigma‐Aldrich), and single‐cell clones were generated via limiting dilution. PRMT1‐deleted cells were subsequently used for downstream assays.

### 5.9 Luciferase Reporter Assay for STAT3 Transcriptional Activity

THP‐1 cells were transfected with a STAT3 luciferase reporter plasmid alongside individual cDNA plasmids using Liposomal Transfection Reagent (40802ES03, Yeasen Biotechnology). Where indicated, cells were stimulated with LPS. After 24 h of transfection and an additional 10‐h LPS stimulation, luciferase activity was quantified using the Firefly Luciferase Reporter Gene Assay Kit (RG006, Beyotime) to measure STAT3‐dependent transcriptional activity.

### 5.10 Colitis and CAC Models

The induction of acute colitis by DSS and colitis‐associated cancer (CAC) by AOM + DSS followed previously described protocols [51, 52]. For the DSS‐induced colitis model, age‐ and sex‐matched mice were exposed to 2.5% DSS (MB5535, Meilun Bio) for 5 days, followed by regular water. Mice were sacrificed on day 7, and inflammation assessments were performed. For the CAC model, mice received an intraperitoneal injection of AOM (10 mg/kg, 25843‐45‐2, MP), followed by three cycles of DSS. Tumor evaluations were performed on day 62. Colon length and histopathological assessments were conducted for each mouse at the end of the study. Bleeding and diarrhea scores were calculated as described, with each parameter rated on a 0–4 scale [53, 54]. Histological evaluations of colitis severity were performed in a double‐blinded manner after H&E staining.

### 5.11 Metabolomic Analysis

A total of 1 × 10^7^ BMDMs derived from wild‐type C57BL/6J mice were treated with or without LPS for 4 h before metabolomic analysis. Metabolite extraction was performed by adding 1 mL of cold methanol:acetonitrile: H_2_O (2:2:1, *v*/*v*/*v*) to 100 µL of each sample. The mixture was vortexed for 60 s, ultrasonicated twice for 30 min at 4°C, incubated at −20°C for 1 h, and centrifuged at 14 000 × *g* for 20 min at 4°C. The supernatant was transferred to new tubes for HILIC LC–MS/MS analysis using an AB5500 QqQ mass spectrometer (AB Sciex, USA) coupled with a Waters I‐class HPLC system (Waters, Ireland). Data acquisition and analysis were conducted by Shanghai Applied Protein Technology Co., Ltd. The list of identified metabolites is provided in Data S1. The relative amount of SAM was calculated using the metabolomic analysis data.

### 5.12 Library Construction for RNA Sequencing and Sequencing Procedures

Total RNA was extracted using the RNeasy Mini Kit (Qiagen) according to the manufacturer's protocol. Paired‐end libraries were constructed with the TruSeq RNA Sample Preparation Kit (Illumina, USA). Briefly, polyA‐containing mRNA was isolated using poly‐T oligo‐attached magnetic beads and fragmented at 94°C for 8 min using divalent cations. The RNA fragments were then reverse‐transcribed into first‐strand cDNA with random primers, followed by second‐strand cDNA synthesis using DNA Polymerase I and RNase H. The resulting cDNA fragments underwent end‐repair, single ‘A’ base addition, and adapter ligation. The final cDNA library was purified, PCR‐amplified, and quantified using a Qubit 2.0 Fluorometer (Life Technologies, USA). Insert sizes and molar concentrations were confirmed using an Agilent 2100 Bioanalyzer (Agilent Technologies, USA). Libraries were diluted to 10 pM, and sample clusters were generated using the cBot system before sequencing on the Illumina HiSeq XTen platform (Illumina, USA). Library construction and sequencing were conducted by the Shanghai Biotechnology Corporation. Gene data is provided in Data S2.

### 5.13 Mass Spectrometry Analysis

PRMT1‐binding proteins were identified through co‐immunoprecipitation (co‐IP) and mass spectrometry (MS). HEK293T cells expressing Flag‐tagged PRMT1 were lysed, and immunoprecipitation was performed using Anti‐DYKDDDDK (Flag) Affinity Gel (20585ES08, Yeasen). The immunoprecipitates were separated via SDS‐PAGE and visualized using Coomassie Blue G‐250 staining. Protein bands were excised, subjected to in‐gel digestion with sequencing‐grade trypsin (Promega), and analyzed on a Q Exactive Plus Orbitrap LC‐MS/MS System (ThermoFisher Scientific). Proteins were identified using Proteome Discoverer 1.2 software (ThermoFisher Scientific). Potential PRMT1 interactors are listed in Data S3.

To identify lactylation sites on STAT3, GST‐tagged STAT3 TAD (Transactivation Domain) plasmids were overexpressed in HEK293T cells and purified using glutathione beads. The immunoprecipitated complex was separated by SDS‐PAGE and visualized with Coomassie Blue G‐250 staining. The GST‐tagged STAT3 TAD protein band was excised, digested in‐gel with α‐lytic protease (MilliporeSigma, A6362), and analyzed by LC‐MS/MS in collaboration with PTM Biolabs (Hangzhou, China).

### 5.14 Molecular Simulation

Structural analyses of LDHA and PRMT1 utilized Protein Data Bank (PDB) entries: LDHA (PDB ID: 6BAD, chains A‐D) and PRMT1 (PDB ID: 6NT2, chain A, residues 42–371). The STAT3 C‐terminal homodimer structure, encompassing the linker and SH2 domains (residues 489–715), was modeled using Modeller 9.10. Missing loops in the crystal structure (PDB ID: 6QHD) were filled using AlphaFold2‐derived structures and refined through 100 ns molecular dynamics (MD) simulations. Protein‐protein interactions between LDHA and PRMT1 were predicted using HDOCK software and relaxed by 100 ns MD simulations. Modified structures were similarly subjected to 100 ns MD simulations to refine conformations.

All MD simulations were conducted with Amber22 using the Amber14SB force field. Systems were solvated in a cubic TIP3P water box with a 1 nm buffer and neutralized with sodium ions. The temperature was gradually increased to 300 K over 100 ps, followed by 10 ns of NVT equilibration and 10 ns of NPT equilibration after four energy minimization steps. Production MD simulations were run at 300 K and 1 atm with the LINCS algorithm restraining hydrogen bonds, enabling a 2‐fs integration time step. Energy and coordinate data were recorded every 10 ps for post‐simulation analysis. All computations were performed on a high‐performance Linux‐based computing cluster.

### 5.15 Coimmunoprecipitation and Immunoblot Analysis

For transient transfection and coimmunoprecipitation experiments, HEK293 cells (1 × 10^6^) were transfected for 36 h. The transfected cells were lysed in radioimmunoprecipitation assay (RIPA) buffer. Cell lysates were centrifuged and the supernatants were incubated with anti‐Flag‐beads overnight at 4°C. For endogenous coimmunoprecipitation experiments, BMDMs stimulated with LPS (100 ng/mL) for 2 h or left untreated. Cell lysates were centrifuged and incubated with anti‐PRMT1 antibody or IgG overnight at 4°C, and then add Protein A/G beads (36403ES03, Yeasen) and incubate at room temperature for 30 min. Beads were washed three times with 1 mL RIPA buffer. The precipitates were analyzed by standard immunoblot procedures.

For immunoblot analysis, cell lysates were prepared using RIPA buffer, and proteins were resolved by SDS‐PAGE (20325ES62, Yeasen Biotechnology) following the manufacturer's protocol. Proteins were transferred to nitrocellulose membranes (Bio‐Rad) and incubated with primary antibodies, followed by HRP‐conjugated secondary antibodies. Detection was performed using an Enhanced Chemiluminescence Reagent (36222ES76, Yeasen Biotechnology), and images were captured using a Tanon Chemiluminescence Imaging System. Antibody details, including dilutions and catalog numbers, are provided in Data S4.

### 5.16 Hematoxylin and Eosin (H&E) Staining of Colon Tissue

Colon tissue sections were prepared following Servicebio's standard operating procedures (Wuhan, China), encompassing pathological tissue sampling, fixation, embedding, and paraffin sectioning. The paraffin sections were sequentially immersed in the following solutions: Environmental‐Friendly Dewaxing Transparent Liquid I and II (20 min each), anhydrous ethanol I and II (5 min each), and 75% ethanol (5 min), followed by rinsing with tap water. The sections were then treated with high‐definition constant staining pretreatment solution for 1 min, stained with hematoxylin solution for 3–5 min, and rinsed. Differentiation was performed using hematoxylin differentiation solution, followed by rinsing and treatment with hematoxylin bluing solution. After rinsing, sections were immersed in 95% ethanol for 1 min, stained with eosin for 15 s, and dehydrated sequentially in absolute ethanol I, II, and III (2 min each), normal butanol I and II (2 min each), and xylene I and II (2 min each). Finally, sections were sealed with neutral gum. Microscopic inspection, image acquisition, and analysis were performed. The samples were evaluated by experienced pathologists who were blinded to each other. The histological score for colon injury was evaluated based on the parameters shown in Table S5.

### 5.17 Mouse Intestinal Lamina Propria Cell Extraction and Flow Cytometry Analysis

Mice were sacrificed, and their colons were excised and longitudinally incised. After removing feces and mucus at room temperature, the tissue was cut into 1 cm fragments and digested with 15 mL of digestive solution 1 (10 mM EDTA, 20 mM HEPES in phosphate‐buffered saline) for 30 min to dissociate epithelial cells. After vigorous shaking, the tissue was filtered and washed with RPMI‐1640 complete medium. Digestive solution 2 (Collagenase VIII (C2139, Sigma‐Aldrich)/DNase I (9003‐98‐9, Psaitong) in RPMI‐1640) was added, and the mixture was incubated at 37°C for 1.5 h. The digested tissue was filtered, centrifuged at 2500 rpm for 5 min, and the middle‐layer cells were isolated using Percoll density gradient centrifugation. These cells were blocked at room temperature for 10 min and stained with antibodies against CD11b, CD45, and F4/80 at 4°C for 30 min. After fixation with 4% paraformaldehyde (BL539A, Biosharp) for 30 min and permeabilization with 0.3% Triton X‐100 for 10 min, the cells were incubated with a primary p‐STAT3 antibody for 1 h. Secondary antibodies were applied after washing, and the cells were resuspended in staining buffer for flow cytometry analysis.

### 5.18 TAT‐Tagged Peptide Synthesis

A synthesized peptide (AAPYLKTKFICVT), corresponding to amino acids 702–714 of STAT3, was conjugated to a TAT cell‐penetrating peptide at the N‐terminus to enhance cellular uptake. In the lactylated peptide, K709 was modified with a lactyl group, whereas K709 remained unmodified in the non‐lactylated peptide. Additionally, a scrambled‑sequence control peptide (maintaining the same amino acid composition as the STAT3 (702–714); sequence: KTYAFLPKCVTIA) was synthesized with the same N‑terminal TAT motif. All peptides were synthesized by APeptide Co., Ltd. (Shanghai, China) and purified to >95% purity using HPLC.

### 5.19 In Vitro Th17 Cell Differentiation From Naïve CD4^+^ T Cells

To induce Th17 polarization in vitro, 48‐well plates were coated with anti‐mouse CD3ε antibody (clone 145‐2C11, cat. no. 16‐0031‐86, Invitrogen) at 1 µg/mL and anti‐mouse CD28 antibody (clone 37.51, cat. no. 16‐0281‐86, Invitrogen) at 2 µg/mL in DPBS (200 µL/well) overnight at 4°C or for 2 h at 37°C; single‐cell suspensions were prepared aseptically from spleens and lymph nodes of *Prmt1^fl/fl^* and *Prmt1^Δmye^* mice, and naïve CD4^+^ T cells (CD4^+^ CD44^−^ CD62L^+^) were purified by fluorescence‐activated cell sorting (FACS), washed, counted, and resuspended in complete RPMI‐1640 medium (supplemented with 10% heat‐inactivated FBS, 100 U/mL penicillin‐streptomycin, and 50 µM β‐mercaptoethanol) at 3 × 10^5^ cells/mL, followed by supplementation with recombinant murine TGF‐β1 (CK33, Novoprotein) at 1 ng/mL, recombinant murine IL‐6 (AF‐216‐16, PeproTech) at 20 ng/mL, anti‐mouse IFN‐γ neutralizing antibody (14‐7311‐85, eBioscience) at 5 µg/mL, and anti‐mouse IL‐4 neutralizing antibody (14‐7041‐85, eBioscience) at 5 µg/mL; after removing unbound antibodies from the coated plates, 1 mL of the cytokine‐supplemented cell suspension (3 × 10^5^ cells/well) was added to each well and cultured for 5 days at 37°C in a 5% CO_2_ incubator without medium change. Th17 polarization efficiency was assessed by quantifying IL‐17A concentration in the culture supernatants using a commercial Mouse IL‐17A ELISA Kit (HJ187, Epizyme) according to the manufacturer's instructions.

### 5.20 Mass Spectrometry Analysis of LDHA Arginine Dimethylation

HEK293T cells were transfected to generate three experimental groups—(i) co‐expression of Flag‐tagged LDHA with wild‐type PRMT1, (ii) Flag‐LDHA expression in a *PRMT1*‐knockdown background via siRNA, and (iii) Flag‐LDHA with empty vector control—after which Flag‐LDHA was immunoprecipitated under denaturing conditions, separated by SDS‐PAGE, and stained with Coomassie Brilliant Blue; the LDHA‐containing gel bands were then excised and subjected to in‐gel trypsin digestion, during which gel slices were destained with 200–400 µL of 30% acetonitrile in 100 mM NH_4_HCO_3_ until transparent, dehydrated with 100% acetonitrile, reduced with 10 mM dithiothreitol at 37°C for 60 min, alkylated with 60 mM iodoacetamide at room temperature in the dark for 60 min, sequentially washed with 100 mM NH_4_HCO_3_ (twice, 15 min each) and 100% acetonitrile, lyophilized, and digested overnight at 37°C with 2.5–10 ng/µL sequencing‐grade trypsin, followed by peptide extraction with 60% acetonitrile/0.1% trifluoroacetic acid under ultrasonication for 15 min, pooling, lyophilization, desalting, and resuspension in 20 µL 0.1% formic acid; no enrichment for arginine‐methylated peptides was performed at any stage. The resulting peptides were separated on a nanoElute HPLC system (Bruker) at 300 nL/min using a Thermo Scientific EASY column (15 cm × 150 µm ID, 2 µm, C18‐A2) with solvent A (0.1% formic acid in water) and solvent B (0.1% formic acid in 99.9% acetonitrile), employing a 30‐min gradient of 0–18 min, 5%–35% B; 18–20 min, 35%–80% B; and 20–30 min, 80% B, after which mass spectrometry was conducted on a timsTOF Pro instrument (Bruker) in positive ion mode at an electrospray voltage of 1.5 kV, acquiring data in parallel accumulation–serial fragmentation (PASEF) mode over m/z 100–1700 with 1 MS scan followed by 8 PASEF MS/MS scans per cycle (0.95 s cycle time, charge range 0–5, active exclusion 24 s); raw data were finally searched using MaxQuant (version 1.6.14) against the human UniProt reference proteome database, specifying Trypsin/P with up to 2 missed cleavages, carbamidomethylation (C) as fixed modification, and oxidation (M), protein N‐terminal acetylation, and asymmetric dimethylarginine (R) as variable modifications, with precursor and fragment mass tolerances of 20 ppm and 0.1 Da, respectively, peptide and protein FDR set to 0.01, and intensity‐based absolute quantification (iBAQ) enabled. Mass spectrometry analysis was performed in collaboration with Applied Protein Technology (Shanghai, China). No ADMA‑modified peptides derived from LDHA were identified under any condition (Data S5).

For in vitro methylated LDHA, recombinant human wild‑type LDHA (N‑terminal 10× His tag, C‑terminal Myc tag) was divided into three groups: a control group (without PRMT1 and SAM), in vitro methylation group 1 (2.5 µg LDHA, 1 µg PRMT1, 100 µM SAM), and in vitro methylation group 2 (5 µg LDHA, 2 µg PRMT1, 100 µM SAM). All reactions were performed in methylation buffer (50 mM Tris‑HCl pH 8.0, 100 mM NaCl, 5 mM MgCl_2_, 1 mM DTT, 0.1 mM PMSF) at 30 °C for 4 h. Subsequently, LDHA was purified using BeyoMag IDA‑Ni magnetic agarose beads, eluted, and buffer‑exchanged. The methylation status of LDHA in the three groups was analyzed by mass spectrometry as described above. No ADMA‑modified peptides derived from LDHA were identified under any condition (Data S6).

### 5.21 In Vitro Methylation and LDHA Activity Assay

Recombinant human wild‑type LDHA (N‑terminal 10× His tag, C‑terminal Myc tag; Cusabio, CSB‑EP012832HU), R268K/R269K mutant LDHA (Cusabio, CSB‑EP012832HU(M)), and active recombinant human full‑length PRMT1 (N‑terminal GST tag; Sino Biological, P365‑380G) were used. Methylation reactions (50 µL) containing 5 µg LDHA, 2 µg PRMT1, and 100 µM SAM (Aladdin Scientific, S192607) were carried out in methylation buffer (50 mM Tris‑HCl pH 8.0, 100 mM NaCl, 5 mM MgCl_2_, 1 mM DTT, 0.1 mM PMSF). Control reactions were set up without PRMT1 or without SAM. After incubation at 30°C for 2 h, LDHA was purified using BeyoMag IDA‑Ni magnetic agarose beads (Beyotime, P2239), eluted, and buffer‑exchanged. LDHA activity was measured as previously described [50]. Briefly, LDH activity was determined by measuring the rate of NADH oxidation in a reaction mixture containing 20 mM HEPES (pH 7.2), 20 µM NADH, 0.05% bovine serum albumin, and 2 mM pyruvate. Fluorescence was measured using a microplate reader (excitation wavelength: 340 nm, emission wavelength: 460 nm). For comparison of wild‑type and mutant LDHA, both proteins were normalized to equal concentrations (0.1 µg/µL) and assayed under identical conditions. Protein purity and equal loading were verified by SDS‑PAGE followed by Coomassie Brilliant Blue staining. For the ‘before vs. after’ methylation comparison, three reaction mixtures were prepared for each protein (WT or R268/269K): one containing PRMT1 and SAM (methylated), a second without PRMT1, and a third without SAM (both mock‑treated). After re‑purification using BeyoMag IDA‑Ni magnetic agarose beads, LDHA activity was measured as described above.

### 5.22 GST Pull‐Down Assay

GST‑tagged PRMT1 (0.5 µg; Sino Biological, P365‑380G) or GST alone (0.5 µg) was immobilized on glutathione‑Sepharose beads (20507ES10, Yeasen) for 2 h at 4°C. After blocking with 1% BSA, the beads were incubated with purified wild‑type LDHA (0.5 µg; Cusabio, CSB‑EP012832HU; N‑terminal 10× His tag, C‑terminal Myc tag) in binding buffer (50 mM Tris‑HCl, pH 7.5, 150 mM NaCl, 0.1% NP‑40, 1 mM DTT) for 2 h at 4°C. The beads were then washed five times with binding buffer, and bound proteins were eluted by boiling in SDS sample buffer. Eluted proteins were analyzed by Western blot using anti‑His antibody (Proteintech, 66005‑1‑Ig) and anti‑GST antibody (ABclonal, AE001).

### 5.23 Tyramide Signal Amplification Multiplex Immunofluorescence

Paraffin‐embedded tissue sections were deparaffinized in xylene (2 × 10 min) and rehydrated through a graded ethanol series (100%, 95%, 85%, 75%; 5 min each), followed by rinsing in distilled water. Antigen retrieval was performed by microwave heating in either pH 8.0 EDTA buffer or pH 6.0 citrate buffer (medium power 8 min, pause 8 min, medium‐low power 7 min), followed by cooling to room temperature and washing in PBS (pH 7.4; 3 × 5 min). Endogenous peroxidase activity was quenched with 3% hydrogen peroxide for 15 min at room temperature. Sections were washed in PBST (PBS containing 0.1% Triton X‐100; 3 × 5 min) and blocked with 10% normal goat serum in PBST for 30 min at room temperature. Primary antibodies were incubated overnight at 4°C: anti‐PRMT1 (1:200), anti‐LDHA (1:200), anti‐phospho‐STAT3 (Tyr705) (1:500), anti‐CD68 (1:500), and anti‐IL‐10 (1:300), as appropriate for the target panel. Sections were then washed in PBST (3 × 5 min) and incubated with HRP‐conjugated polymer secondary antibody for 30 min at room temperature, followed by PBST washes (5 × 5 min). Multiplex TSA was performed sequentially using Opal 570, Opal 520, Opal 620, and Opal 490 tyramide reagents (5 min each). After each TSA cycle, antibodies were stripped by repeating the microwave antigen retrieval step. Following the final cycle and PBST washes, nuclei were counterstained with DAPI (10 min at room temperature, protected from light). Slides were mounted with anti‐fade mounting medium. Images were acquired using confocal microscopy or multispectral imaging.

### 5.24 Statistical Analysis

The statistical methods and sample sizes (*n*) are provided in the Figure legends. Statistical analysis was performed using GraphPad Prism 8.2.1 software. Data were presented as mean ± SD. For in vivo studies, “*n*” refers to the number of mice per group, while for in vitro studies, “*n*” indicates the number of biological replicates. Statistical significance was assessed using unpaired *t*‐tests, one‐way ANOVA or Mantel‐Cox tests, with a threshold of *p* < 0.05.

## Funding

This work was supported by grants from the Natural Science Foundation of China (82341078 to T.L.), SIP High‐Quality Innovation Platform for Chronic Diseases (YZCXPT2022203).

## Conflicts of Interest

The authors declare no conflicts of interest.

## Author Contributions

T.L. and Y.L. designed the experiments, supervised the study and interpreted the data. H.W., M.Z., W.G., and J.Z. performed experiments and provided intellectual input. S.W., J.W., and Y. L. performed computational modeling of PRMT1–LDHA interaction and STAT3 lactylation. W.Z. and W.G. performed biostatistical analyses and generated critical reagents. K.W., C.H., and J.Z. contributed intellectual input. T.L. and Y.L. wrote the manuscript.

## Supporting information




**Supporting File**: advs75934‐sup‐0001‐SuppMat.pdf.

## Data Availability

All data are included in the Supplementary Information or available from the authors, as are unique reagents used in this Article. The raw numbers for charts and graphs are available in the Source Data file whenever possible.
