# Supplementary material for: PRMT1‐Mediated LDHA Methylation Drives STAT3 Lactylation to Orchestrate Intestinal Inflammation and Tumorigenesis
Source: Adv Sci (Weinh). 2026 Jun 1;13(48):e16577. doi: 10.1002/advs.202516577 (PMC13337045; doi:10.1002/advs.202516577)
Supplement: Supplementary file 1 [file ADVS-13-e16577-s001.pdf]

Supporting Information

Title: PRMT1-Mediated LDHA Methylation Drives STAT3 Lactylation to Orchestrate Intestinal Inflammation and Tumorigenesis

Hui Wang, Mengyu Zhang, Weipeng Gong, Jiaxuan Wu, Junping Zhang, Wenxiu Zhang, Yue Liu, Kui Wang, Canhua Huang, Jun Zhou, Sijin Wu, Yan Li* and Tianliang Li**

Supplementary Materials for this manuscript include the following:

Figures. S1 to S20

Tables S1 to S5

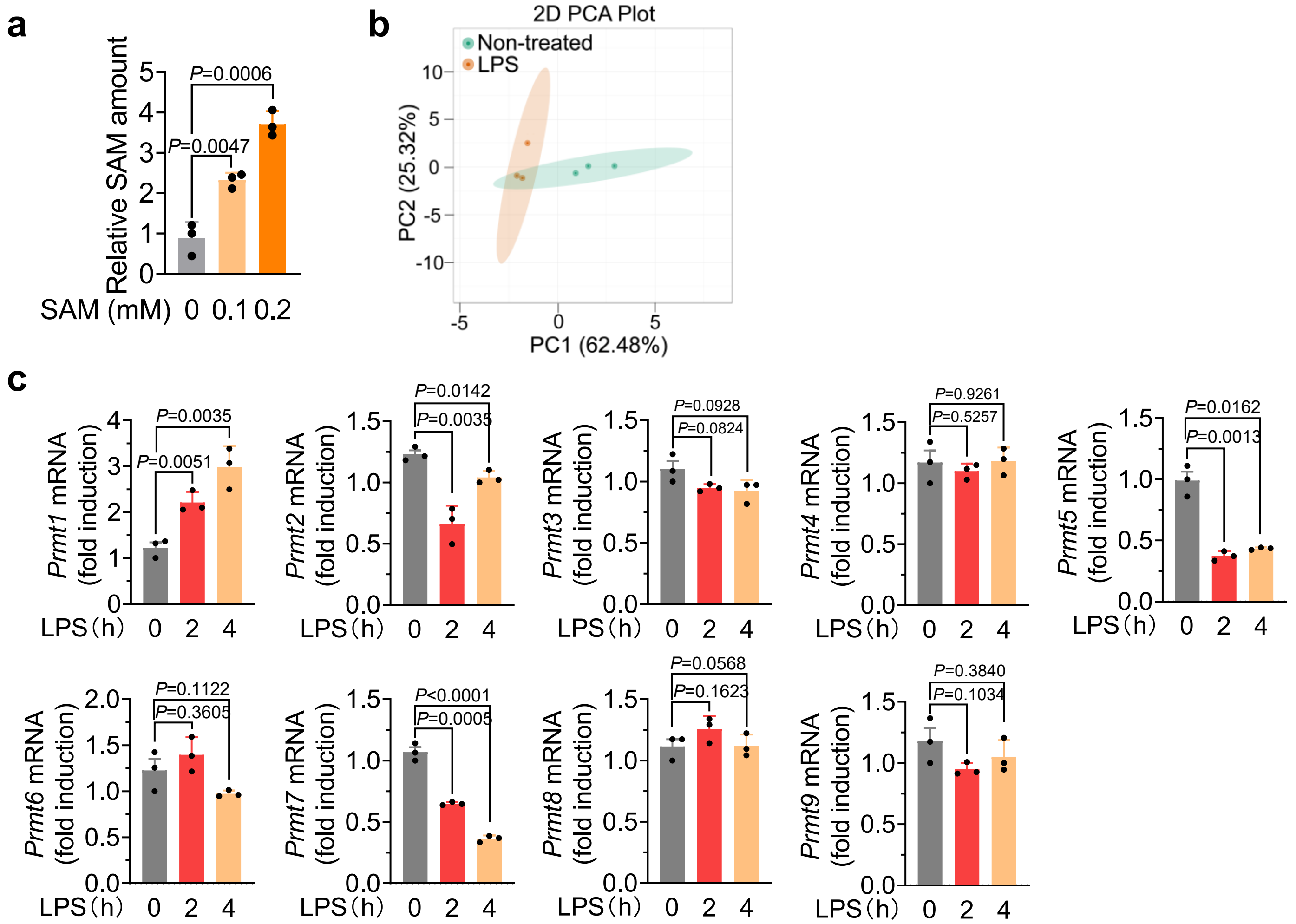

Figure S1 LPS promotes STAT3 activation depends on PRMT1.

(a) Intracellular S-adenosylmethionine (SAM) levels in bone marrow-derived macrophages (BMDMs) treated with vehicle or the indicated concentrations of SAM (100 or 200 μM) for 24 h. SAM concentrations were quantified using a commercial detection kit according to the manufacturer's protocol (n = 3). (b) Total RNA profiling by RNA sequencing was performed and analyzed using principal component analysis (PCA) on BMDMs derived from C57BL/6J mice, untreated or treated with LPS (100 ng/mL) for 2 hours (n = 3). (c) Transcripts of *Prmt1-9* in BMDMs from C57BL/6J mice stimulated with LPS for the indicated times (n = 3). Data are shown as the mean ± SD. Statistical significance in (a, c) were determined using the unpaired t-test. P<0.05 is considered statistically significant.

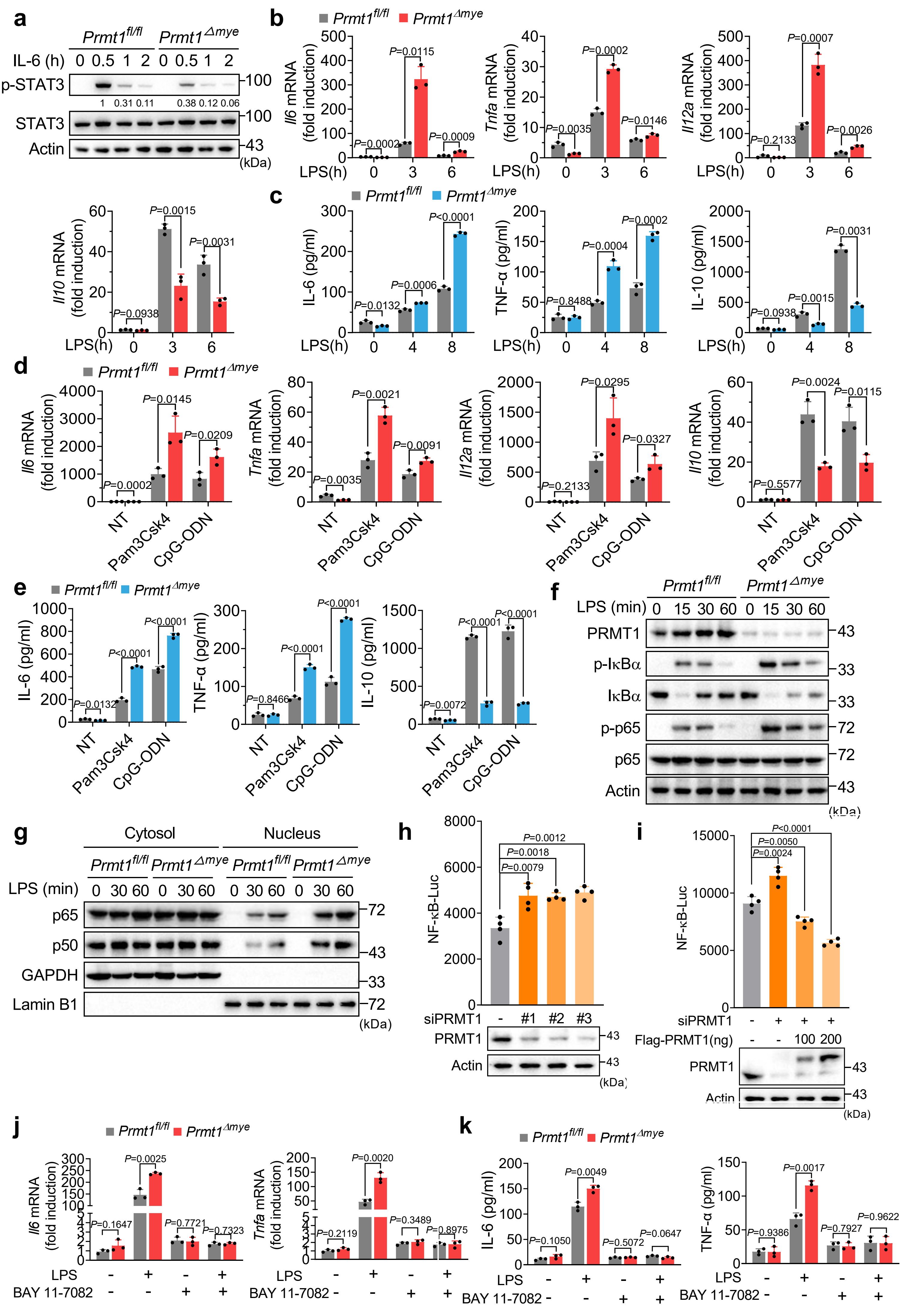

a
Prmt1fl/fl Prmt1Δmye
IL-6 (h) 0 0.5 1 2 0 0.5 1 2
p-STAT3
1 0.31 0.11 0.38 0.12 0.06
STAT3
Actin
100
100
43
(kDa)
b
Prmt1fl/fl Prmt1Δmye
Il6 mRNA (fold induction)
Tnfa mRNA (fold induction)
Il12a mRNA (fold induction)
LPS(h) 0 3 6
P=0.0002 P=0.0115 P=0.0009
P=0.0035 P=0.0002 P=0.0146
P=0.2133 P=0.0007 P=0.0026
Il10 mRNA (fold induction)
P=0.0938 P=0.0015 P=0.0031
c
Prmt1fl/fl Prmt1Δmye
IL-6 (pg/ml)
TNF-α (pg/ml)
IL-10 (pg/ml)
LPS(h) 0 4 8
P=0.0132 P=0.0006 P<0.0001
P=0.8488 P=0.0004 P=0.0002
P=0.0938 P=0.0015 P=0.0031
d
Prmt1fl/fl Prmt1Δmye
Il6 mRNA (fold induction)
P=0.0002 P=0.0145 P=0.0209
Tnfa mRNA (fold induction)
P=0.0035 P=0.0021 P=0.0091
Il12a mRNA (fold induction)
P=0.2133 P=0.0295 P=0.0327
Il10 mRNA (fold induction)
P=0.5577 P=0.0024 P=0.0115
NT Pam3Csk4 CpG-ODN
e
Prmt1fl/fl Prmt1Δmye
IL-6 (pg/ml)
P=0.0132 P<0.0001 P<0.0001
TNF-α (pg/ml)
P=0.8466 P<0.0001 P<0.0001
IL-10 (pg/ml)
P=0.0072 P<0.0001 P<0.0001
NT Pam3Csk4 CpG-ODN
f
Prmt1fl/fl Prmt1Δmye
LPS (min) 0 15 30 60 0 15 30 60
PRMT1
p-IκBα
IκBα
p-p65
p65
Actin
43 33 33 72 72 43
(kDa)
g
Cytosol Nucleus
Prmt1fl/fl Prmt1Δmye Prmt1fl/fl Prmt1Δmye
LPS (min) 0 30 60 0 30 60 0 30 60 0 30 60
p65
p50
GAPDH
Lamin B1
72 43 33 72
(kDa)
h
NF-κB-Luc
P=0.0079 P=0.0018 P=0.0012
siPRMT1 - #1 #2 #3
PRMT1
Actin
43 43
(kDa)
i
NF-κB-Luc
P=0.0024 P=0.0050 P<0.0001
siPRMT1 - + + +
Flag-PRMT1(ng) - - 100 200
PRMT1
Actin
43 43
(kDa)
j
Prmt1fl/fl Prmt1Δmye
Il6 mRNA (fold induction)
P=0.1647 P=0.0025 P=0.7721 P=0.7323
Tnfa mRNA (fold induction)
P=0.2119 P=0.0020 P=0.3489 P=0.8975
LPS - + - +
BAY 11-7082 - - + +
k
Prmt1fl/fl Prmt1Δmye
IL-6 (pg/ml)
P=0.1050 P=0.0049 P=0.5072 P=0.0647
TNF-α (pg/ml)
P=0.9386 P=0.0017 P=0.7927 P=0.9622
LPS - + - +
BAY 11-7082 - - + +

Figure S2 PRMT1 mediates STAT3 activation.

(a) Immunoblotting of phosphorylated STAT3 (Y705) and total STAT3 in *Prmt1*$^{fl/fl}$ and *Prmt1*$^{\Delta mye}$ BMDMs stimulated with IL-6 (20 ng/mL) for the indicated times. (b-e) *Prmt1*$^{fl/fl}$ and *Prmt1*$^{\Delta mye}$ BMDMs were left untreated or stimulated with LPS (100 ng/mL) (b, c) for the indicated times, Pam3CSK4 (10 μg/mL) (d) or CpG-ODN (3 mM) (e) for 3 hours. Cytokine transcripts including *Il6*, *Tnfa*, *Il12a* and *Il10* in the cells were measured with RT-qPCR (b, d), IL-6, TNF-α and IL-10 proteins in the supernatants were measured with ELISA (c, e) (n = 3). (f) Immunoblotting of PRMT1, phosphorylated IκBα (Ser32/36), IκBα, phosphorylated p65 (Ser536) and total p65 in *Prmt1*$^{fl/fl}$ and *Prmt1*$^{\Delta mye}$ BMDMs stimulated with LPS (100 ng/mL) for the indicated times. (g) Immunoblotting of p65, p50, GAPDH and Lamin B1 from cytoplasmic and nuclear fractions in *Prmt1*$^{fl/fl}$ and *Prmt1*$^{\Delta mye}$ BMDMs stimulated with LPS (100 ng/mL) for the indicated times. (h, i) NF-κB luciferase activity and immunoblotting of PRMT1 in 293T cells transfected with NF-κB luciferase reporter plasmids and either siRNAs targeting PRMT1 or Flag-tagged PRMT1 plasmids (n = 4). (j) Transcripts of *Il6* and *Tnfa* in *Prmt1*$^{fl/fl}$ and *Prmt1*$^{\Delta mye}$ BMDMs pretreated with BAY 11-7082 (10 μM) for 1 hour, followed by LPS (100 ng/mL) stimulation for 2 hours, measured by RT-qPCR (n = 3). (k) Supernatant IL-6 and TNF-α proteins levels in *Prmt1*$^{fl/fl}$ and *Prmt1*$^{\Delta mye}$ BMDMs, pretreated with BAY 11-7082 (10 μM) for 1 hours, followed by LPS (100 ng/mL) stimulation for 2 hours (n = 3). Data are shown as the mean ± SD. Statistical significance in (b-e, h-k) were determined using the unpaired t-test. The data in (a, f, g) are representative of three independent experiments. P<0.05 is considered statistically significant. NT, non- treated. Original blot can be found in Figure S14.

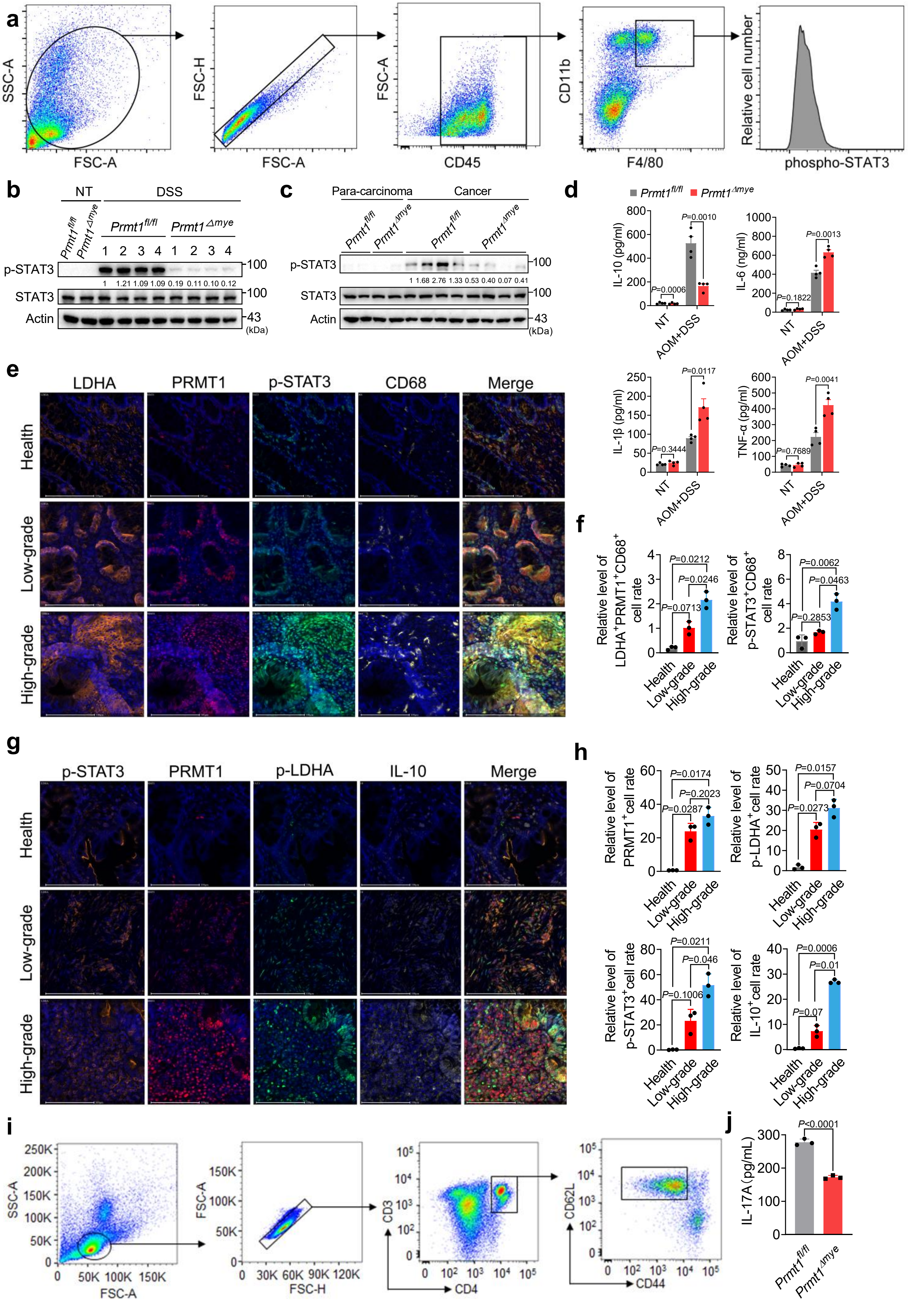

a
SSC-A
FSC-A
FSC-H
FSC-A
FSC-A
CD45
CD11b
F4/80
Relative cell number
phospho-STAT3
b
NT
DSS
Prmt1fl/fl
Prmt1Δmye
1 2 3 4 1 2 3 4
p-STAT3
STAT3
Actin
1 1.21 1.09 1.09 0.19 0.11 0.10 0.12
100
100
43
(kDa)
c
Para-carcinoma
Cancer
Prmt1fl/fl
Prmt1Δmye
p-STAT3
STAT3
Actin
1 1.68 2.76 1.33 0.53 0.40 0.07 0.41
100
100
43
(kDa)
d
Prmt1fl/fl
Prmt1Δmye
IL-10 (pg/ml)
IL-6 (ng/ml)
IL-1β (pg/ml)
TNF-α (pg/ml)
P=0.0006
P=0.0010
P=0.1822
P=0.0013
P=0.3444
P=0.0117
P=0.7689
P=0.0041
NT
AOM+DSS
e
LDHA
PRMT1
p-STAT3
CD68
Merge
Health
Low-grade
High-grade
f
Relative level of LDHA+PRMT1+CD68+ cell rate
Relative level of p-STAT3+CD68+ cell rate
P=0.0212
P=0.0246
P=0.0713
P=0.0062
P=0.0463
P=0.2853
Health
Low-grade
High-grade
g
p-STAT3
PRMT1
p-LDHA
IL-10
Merge
Health
Low-grade
High-grade
h
Relative level of PRMT1+ cell rate
Relative level of p-LDHA+ cell rate
Relative level of p-STAT3+ cell rate
Relative level of IL-10+ cell rate
P=0.0174
P=0.2023
P=0.0287
P=0.0157
P=0.0704
P=0.0273
P=0.0211
P=0.046
P=0.1006
P=0.0006
P=0.01
P=0.07
Health
Low-grade
High-grade
i
SSC-A
FSC-A
FSC-A
FSC-H
CD3
CD4
CD62L
CD44
j
IL-17A (pg/mL)
P<0.0001
Prmt1fl/fl
Prmt1Δmye

Figure S3 *Prmt1$^{\Delta mye}$* mice are more susceptible to experimental colitis and colitis-associated colorectal cancer. (a) The gating strategy for detecting phosphorylated STAT3 levels in colon macrophages ($CD11b^+F4/80^+$) was performed using FACS analysis. (b, c) Immunoblotting of phosphorylated STAT3 (Y705) and total STAT3 in colon tissues from NT or DSS-treated *Prmt1$^{fl/fl}$* and *Prmt1$^{\Delta mye}$* mice (b), and colon tissues from para-carcinoma and cancer lesions of AOM+DSS-treated *Prmt1$^{fl/fl}$* and *Prmt1$^{\Delta mye}$* mice (c). (d) IL-10, IL-6, IL-1β and TNF-α in colon explant cultures were measured by ELISA (n = 4). (e) Representative confocal images of tyramide signal amplification-amplified four-plex staining for CD68 (yellow), PRMT1 (red), LDHA (orange), and phospho-STAT3 (Y705) (green) in FFPE colon mucosa from active IBD, remission, and non-IBD controls. Scale bar = 100 μm. (f) Relative level of positive cell rate from active IBD, remission, and non-IBD controls (n = 3). (g) Representative confocal images of tyramide signal amplification-amplified four-plex staining for PRMT1 (red), phospho-LDHA (Y10) (green), phospho-STAT3 (Y705) (orange), and IL-10 (yellow) in FFPE colon mucosa from active IBD, remission, and non-IBD controls. Scale bar = 100 μm. (h) Relative level of positive cell rate from active IBD, remission, and non-IBD controls (n = 3). (i) Representative flow cytometry gating strategy for isolation of naïve $CD4^+$ T cells ($CD4^+$ $CD44^−$ $CD62L^+$) from spleens of *Prmt1$^{fl/fl}$* and *Prmt1$^{\Delta mye}$* mice prior to *in vitro* Th17 polarization. (j) Naïve $CD4^+$ T cells isolated as in (i) were cultured under standard Th17-polarizing conditions (TGF-β, IL-6, anti-IFN-γ, anti-IL-4). IL-17A concentrations in culture supernatants after 5 days were quantified by ELISA (n = 3). Data are shown as the mean ± SD. Statistical significance in (d, j) were determined using the *t*-test (and nonparametric tests). Statistical significance in (f, h) were determined using the one-way ANOVA. The data in (b, c) are representative of three independent experiments. P<0.05 is considered statistically significant. NT, untreated. Original blot can be found in Figure S15.

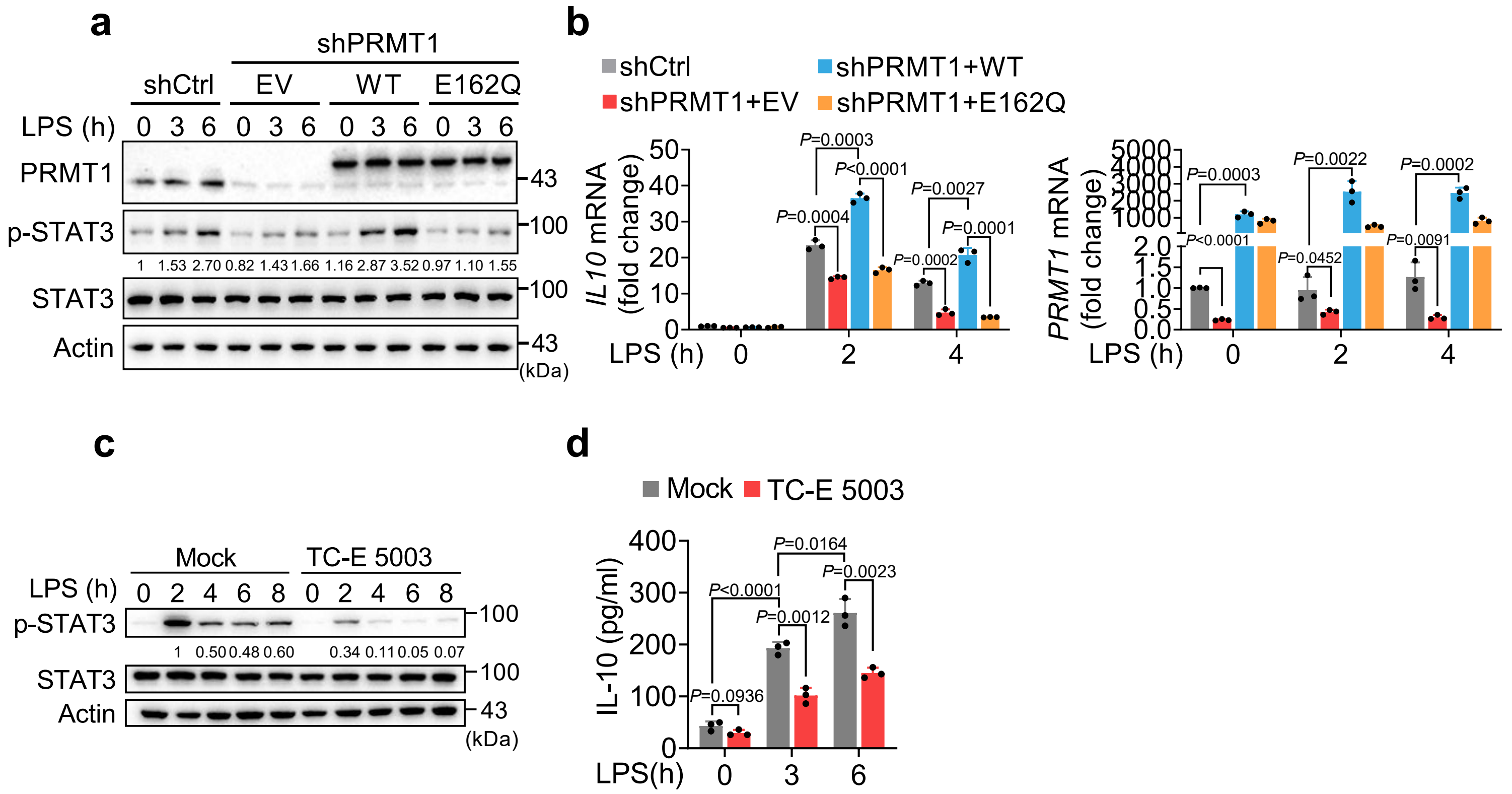

Figure S4 PRMT1 promotes STAT3 activation and IL-10 production through its enzymatic activity.

(a, b) Immunoblotting of PRMT1, phosphorylated STAT3 (Y705) and total STAT3 (a), transcriptional levels of *IL10* and *PRMT1* (b) from shCtrl or shPRMT1 THP-1 cells reconstituted with Flag-tagged wild type or E162Q PRMT1 plasmids, followed by LPS (100 ng/mL) stimulation for the indicated times (n = 3). (c) Immunoblotting of phosphorylated STAT3 (Y705) and total STAT3 in BMDMs, left untreated or stimulated with TC-E 5003 (2 μM) for 12 hours, followed by LPS (100 ng/mL) challenge for the indicated times. (d) Supernatant IL-10 protein levels in BMDMs, left untreated or stimulated with TC-E 5003 (2 μM) for 12 hours, followed by LPS (100 ng/mL) challenge for the indicated times (n = 3). Data are shown as the mean ± SD. Statistical significance in (b, d) were determined using the unpaired t-test. The data in (a, c) are representative of three independent experiments. P<0.05 is considered statistically significant. Original blot can be found in Figure S16.

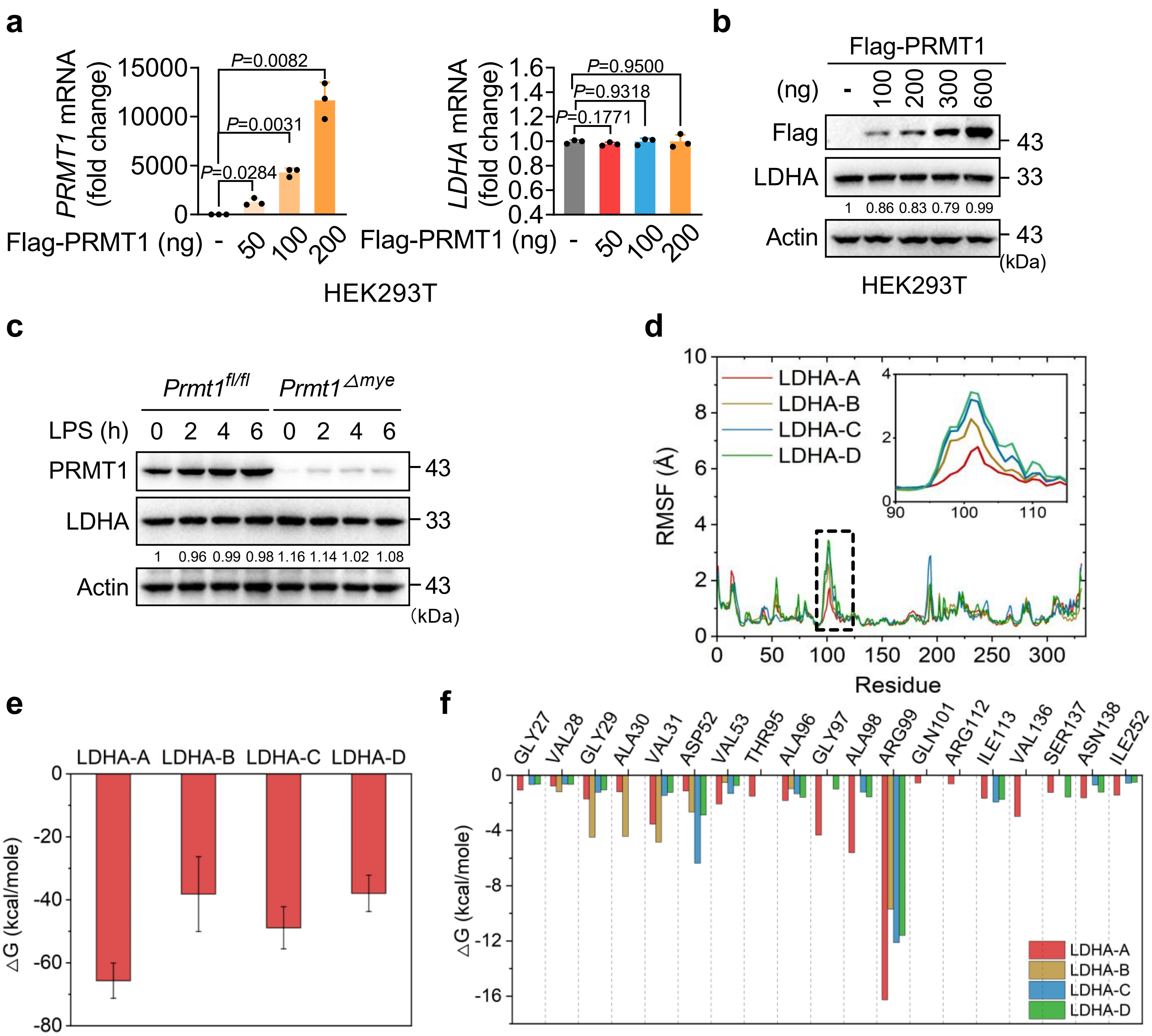

Figure S5 PRMT1 interacts with LDHA and regulates STAT3 activation.

(a) The transcripts of *PRMT1* and *LDHA* in HEK293T cells transfected with different amounts of PRMT1 expression plasmids (n = 3). (b) Immunoblotting of LDHA in HEK293T cells transfected with different amounts of PRMT1 expression plasmids. (c) Western blot analysis of total LDHA protein levels in BMDMs from *Prmt1*$^{fl/fl}$ and *Prmt1*$^{\Delta mye}$ mice. Actin was used as loading control. (d) The RMSF curves of the LDHA tetramer structure from MD simulation with an inset figure that magnifies the dashed square region, providing a close-up view of the fluctuations around the active site loop (residues 90-115). (e) The NAD+ substrate binding energy data from three repeats for the PRMT1-LDHA tetramer system indicate that the LDHA-A chain, which interacts with PRMT1, exhibits significantly higher binding energy compared to the other three chains bound to PRMT1. (f) Energy contribution analysis of residues across different chains in the LDHA tetramer structure. Data are shown as the mean ± SD. Statistical significance in (a) was determined using the unpaired t-test. The data in (b, c) are representative of three independent experiments. P<0.05 is considered statistically significant. Original blot can be found in Figure S17.

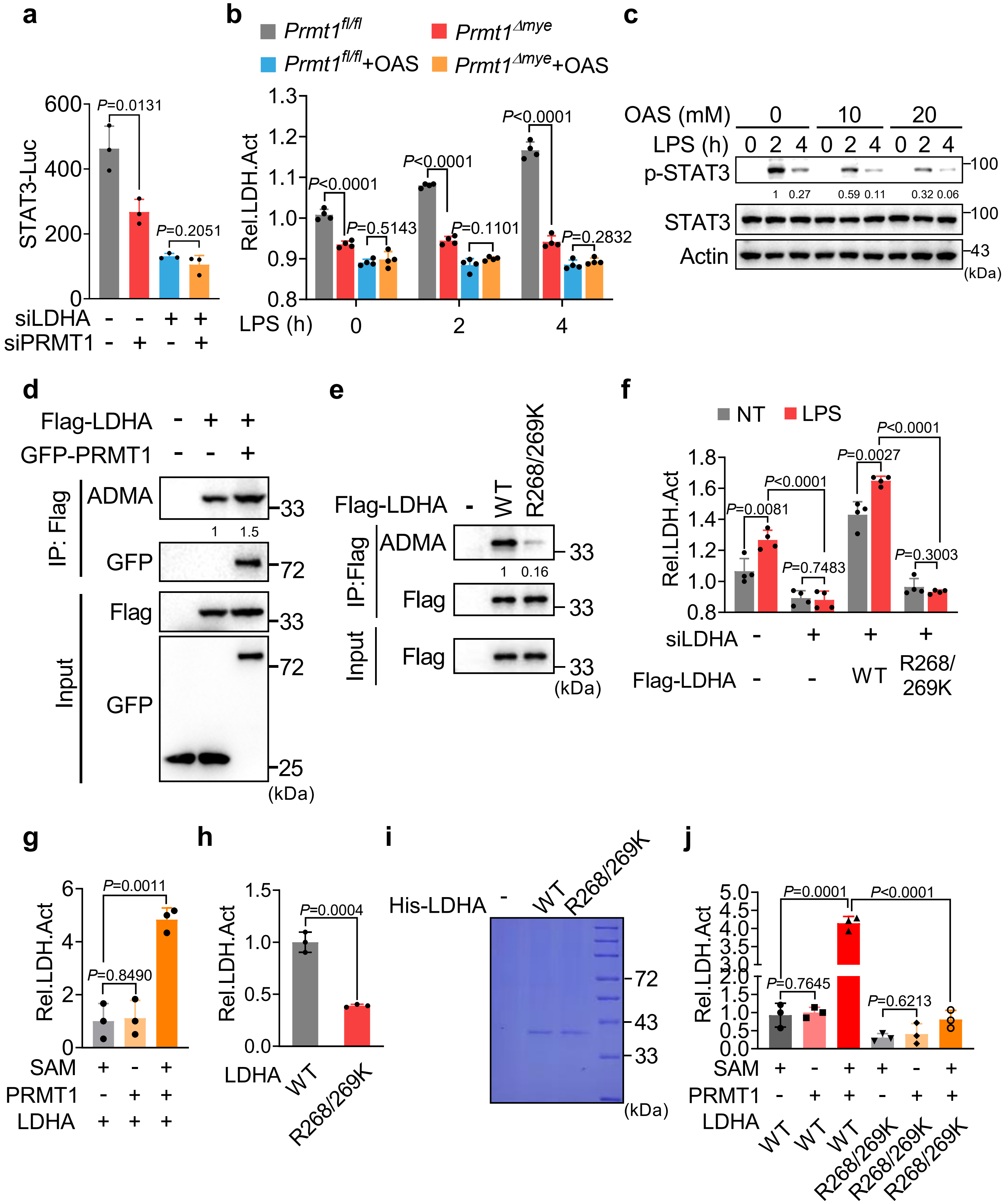

a
STAT3-Luc
P=0.0131
P=0.2051
siLDHA - - + +
siPRMT1 - + - +
b
Prmt1fl/fl
Prmt1Δmye
Prmt1fl/fl+OAS
Prmt1Δmye+OAS
Rel.LDH.Act
P<0.0001
P=0.5143
P<0.0001
P=0.1101
P<0.0001
P=0.2832
LPS (h) 0 2 4
c
OAS (mM) 0 10 20
LPS (h) 0 2 4 0 2 4 0 2 4
p-STAT3
1 0.27 0.59 0.11 0.32 0.06
STAT3
Actin
100
100
43
(kDa)
d
Flag-LDHA - + +
GFP-PRMT1 - - +
IP: Flag
ADMA
1 1.5
GFP
Input
Flag
GFP
33
72
33
72
25
(kDa)
e
Flag-LDHA - WT R268/269K
IP:Flag
ADMA
1 0.16
Flag
Input
Flag
33
33
33
(kDa)
f
NT
LPS
Rel.LDH.Act
P=0.0081
P<0.0001
P=0.0027
P<0.0001
P=0.7483
P=0.3003
siLDHA - + + +
Flag-LDHA - - WT R268/269K
g
Rel.LDH.Act
P=0.0011
P=0.8490
SAM + - +
PRMT1 - + +
LDHA + + +
h
Rel.LDH.Act
P=0.0004
LDHA WT R268/269K
i
His-LDHA - WT R268/269K
72
43
33
(kDa)
j
Rel.LDH.Act
P=0.0001
P<0.0001
P=0.7645
P=0.6213
SAM + - + + - +
PRMT1 - + + - + +
LDHA WT WT WT R268/269K R268/269K R268/269K

Figure S6 LDHA activity is critical for PRMT1-mediated STAT3 activation.

(a) STAT3 luciferase activity in THP-1 cells transfected with control siRNA (siCtrl), siRNA targeting LDHA (siLDHA), siRNA targeting PRMT1 (siPRMT1), or a combination of siLDHA and siPRMT1 (n = 3). (b) Intracellular LDHA activity in *Prmt1*$^{fl/fl}$ and *Prmt1*$^{\Delta mye}$ BMDMs left untreated or pretreated with OAS (10 mM) for 16 hours, followed by LPS (100 ng/ml) stimulation for indicated times (n = 4). (c) Immunoblotting of phosphorylated STAT3 (Y705) and total STAT3 in BMDMs pretreated with the indicated concentrations of OAS, followed by LPS stimulation for the specified durations. (d) 293T cells were transfected with the indicated plasmids for 30 hours, and immunoprecipitated proteins were analyzed using the specified antibodies. (e) Asymmetric dimethylarginine (ADMA) signal on wild-type, and R268/269K mutant LDHA proteins expressed in HEK293T cells. ADMA was detected by anti-ADMA antibody following immunoprecipitation. (f) Intracellular LDH activity in THP-1 cells transfected with control siRNA (siCtrl), siRNA targeting LDHA (siLDHA), or siLDHA in combination with LDHA WT or LDHA R268/269K mutant expression plasmids, followed by either untreated or LPS stimulation for 2 hours (n = 4). (g) *In vitro* methylation and His-LDHA activity was determined by measuring the rate of NADH oxidation in a reaction mixture containing 20 mM HEPES (pH 7.2), 20 μM NADH, 0.05% bovine serum albumin, and 2 mM pyruvate. Fluorescence was recorded using a microplate reader at excitation/emission wavelengths of 340/460 nm (n = 3). (h) LDHA activity was quantified by monitoring NADH oxidation in a reaction mixture containing LDHA WT or mutant protein, HEPES (20 mM , pH 7.2), NADH (20 μM), 0.05% bovine serum albumin, and pyruvate (2 mM). Fluorescence was recorded using a microplate reader at excitation/emission wavelengths of 340/460 nm (n = 3). (i) SDS-PAGE analysis of purified LDHA-WT and LDHA-R268/269K proteins. (j) Recombinant WT and R268/269K LDHA were each tested under three parallel conditions: one with PRMT1 and SAM (methylated), a second without PRMT1, and a third without SAM (both mock-treated). After re-purification, LDHA activity was measured (n = 3). Data are shown as the mean ± SD. Statistical significance in (a, b, f, g) was determined using the unpaired t-test. The data in (c, d, e, j) are representative of three independent experiments. $P<0.05$ is considered statistically significant. Original blot can be found in Figure S18.

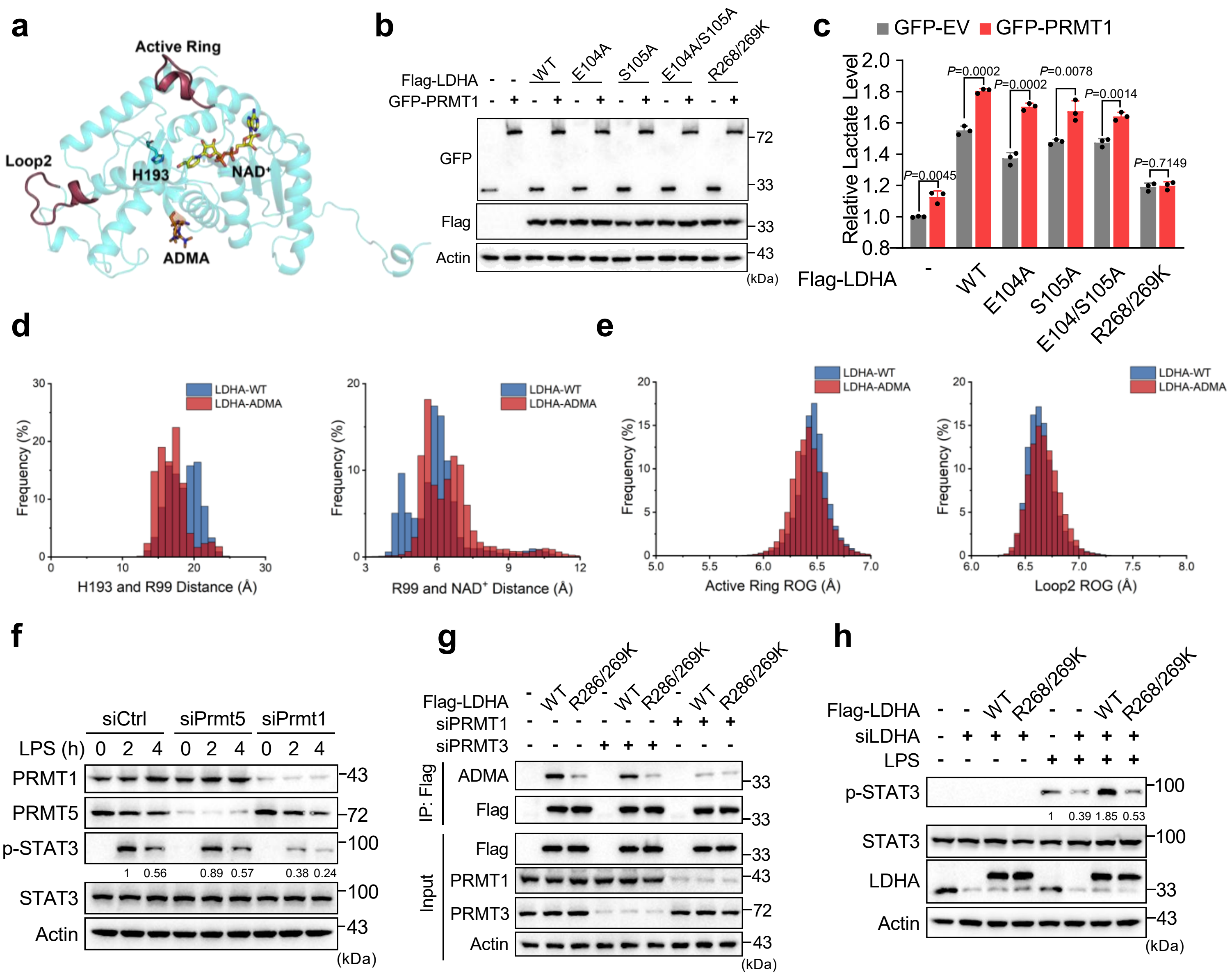
a
Active Ring
Loop2
H193
NAD+
ADMA
b
Flag-LDHA
GFP-PRMT1
WT
E104A
S105A
E104A/S105A
R268/269K
GFP
Flag
Actin
72
33
33
43
(kDa)
c
GFP-EV
GFP-PRMT1
Relative Lactate Level
P=0.0045
P=0.0002
P=0.0002
P=0.0078
P=0.0014
P=0.7149
Flag-LDHA
WT
E104A
S105A
E104/S105A
R268/269K
d
LDHA-WT
LDHA-ADMA
Frequency (%)
H193 and R99 Distance (Å)
R99 and NAD+ Distance (Å)
e
Active Ring ROG (Å)
Loop2 ROG (Å)
f
siCtrl
siPrmt5
siPrmt1
LPS (h)
PRMT1
PRMT5
p-STAT3
1
0.56
0.89
0.57
0.38
0.24
STAT3
Actin
(kDa)
g
Flag-LDHA
WT
R286/269K
siPRMT1
siPRMT3
IP: Flag
ADMA
Flag
Input
Flag
PRMT1
PRMT3
Actin
(kDa)
h
Flag-LDHA
WT
R268/269K
siLDHA
LPS
p-STAT3
1
0.39
1.85
0.53
STAT3
LDHA
Actin
(kDa)

Figure S7 Structural and conformational analyses of flexible loop regions upon ADMA modification of LDHA. (a) Structural localization of flexible regions in LDHA upon ADMA modification. The Active Ring (residues 98–110) and Loop2 (residues 220–230) identified from molecular dynamics simulations are highlighted in red. (b) Immunoblotting of Flag-tagged LDHA constructs (WT, E104A, S105A, E104A/S105A, R268K/R269K) in HEK293T cells co-transfected with empty vector or wild-type PRMT1. (c) Relative lactate levels in cell lysates from the experiments in (B) (n = 3). (d) Distance frequency measurements between key catalytic residues in WT and ADMA-modified LDHA systems. Left: the distances between R99 with H193. Right: the distances between R99 with the NAD+. (e) Radius of gyration analysis of flexible loop regions. Left: The Active Ring. Right: Loop2. (f) Immunoblot analysis of PRMT5, PRMT1, phospho-STAT3 (Y705), total STAT3, and β-actin in peritoneal macrophages transfected with scrambled siRNA, *Prmt5* siRNA, or *Prmt1* siRNA, followed by LPS stimulation (100 ng/mL, 2 h). (g) Immunoblot analysis of asymmetric dimethylation of overexpressed LDHA (upper panel) and input controls (lower panel) in HEK293T cells transfected with scrambled, *PRMT3*, or *PRMT1* siRNA, followed by overexpression of Flag-tagged empty vector (EV), wild-type (WT) LDHA, or R268K/R269K mutant LDHA. Flag-tagged LDHA was immunoprecipitated with anti-Flag antibody and blotted with anti-ADMA antibody (upper panel); total Flag-LDHA in immunoprecipitate shown. Input lysates were blotted for PRMT3, PRMT1, Flag, and β-actin (lower panel). (h) Immunoblotting of phosphorylated STAT3 (Y705), total STAT3, and LDHA in THP-1 cells transfected with control siRNA (siCtrl), siRNA targeting LDHA (siLDHA), or siLDHA in combination with LDHA WT or LDHA R268/269K mutant expression plasmids, followed by either untreated or LPS stimulation for 2 hours. Data are shown as the mean ± SD. Statistical significance in (c) was determined using the unpaired t-test. The data in (f-h) are representative of three independent experiments. $P<0.05$ is considered statistically significant. Original blot can be found in Figure S19.

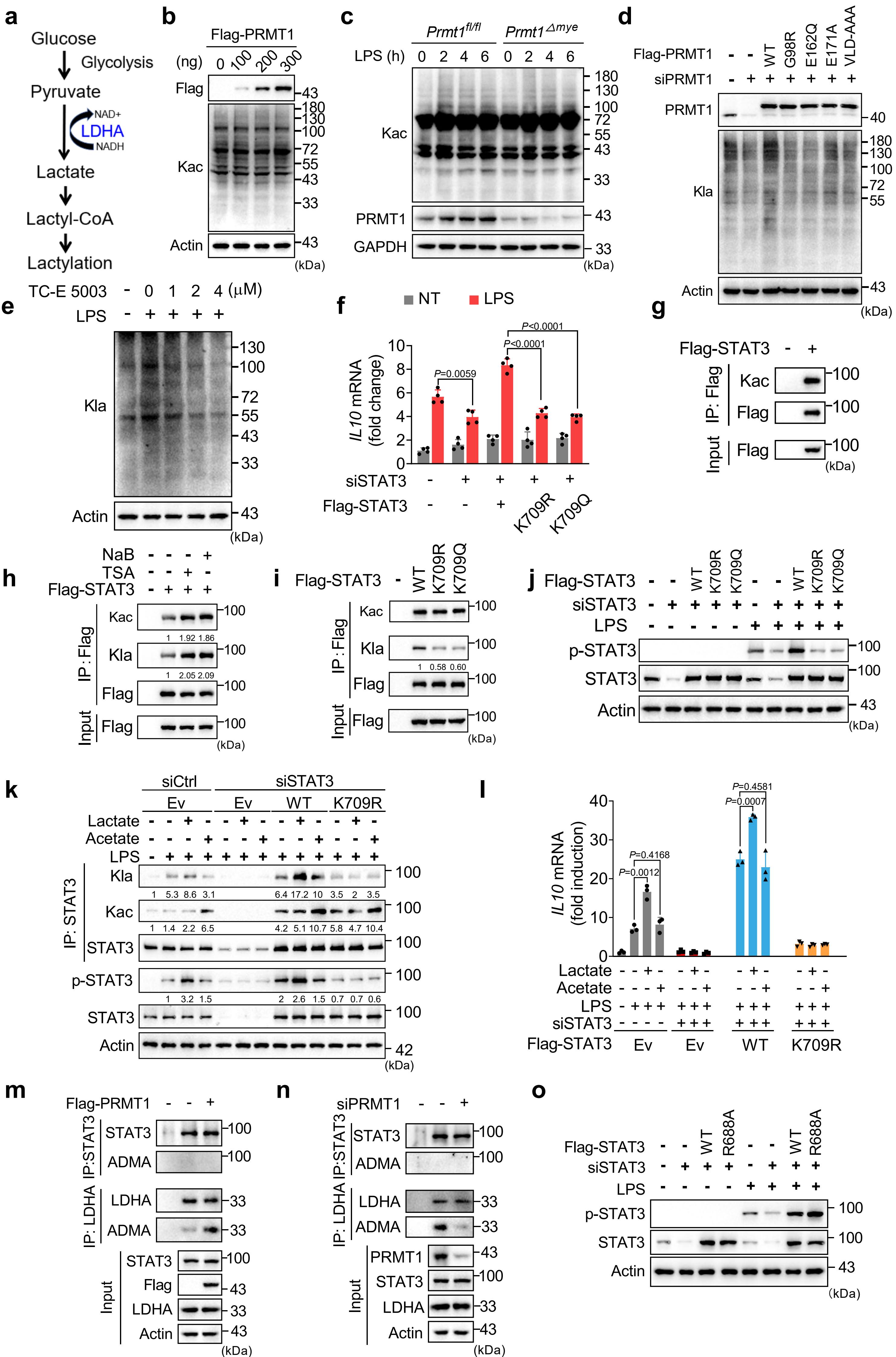
a
Glucose
Glycolysis
Pyruvate
NAD+
LDHA
NADH
Lactate
Lactyl-CoA
Lactylation
b
Flag-PRMT1
(ng) 0 100 200 300
Flag
Kac
Actin
43
180
130
100
72
55
43
33
43
(kDa)
c
Prmt1fl/fl
Prmt1Δmye
LPS (h) 0 2 4 6 0 2 4 6
Kac
PRMT1
GAPDH
180
130
100
72
55
43
33
43
33
(kDa)
d
Flag-PRMT1 - - WT G98R E162Q E171A VLD-AAA
siPRMT1 - + + + + + +
PRMT1
Kla
Actin
40
180
130
100
72
55
43
(kDa)
e
TC-E 5003 - 0 1 2 4 (μM)
LPS - + + + +
Kla
Actin
130
100
72
55
43
33
43
(kDa)
f
NT
LPS
IL10 mRNA (fold change)
P=0.0059
P<0.0001
P<0.0001
siSTAT3 - + + + +
Flag-STAT3 - - + K709R K709Q
g
Flag-STAT3 - +
IP: Flag
Kac
Flag
Input
Flag
100
100
100
(kDa)
h
NaB - - - +
TSA - - + -
Flag-STAT3 - + + +
IP : Flag
Kac
1 1.92 1.86
Kla
1 2.05 2.09
Flag
Input
Flag
100
(kDa)
i
Flag-STAT3 - WT K709R K709Q
IP : Flag
Kac
Kla
1 0.58 0.60
Flag
Input
Flag
100
(kDa)
j
Flag-STAT3 - - WT K709R K709Q - - WT K709R K709Q
siSTAT3 - + + + + - + + + +
LPS - - - - - + + + + +
p-STAT3
STAT3
Actin
100
100
43
(kDa)
k
siCtrl
siSTAT3
Ev
Ev
WT
K709R
Lactate
Acetate
LPS
IP: STAT3
Kla
1 5.3 8.6 3.1 6.4 17.2 10 3.5 2 3.5
Kac
1 1.4 2.2 6.5 4.2 5.1 10.7 5.8 4.7 10.4
STAT3
p-STAT3
1 3.2 1.5 2 2.6 1.5 0.7 0.7 0.6
STAT3
Actin
100
42
(kDa)
l
IL10 mRNA (fold induction)
P=0.4581
P=0.0007
P=0.4168
P=0.0012
Lactate - - + - - + - - + - - + -
Acetate - - - + - - + - - + - - +
LPS - + + + + + + + + + + + +
siSTAT3 - - - - + + + + + + + + +
Flag-STAT3 Ev Ev WT K709R
m
Flag-PRMT1 - - +
IP:STAT3
STAT3
ADMA
IP: LDHA
LDHA
ADMA
Input
STAT3
Flag
LDHA
Actin
100
33
43
(kDa)
n
siPRMT1 - - +
IP:STAT3
STAT3
ADMA
IP: LDHA
LDHA
ADMA
Input
PRMT1
STAT3
LDHA
Actin
100
33
43
(kDa)
o
Flag-STAT3 - - WT R688A - - WT R688A
siSTAT3 - + + + - + + +
LPS - - - - + + + +
p-STAT3
STAT3
Actin
100
43
(kDa)

Figure S8 Lactylation of STAT3 on K709 is critical for its activation.

(a) Schematic diagram of lactate metabolism. (b, c) Immunoblotting for total acetylation in HEK293T cells transfected with increasing amounts of Flag-tagged PRMT1 expression plasmid (b), or in *Prmt1*$^{fl/fl}$ and *Prmt1*$^{\Delta mye}$ BMDMs left untreated or stimulated with LPS (100 ng/mL) for the indicated times (c). (d, e) Immunoblotting of total lactylation in PRMT1-knockdown 293T cells reconstituted with Flag-tagged PRMT1 wild type and enzymatic-dead mutant expression plasmids (d), or in BMDMs left untreated or treated with TC-E 5003 (2 μM) and stimulated with LPS (100 ng/ml) for 2 hours (e). (f) Quantification of *IL-10* mRNA levels by qRT-PCR in STAT3-knockdown THP-1 cells reconstituted with wild-type (WT) STAT3 or the lactylation-deficient K709 mutant, following stimulation with LPS (100 ng/mL) for indicated times (n = 4). (g, h) Acetylation of overexpressed STAT3 in HEK293T cells under basal conditions (g) or following treatment with sodium butyrate (1 mM) or TSA (100 nM) (h). (i) Immunoprecipitation of Flag-tagged wild-type, K709R, or K709Q STAT3 followed by immunoblotting for lactylation, acetylation and Flag. (j) Reconstitution of STAT3-knockout THP-1 cells with wild-type, K709R, or K709Q STAT3, followed by immunoblotting for p-STAT3 (Y705) and total STAT3 upon stimulation. (k, l) Immunoprecipitation of STAT3 and immunoblotting of Kla, Kac, p-STAT3 (Y705), quantification of IL10 mRNA levels by qRT-PCR in STAT3-knockdown THP-1 cells reconstituted with wild-type (WT) STAT3 or the lactylation-deficient K709 mutant, pretreated with lactate (20 mM) or acetate (20 mM) for 6 hours, followed by LPS (100 ng/mL) stimulation for 2 hours (n = 3). (m, n) Immunoprecipitation of STAT3 from THP-1 cells overexpressing PRMT1 (m) or with PRMT1 knockdown (n), followed by immunoblotting with pan-asymmetric dimethylarginine (ADMA) antibody. Parallel samples immunoblotted for ADMA modification of LDHA serve as positive control. (o) THP-1 cells were transfected with control or STAT3-specific siRNA, followed by reconstitution with vector, wild-type (WT) STAT3, or R688A mutant STAT3. Cells were left unstimulated (NT) or stimulated with LPS for 2 hours, and lysates were immunoblotted for phosphorylated STAT3 (Y705), total STAT3, and Actin. Data are shown as the mean ± SD. Statistical significance in (f, l) was determined using the unpaired t-test. The data in (b-e, g-k) are representative of three independent experiments. P<0.05 is considered statistically significant. Original blot can be found in Figure S20.

Figure 1e

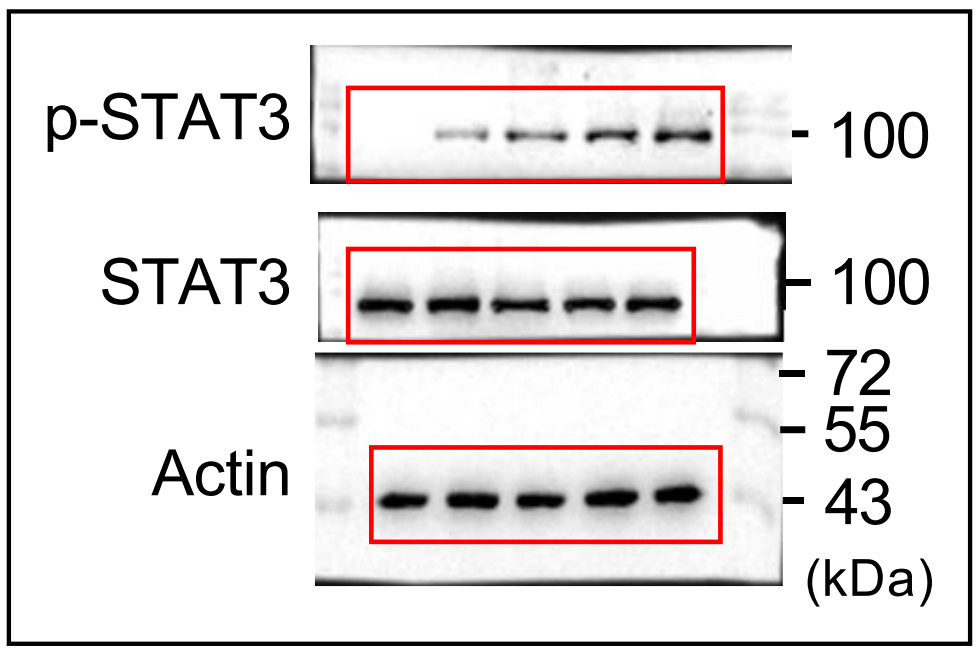

Figure 1i

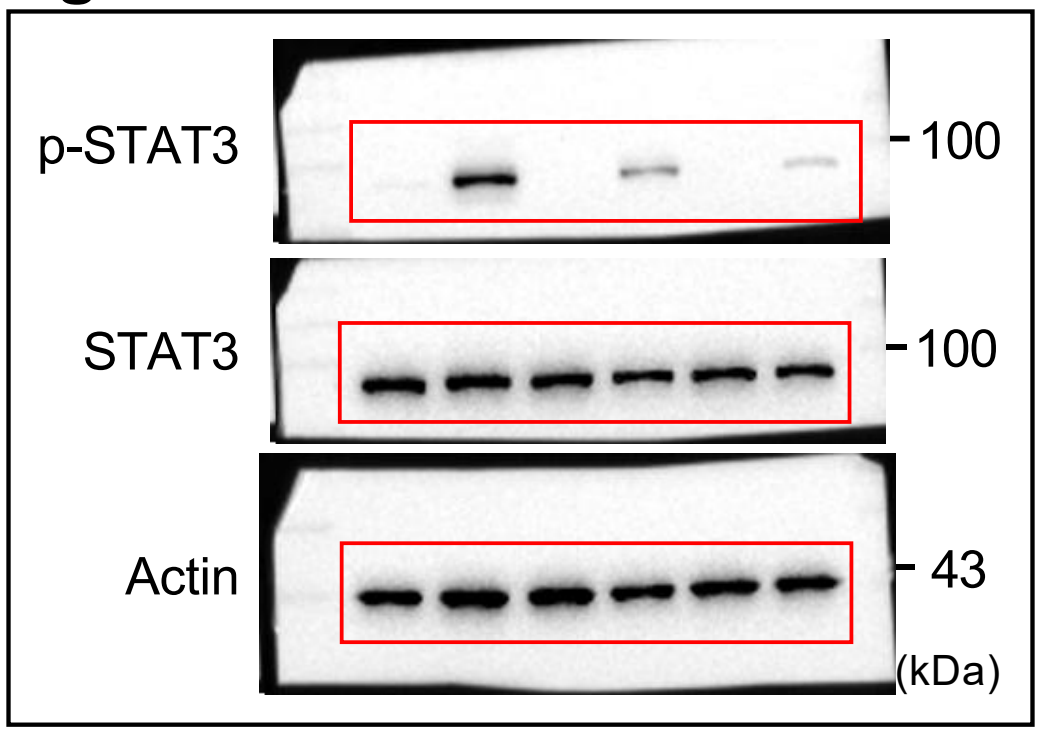

Figure 1k

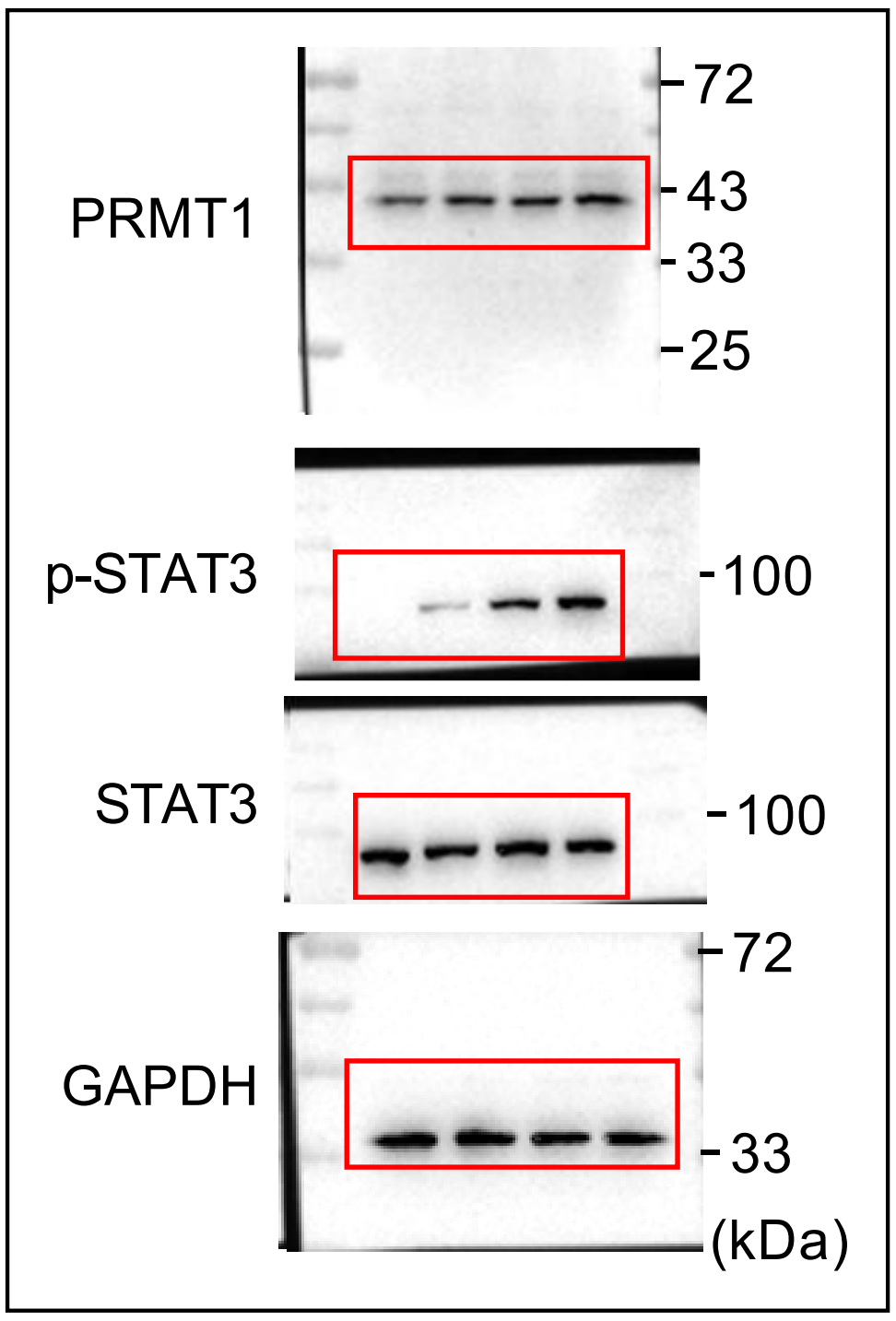

Figure 1m

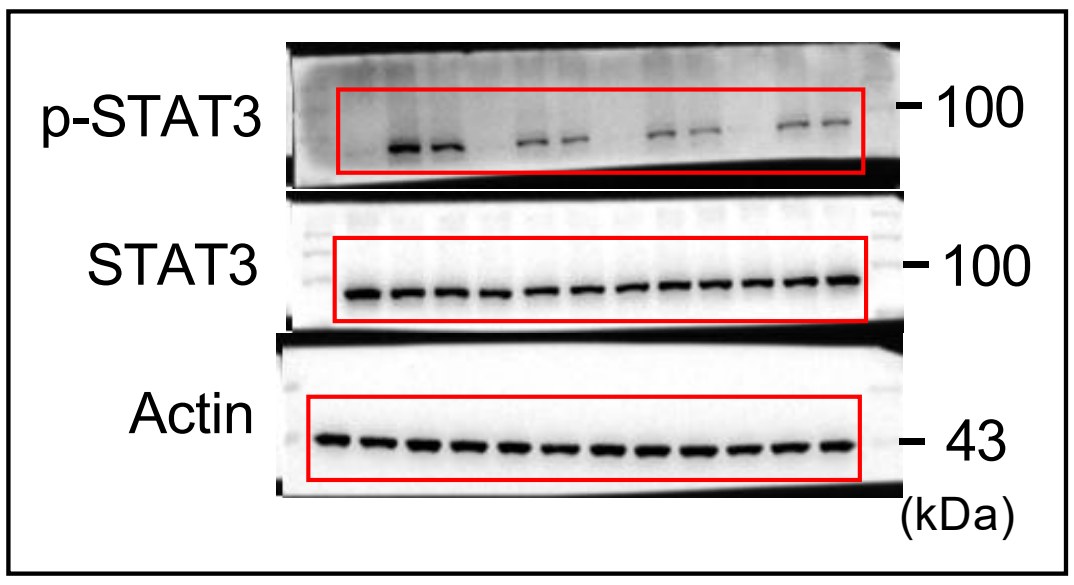

Figure 1n

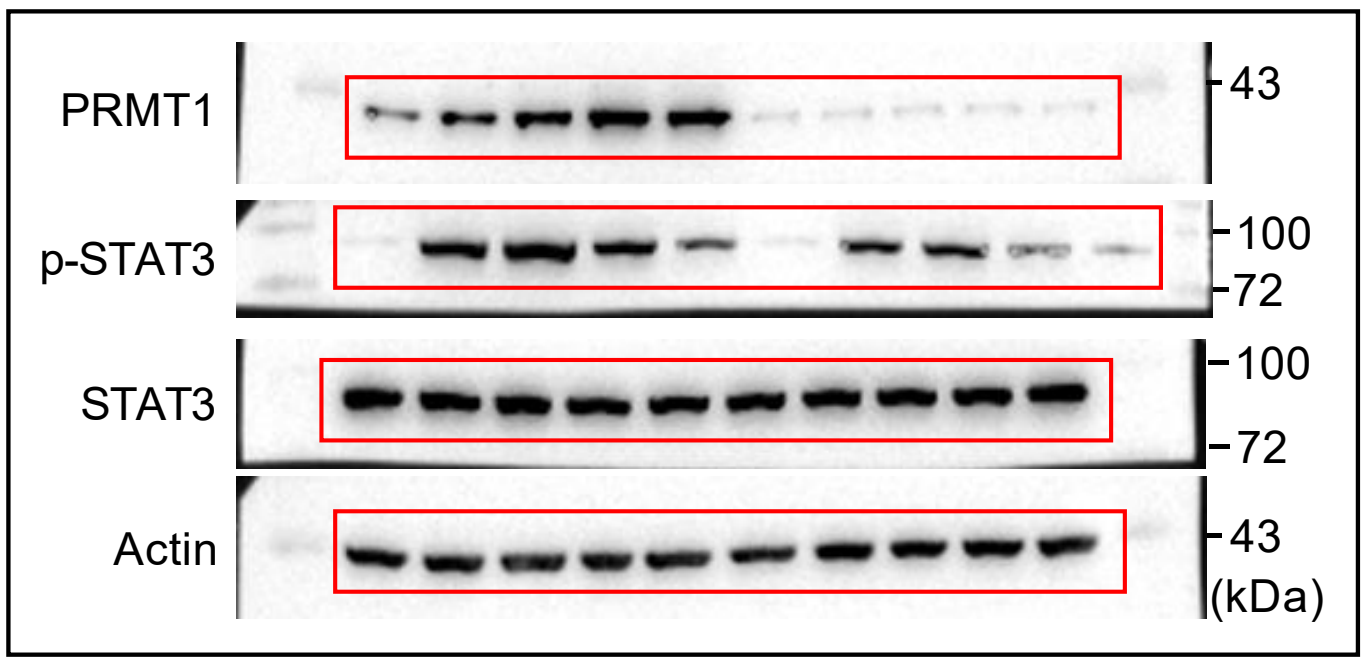

Figure 1o

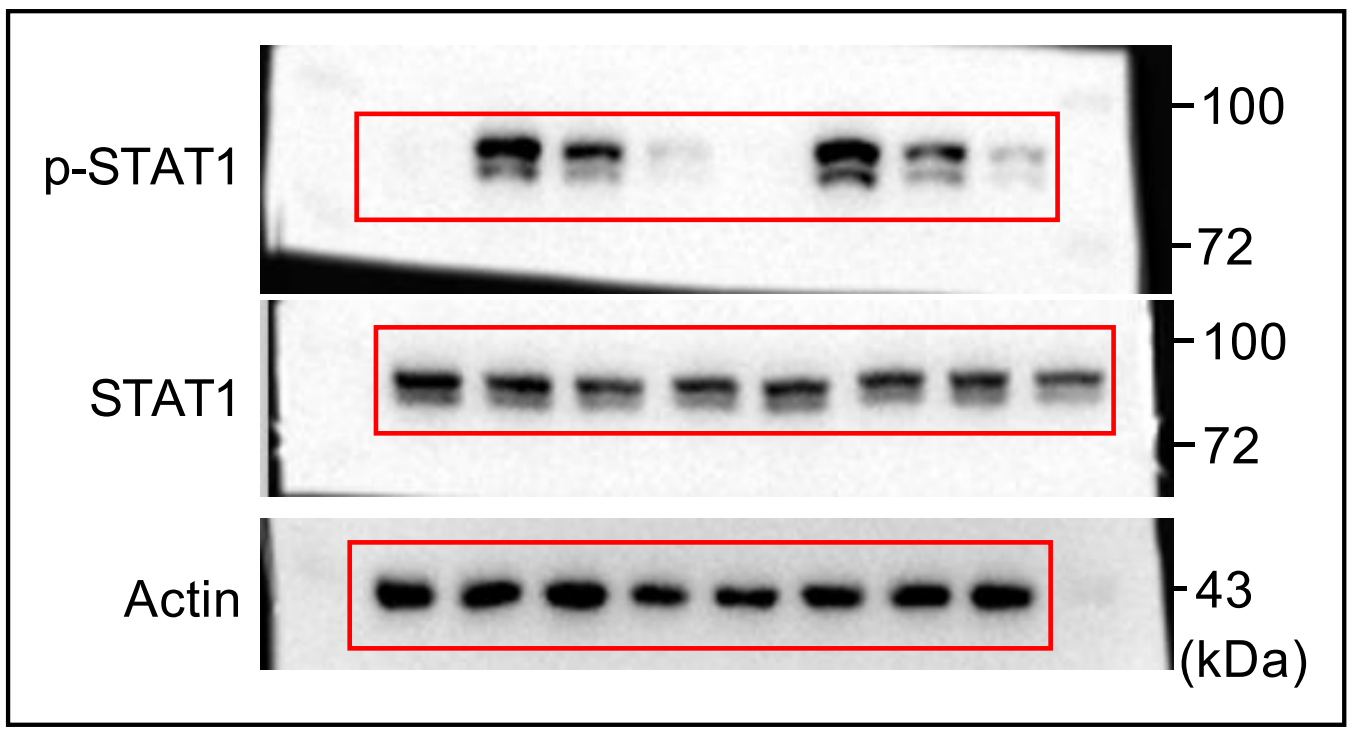

Figure 1p

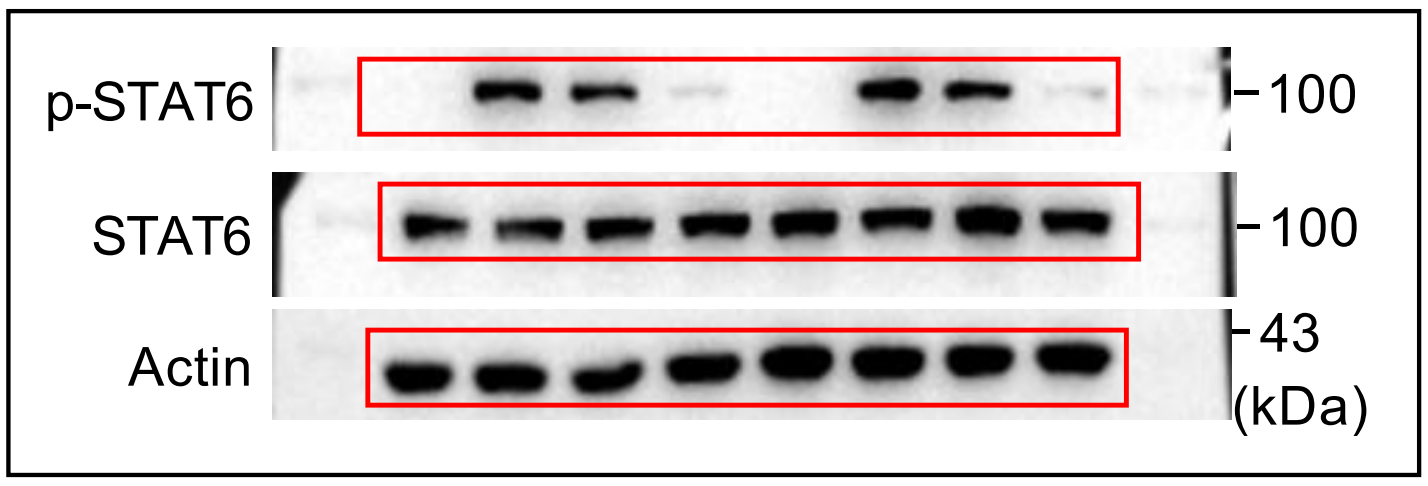

Figure S9 (Related to Figure 1)

Figure 3b

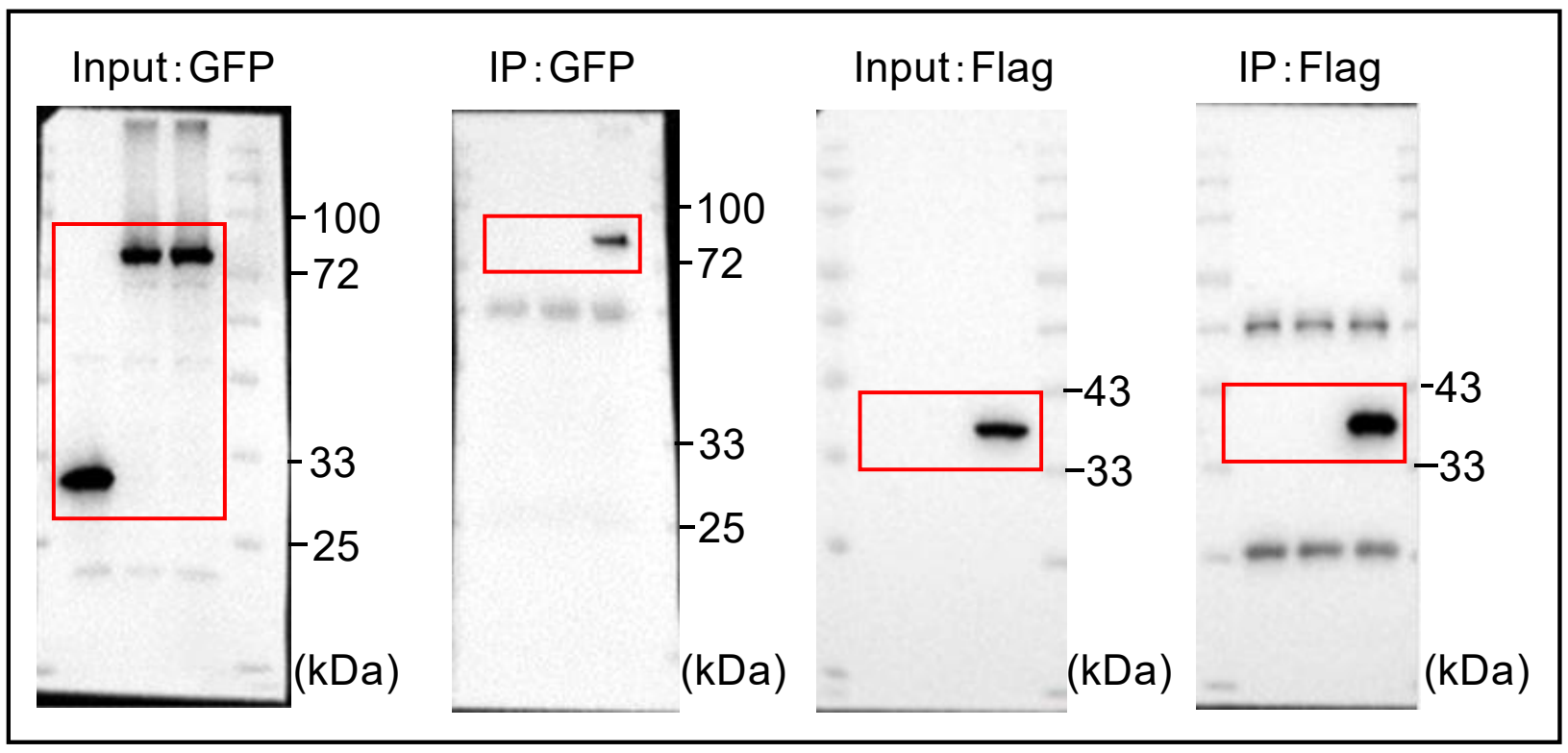

Figure 3g

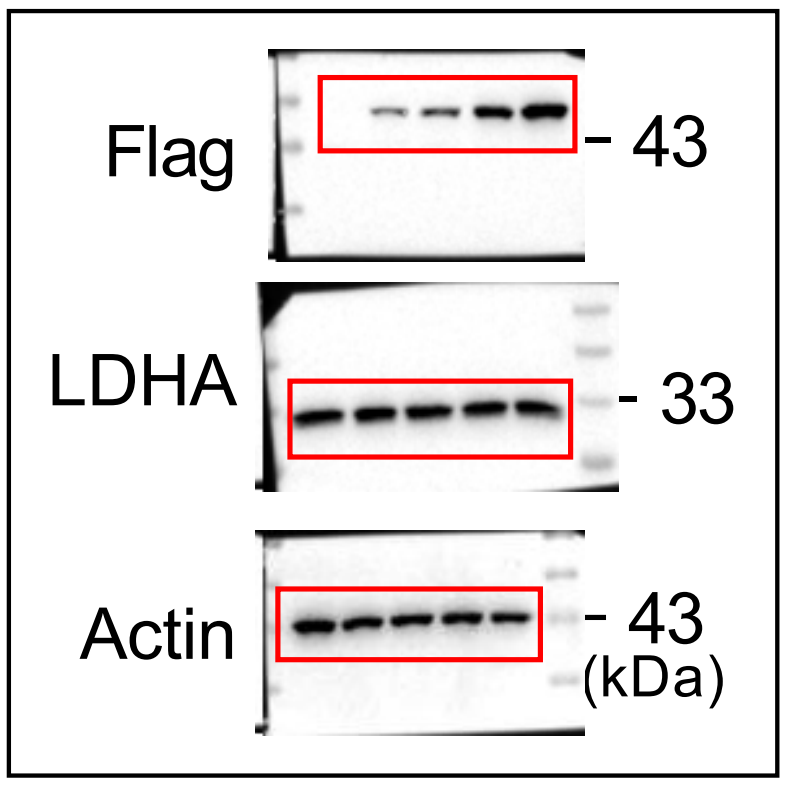

Figure 3h

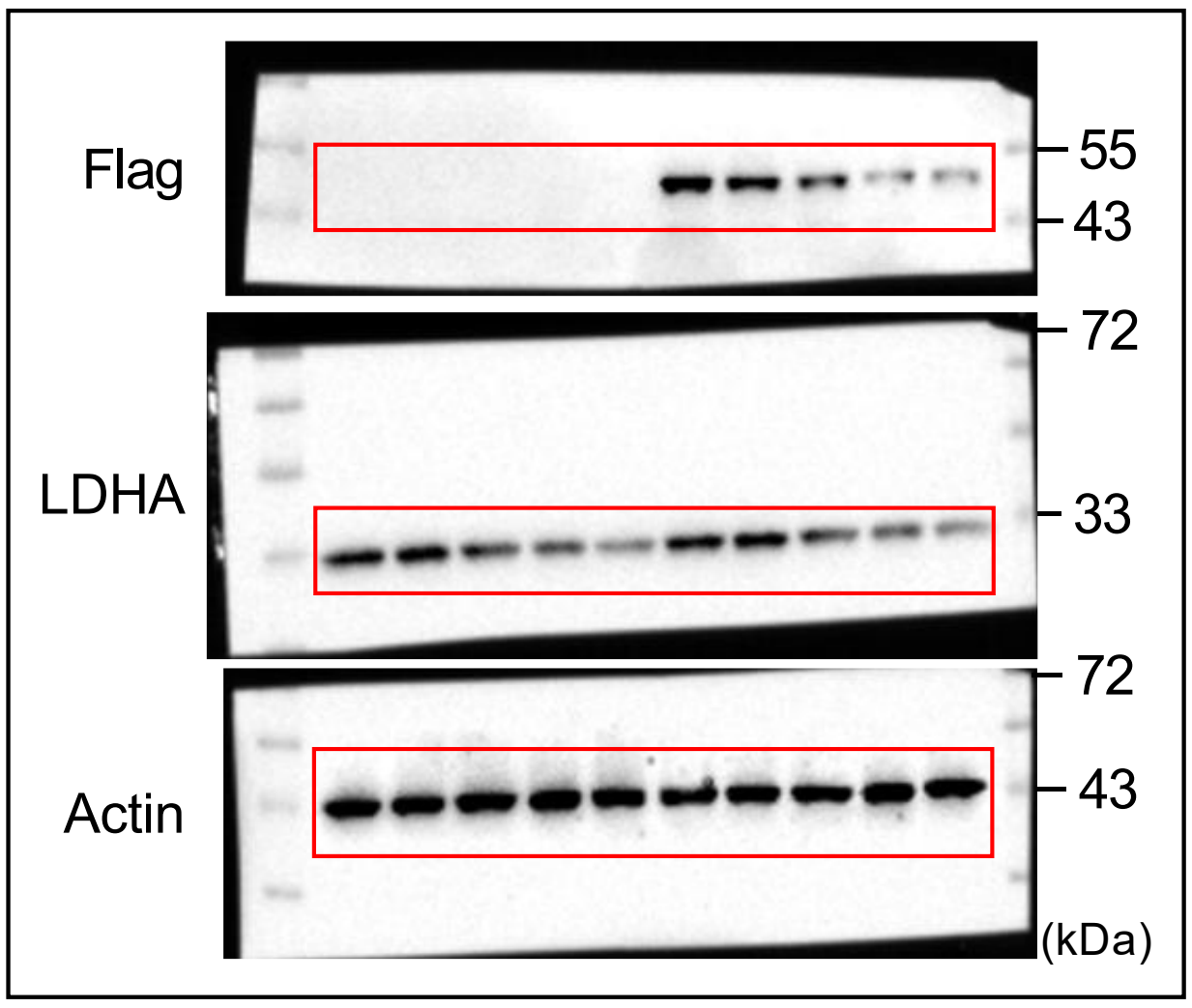

Figure 3c

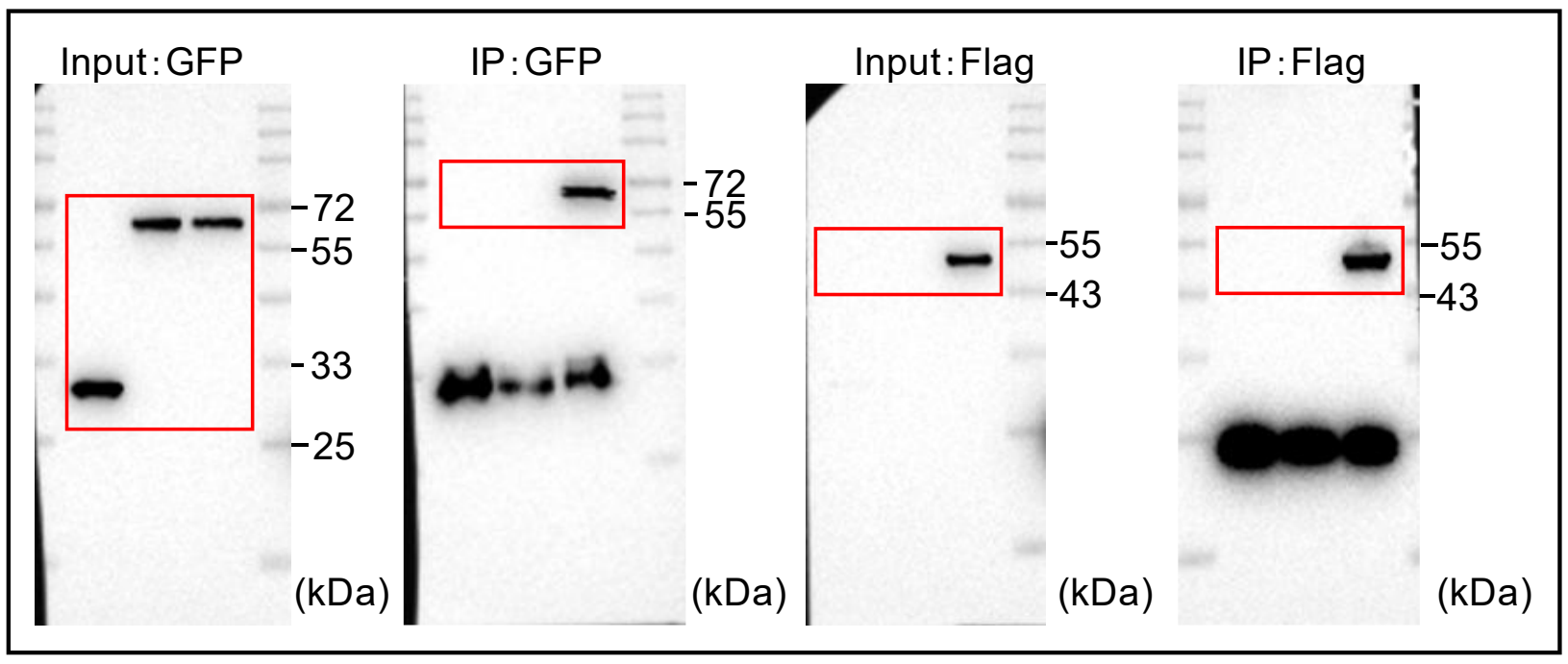

Figure 3d

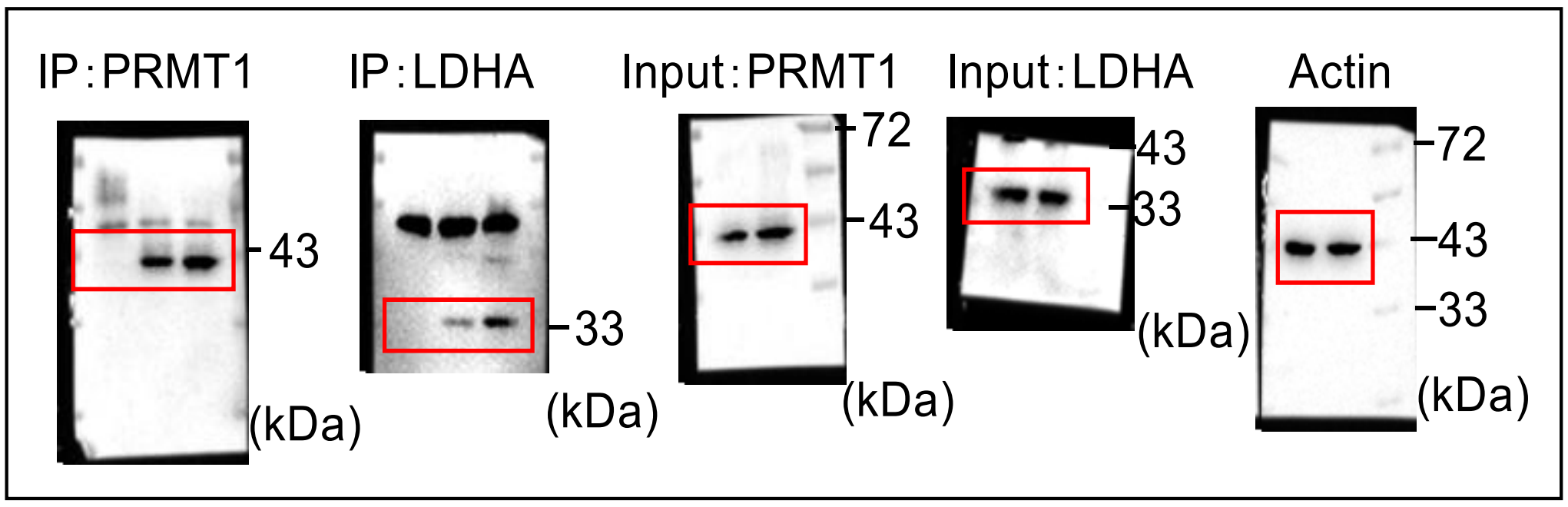

Figure 3e

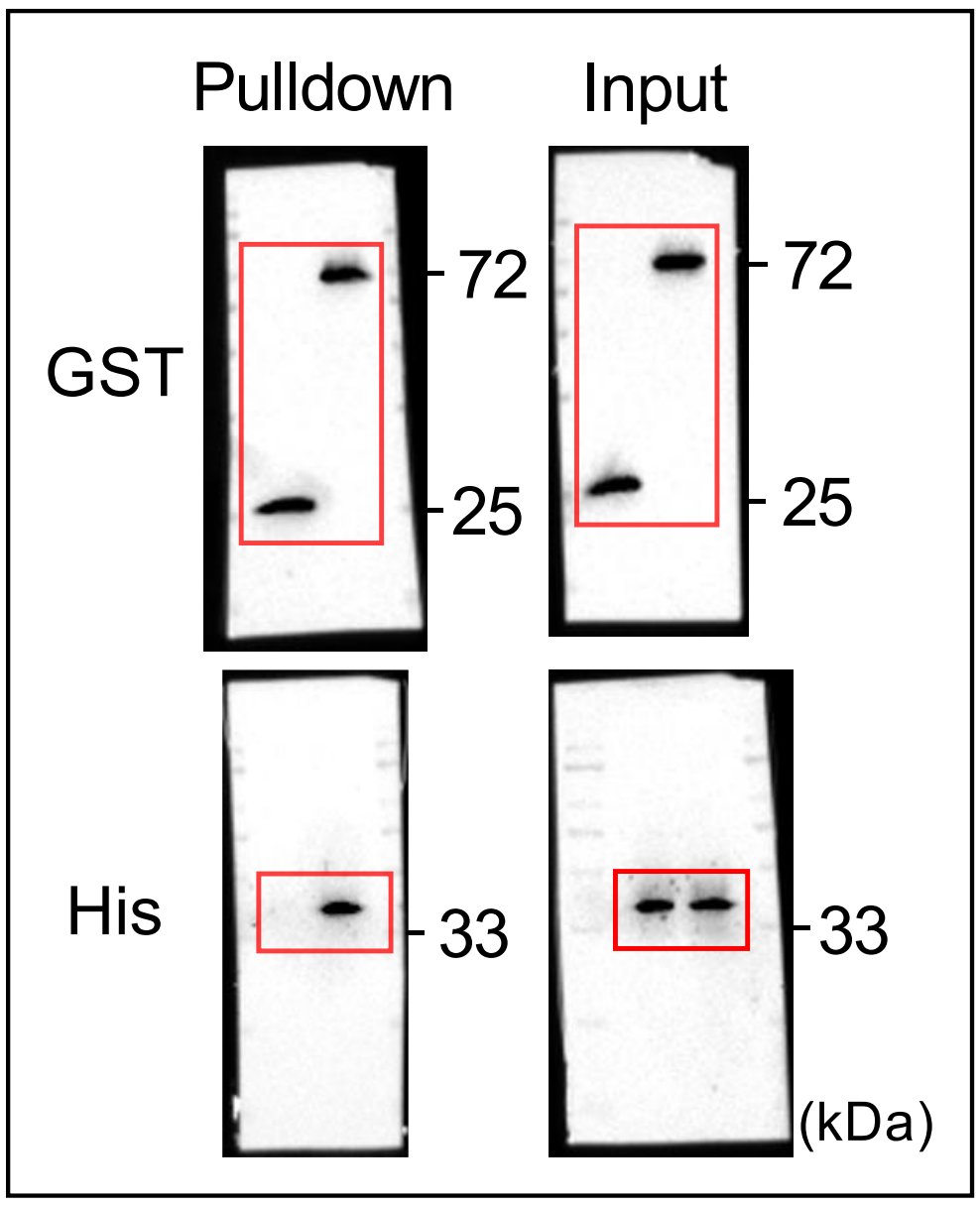

Figure 3k

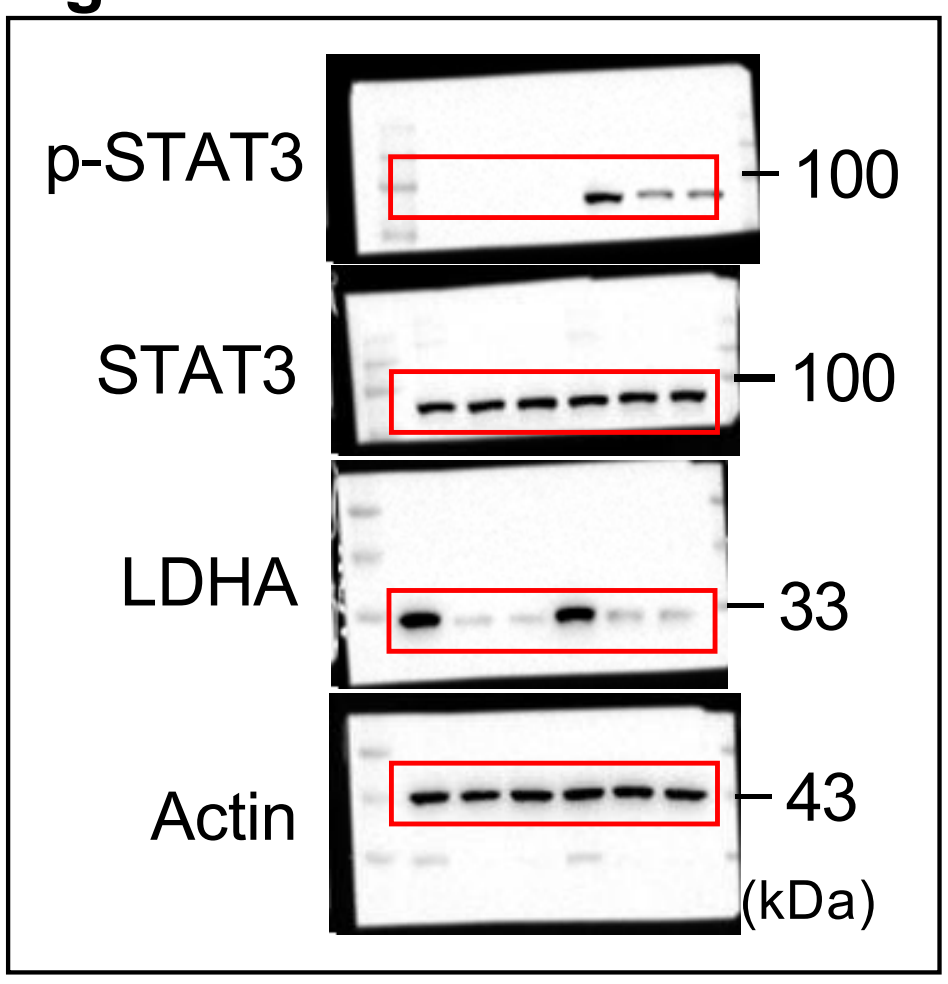

Figure S10 (Related to Figure 3)

Figure 4a

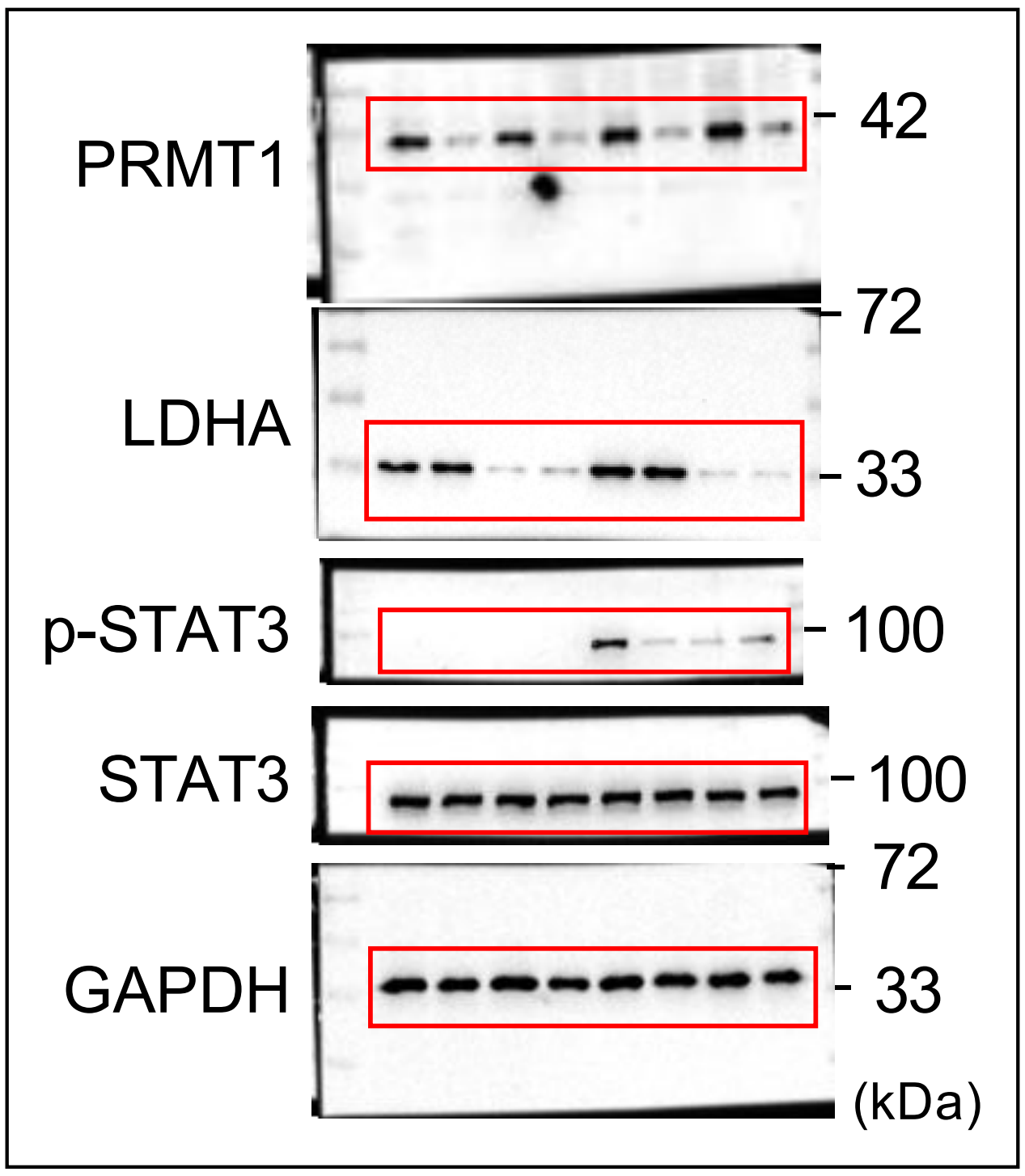

Figure 4b

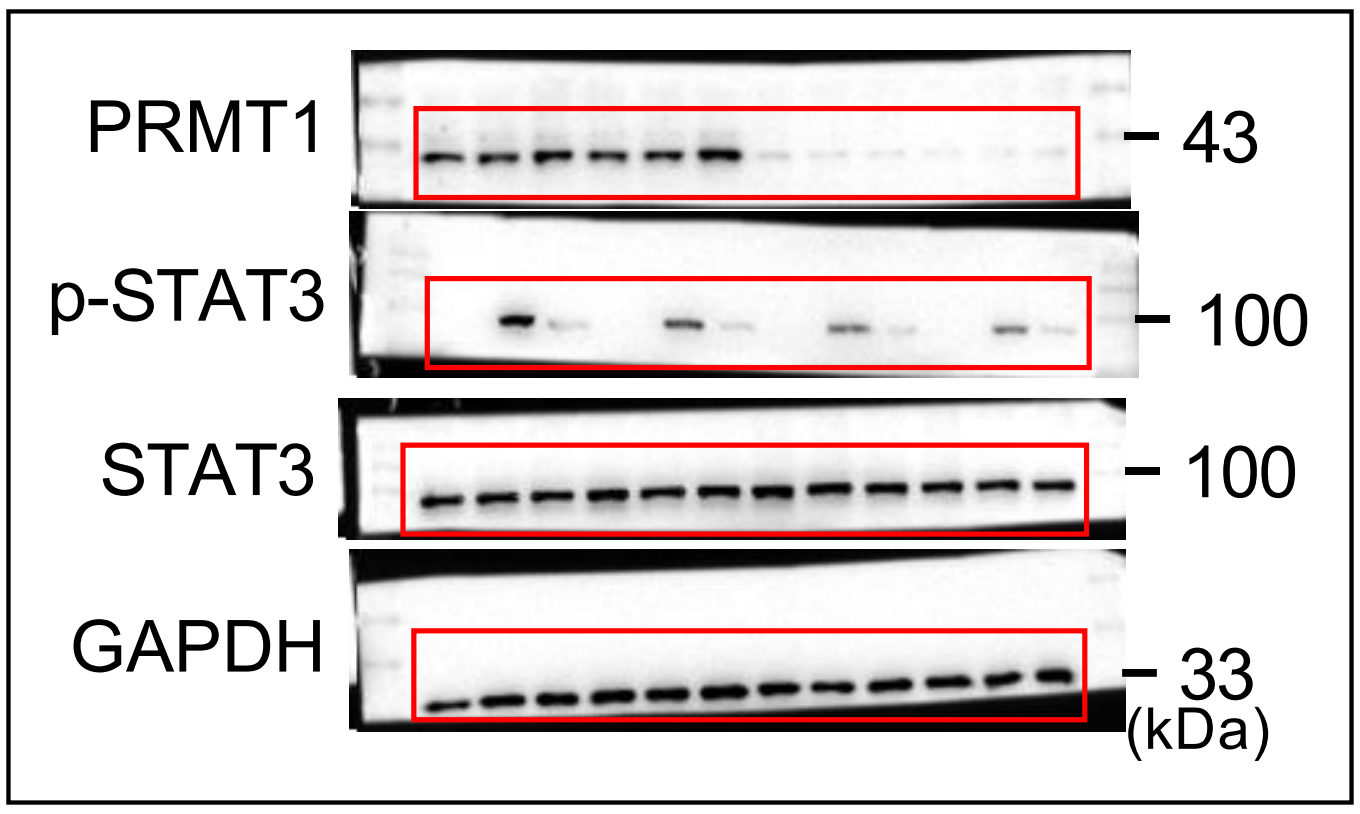

Figure 4f

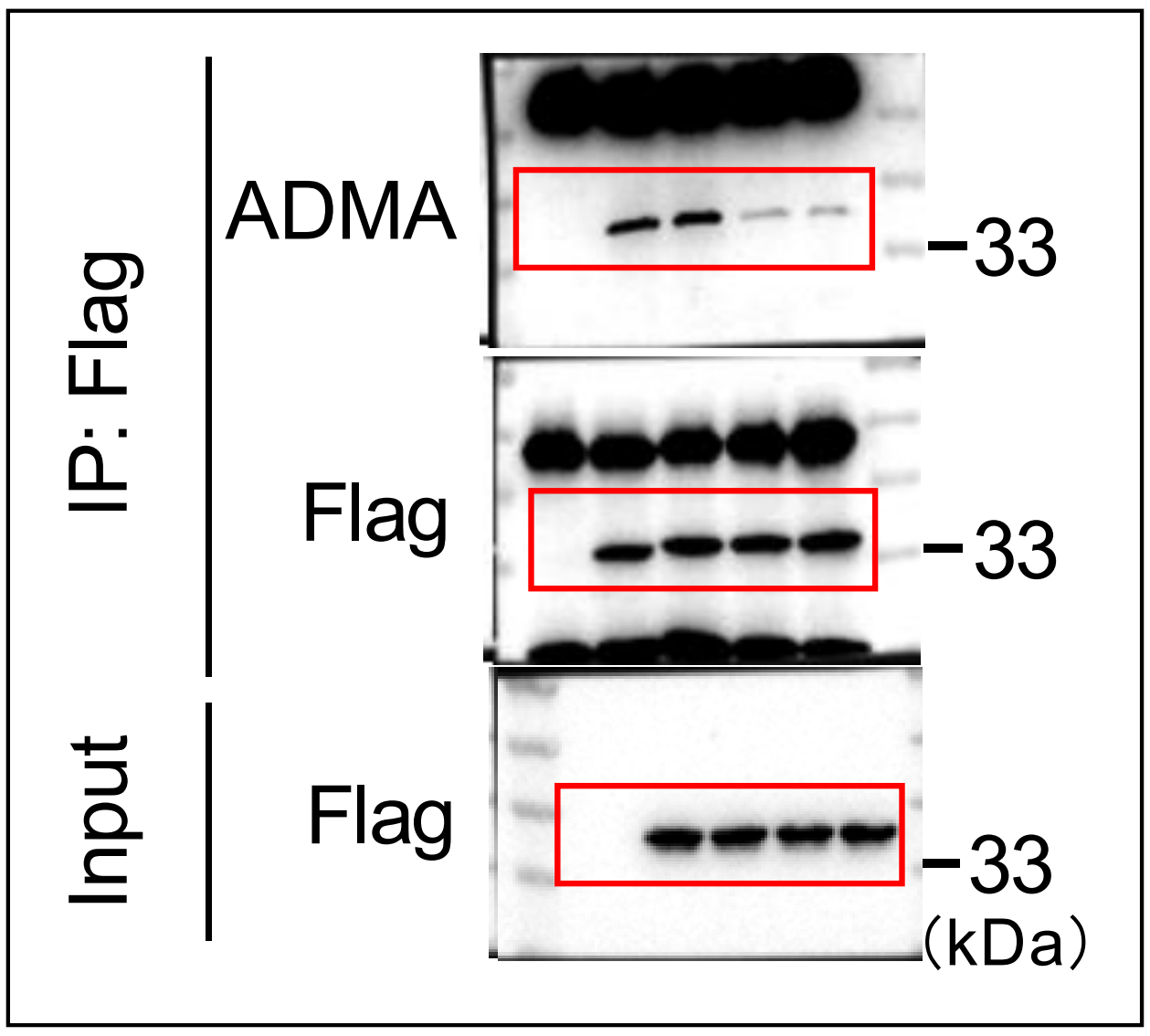

Figure 4g

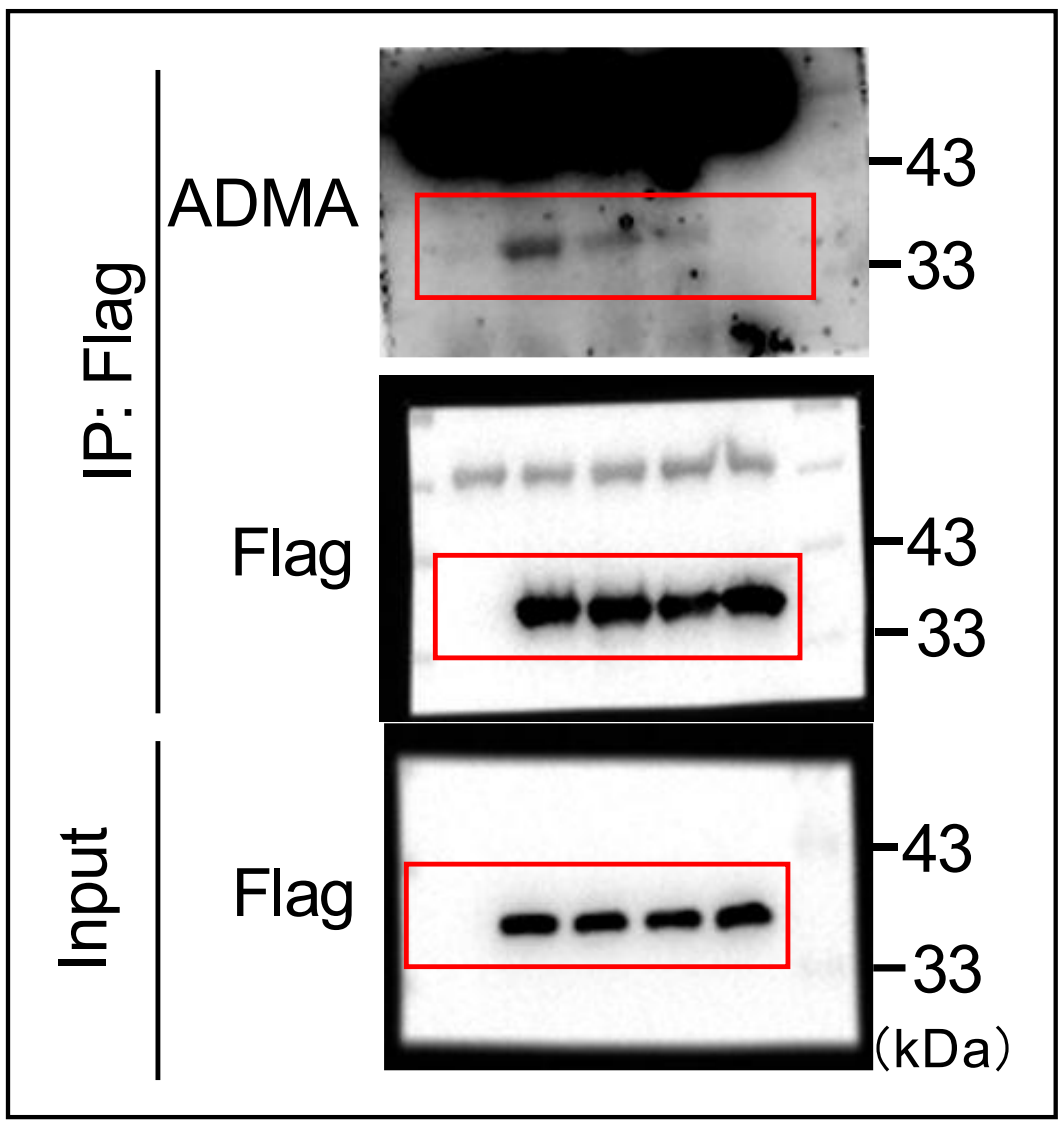

Figure 4l

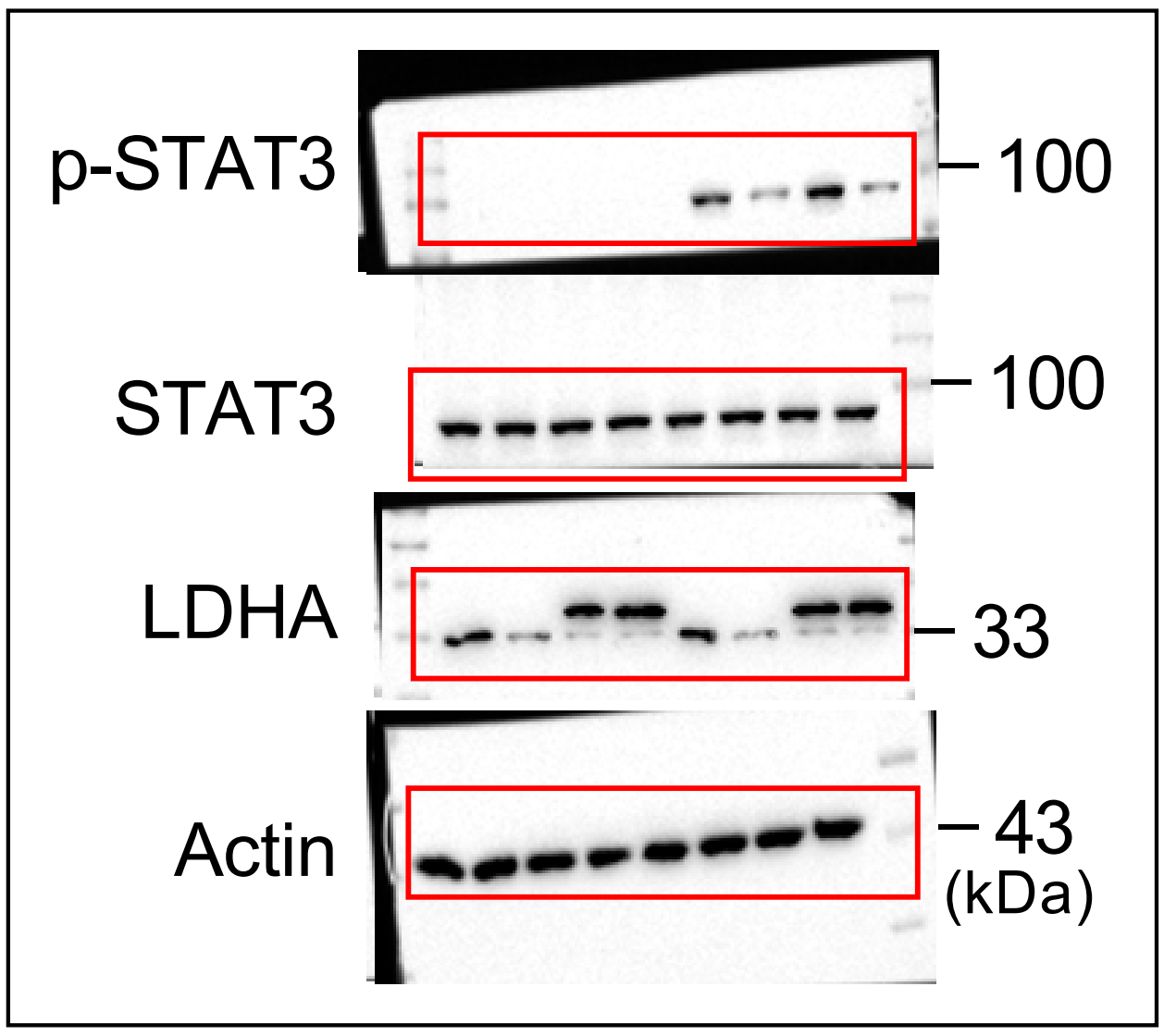

Figure S11 (Related to Figure 4)

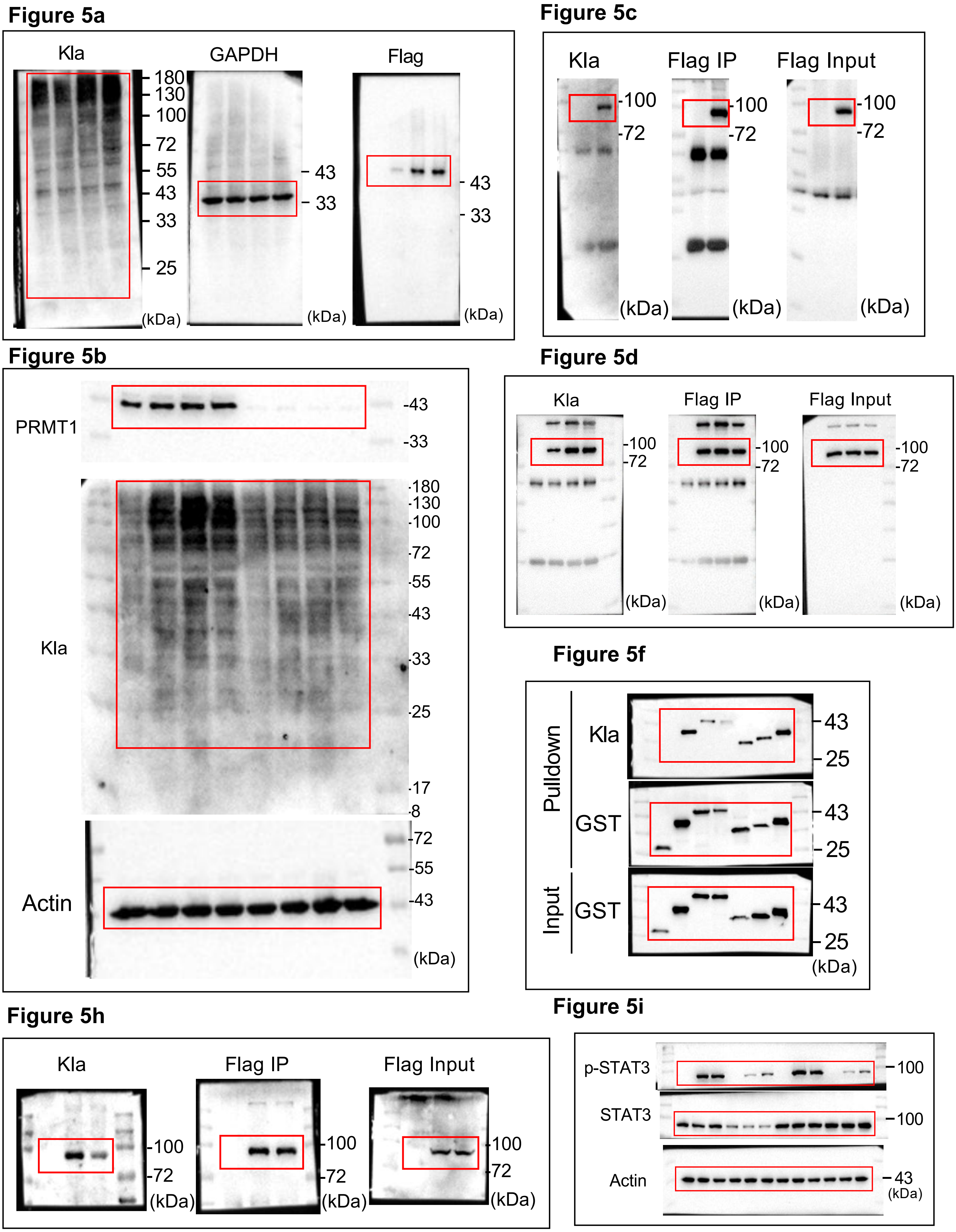

Figure S12 (Related to Figure 5)

Figure 6b

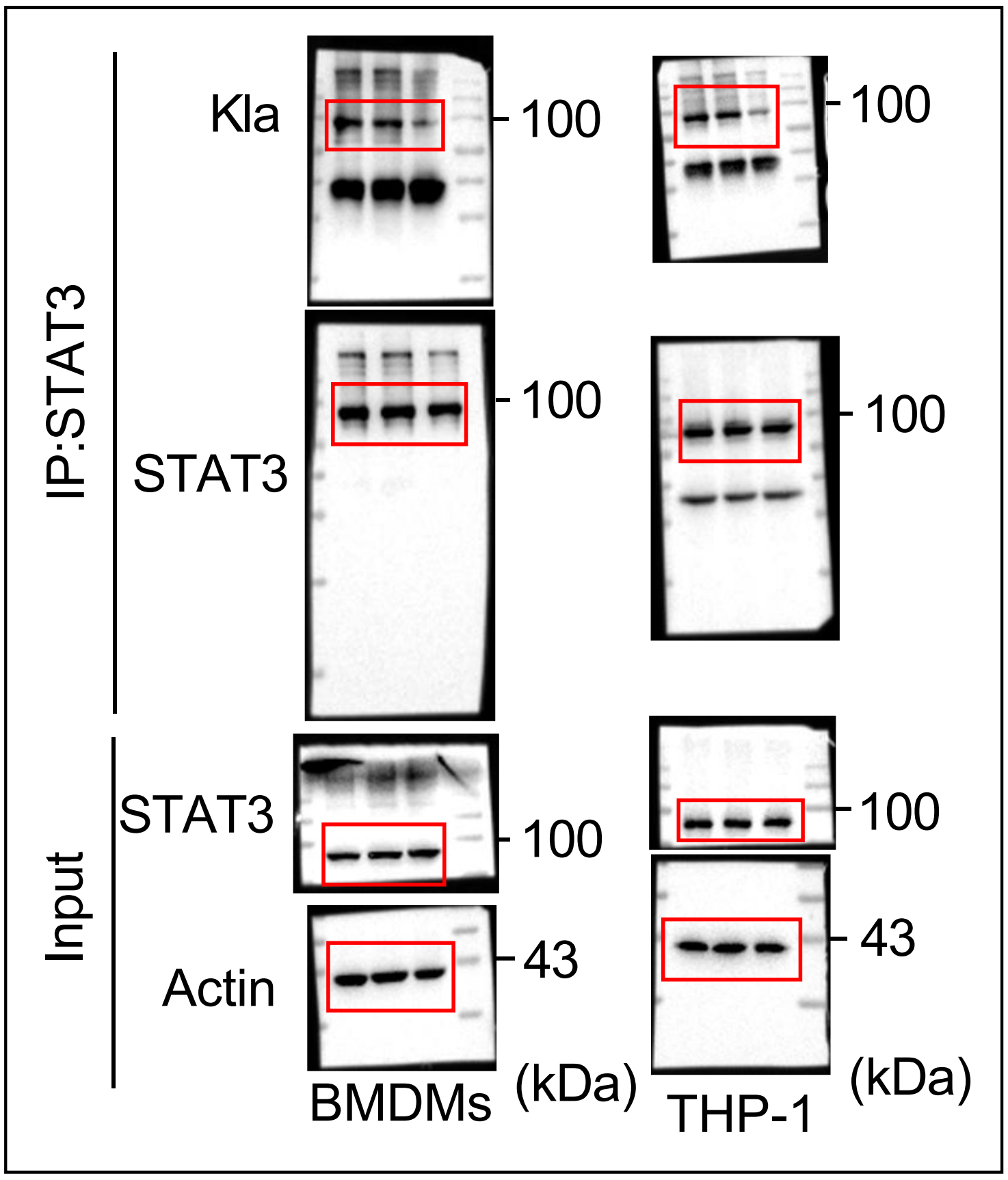

Figure 6c

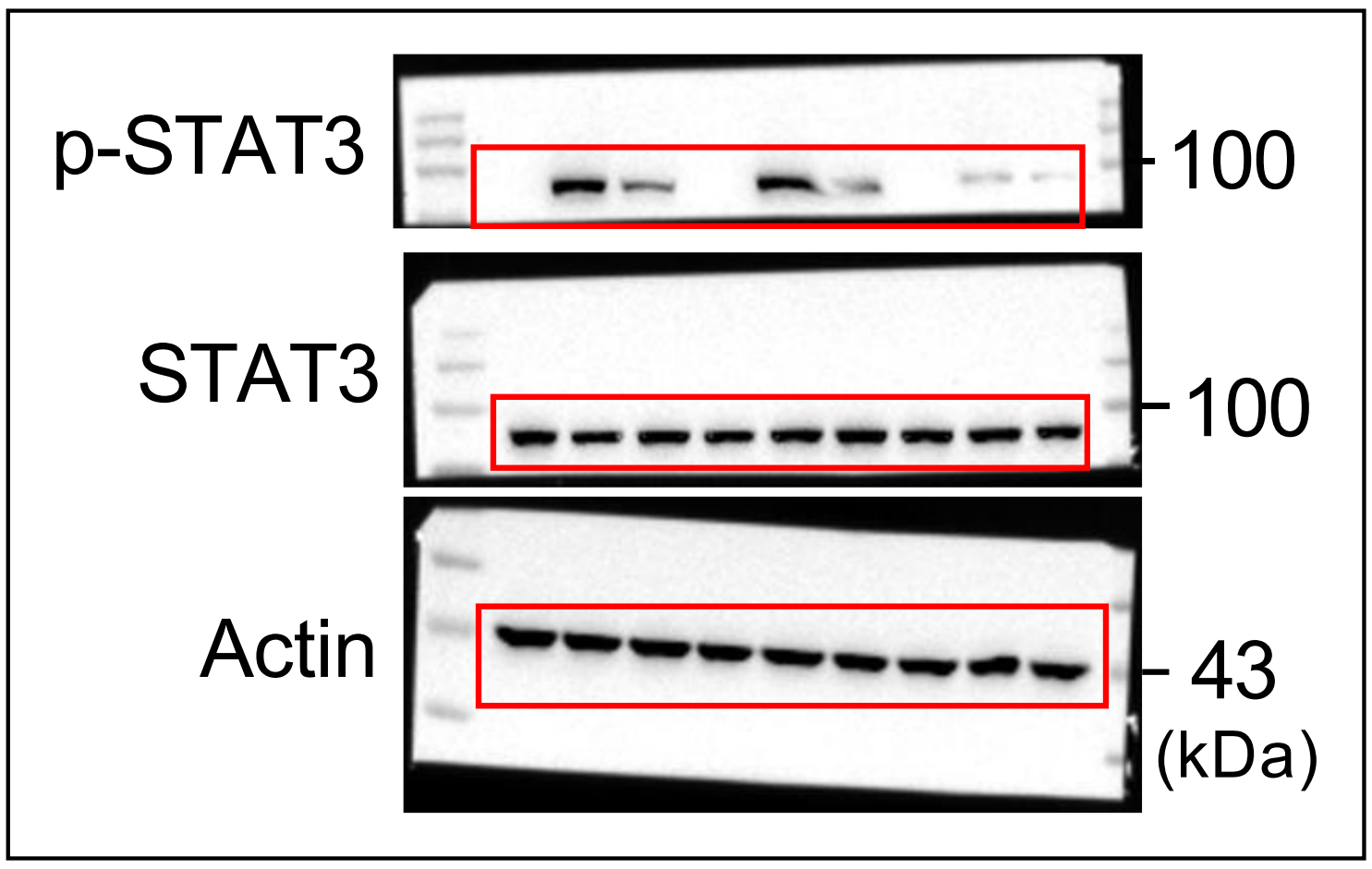

Figure 6m

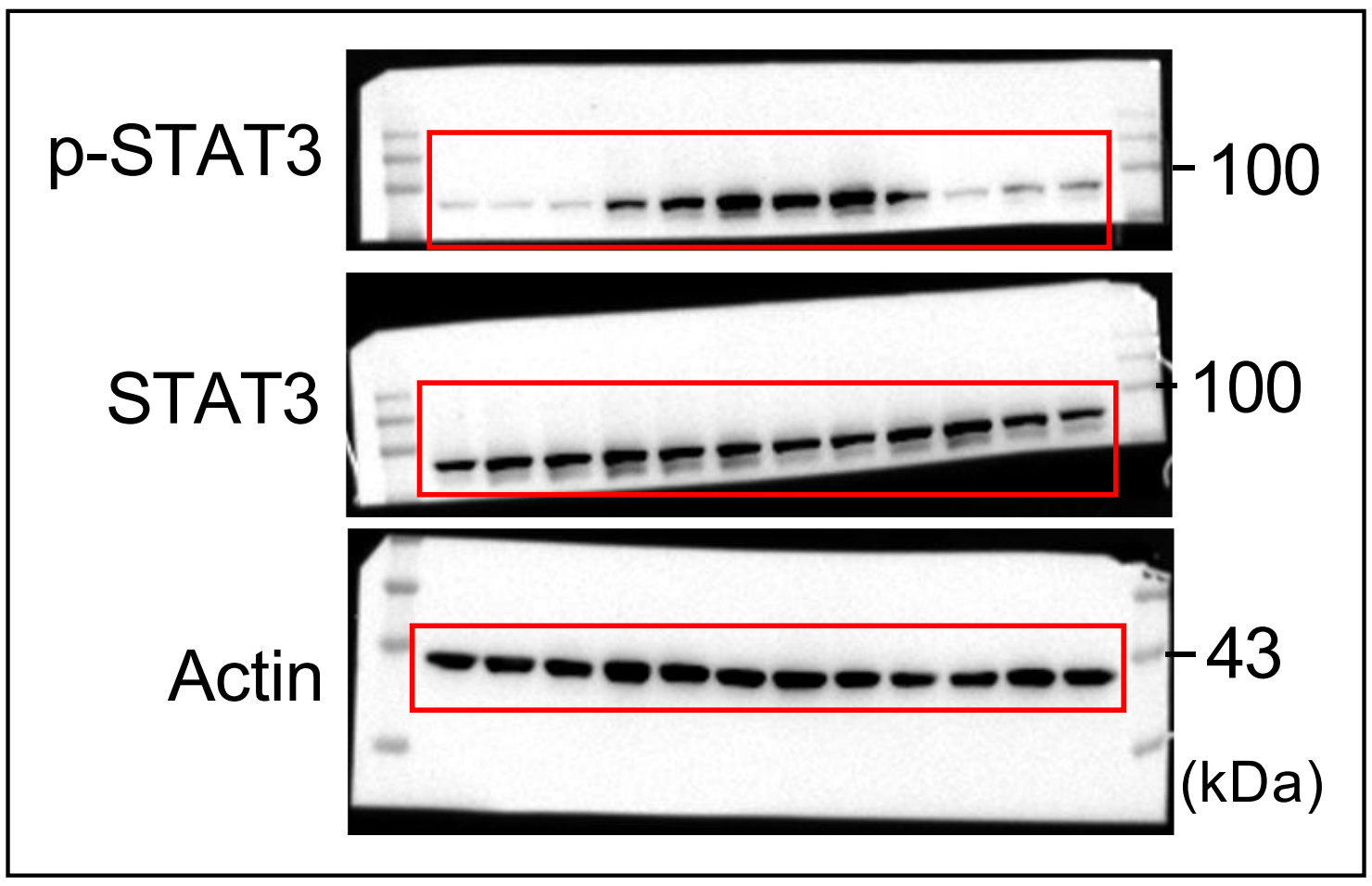

Figure S13 (Related to Figure 6)

Figure S2a

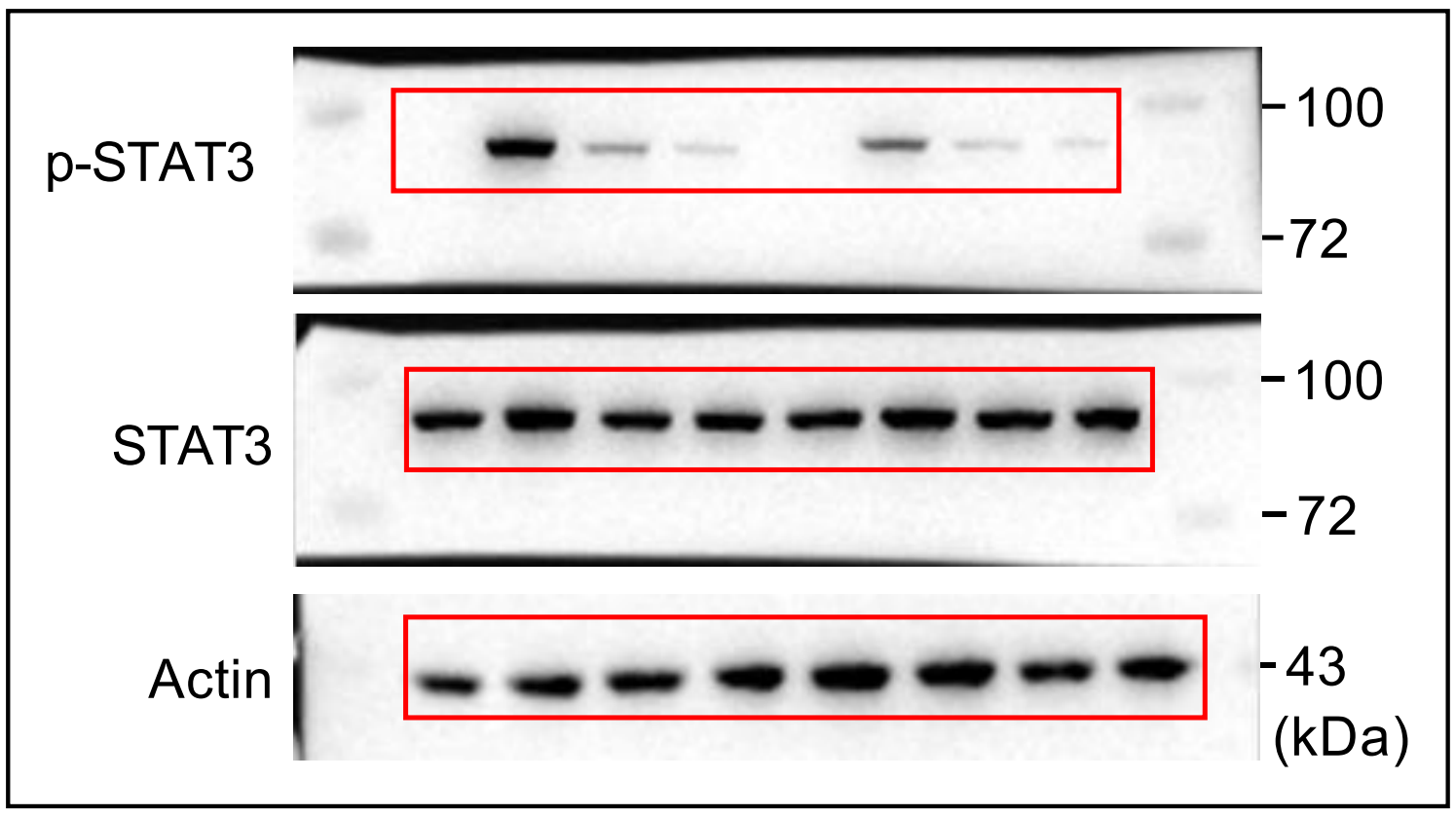

Figure S2f

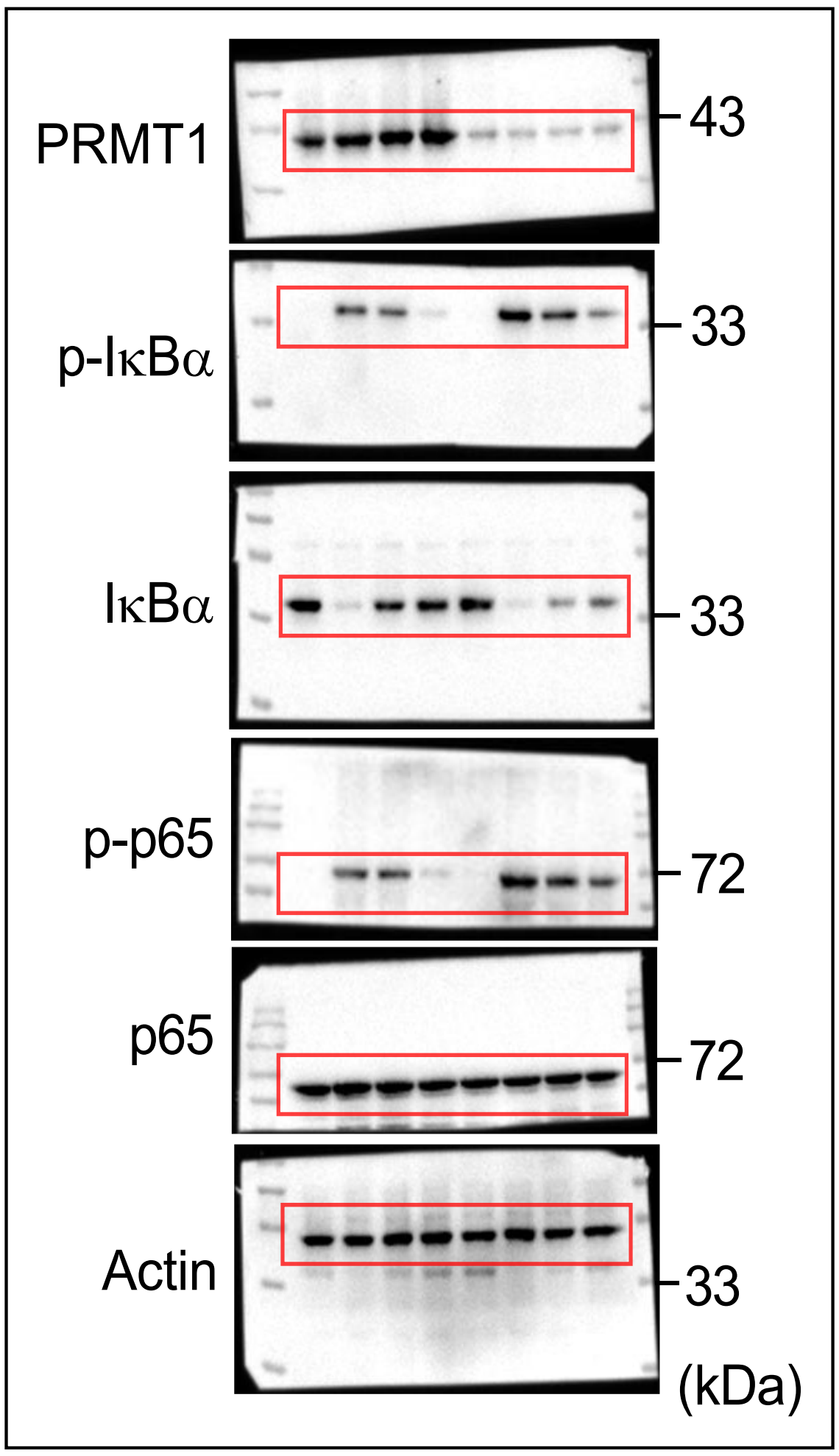

Figure S2g

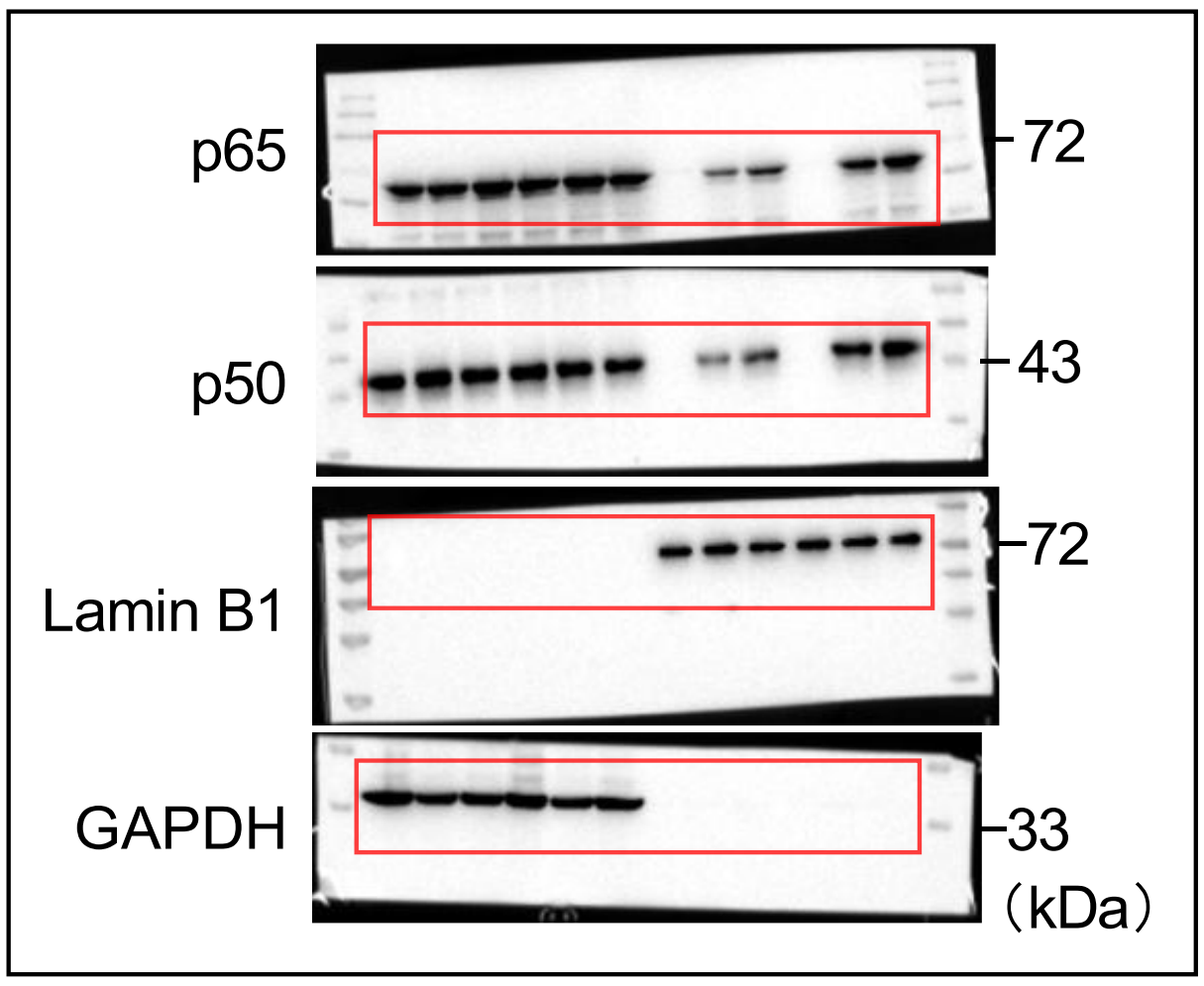

Figure S2h

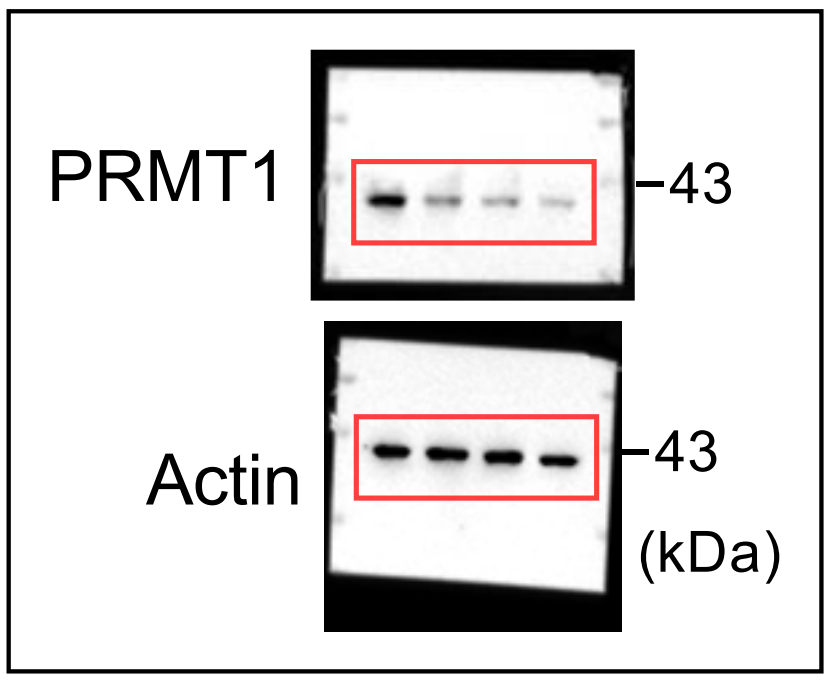

Figure S2i

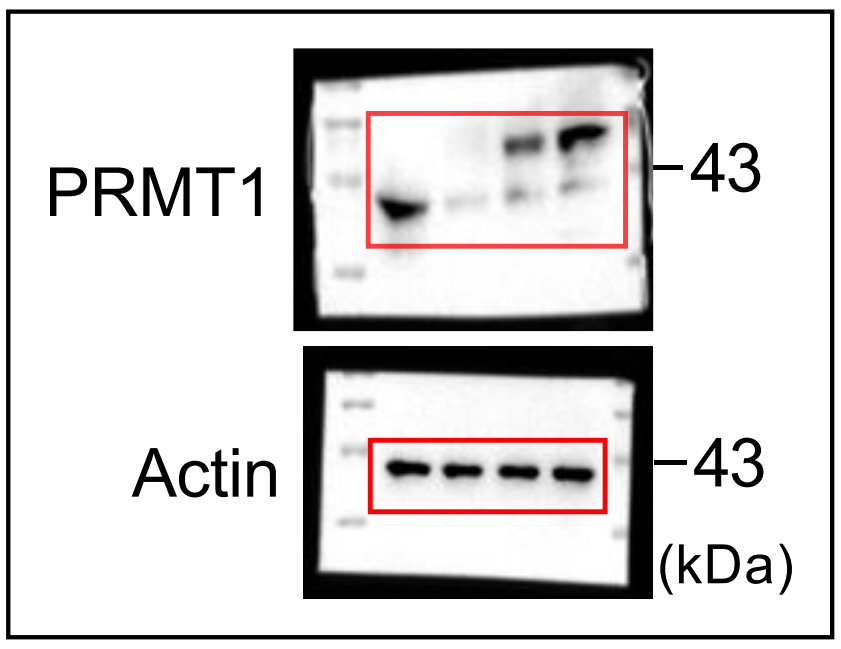

Figure S14 (Related to Figure S2)

Figure S3b

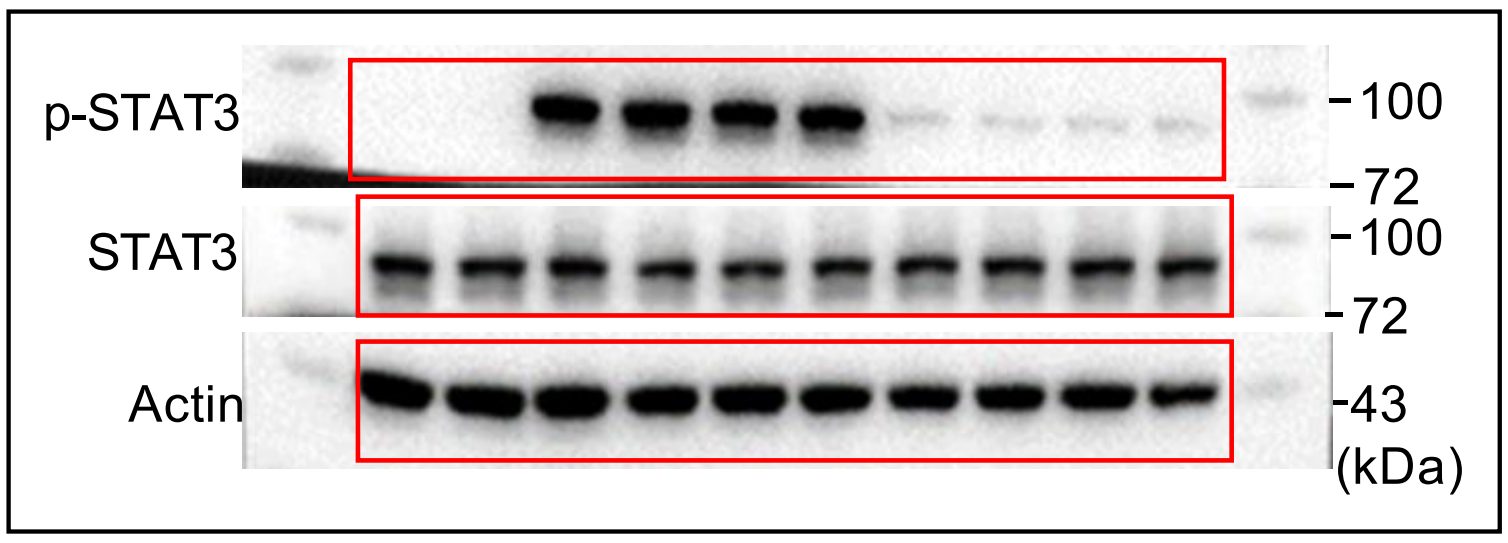

Figure S3c

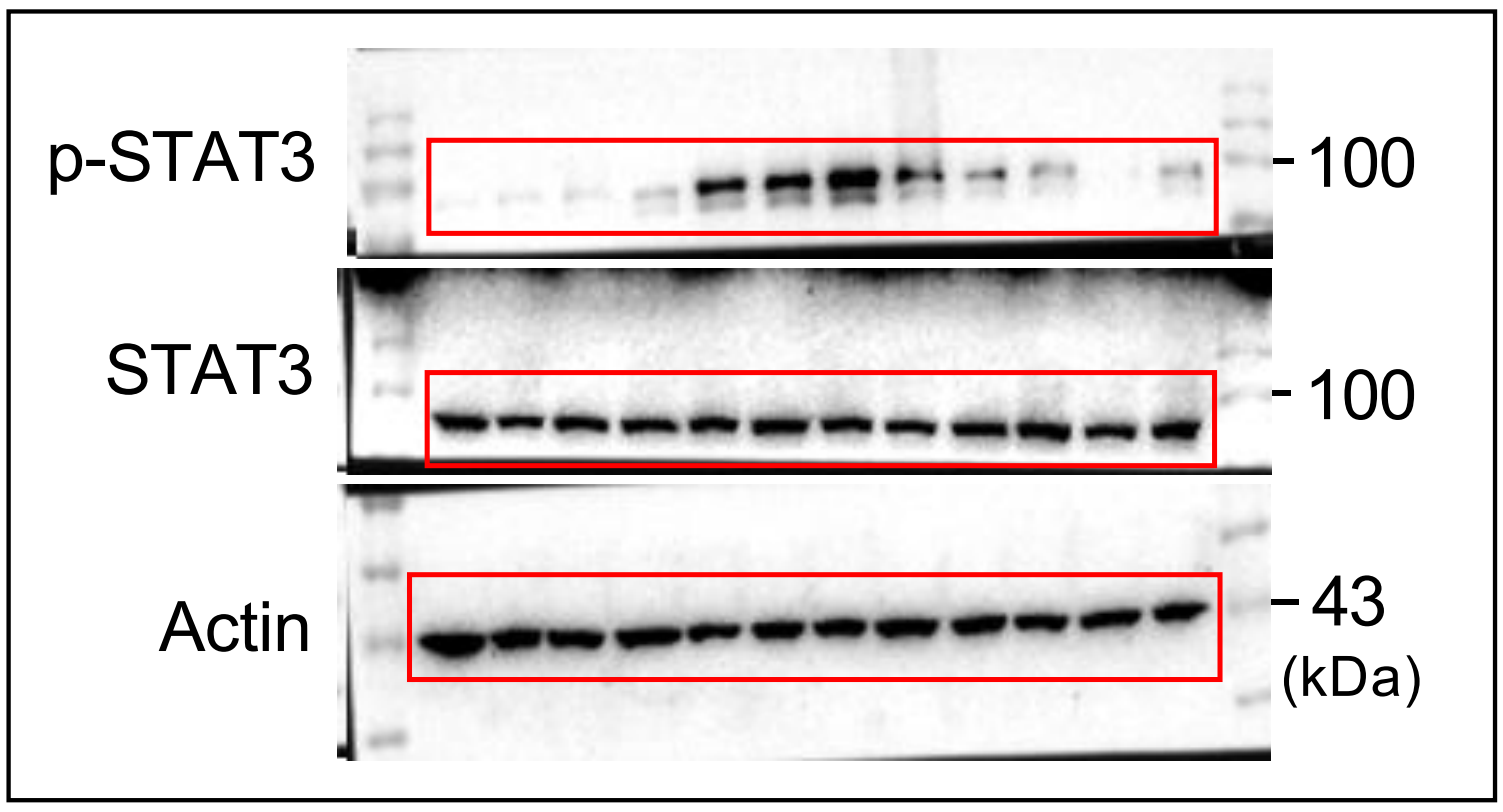

Figure S15 (Related to Figure S3)

Figure S4a

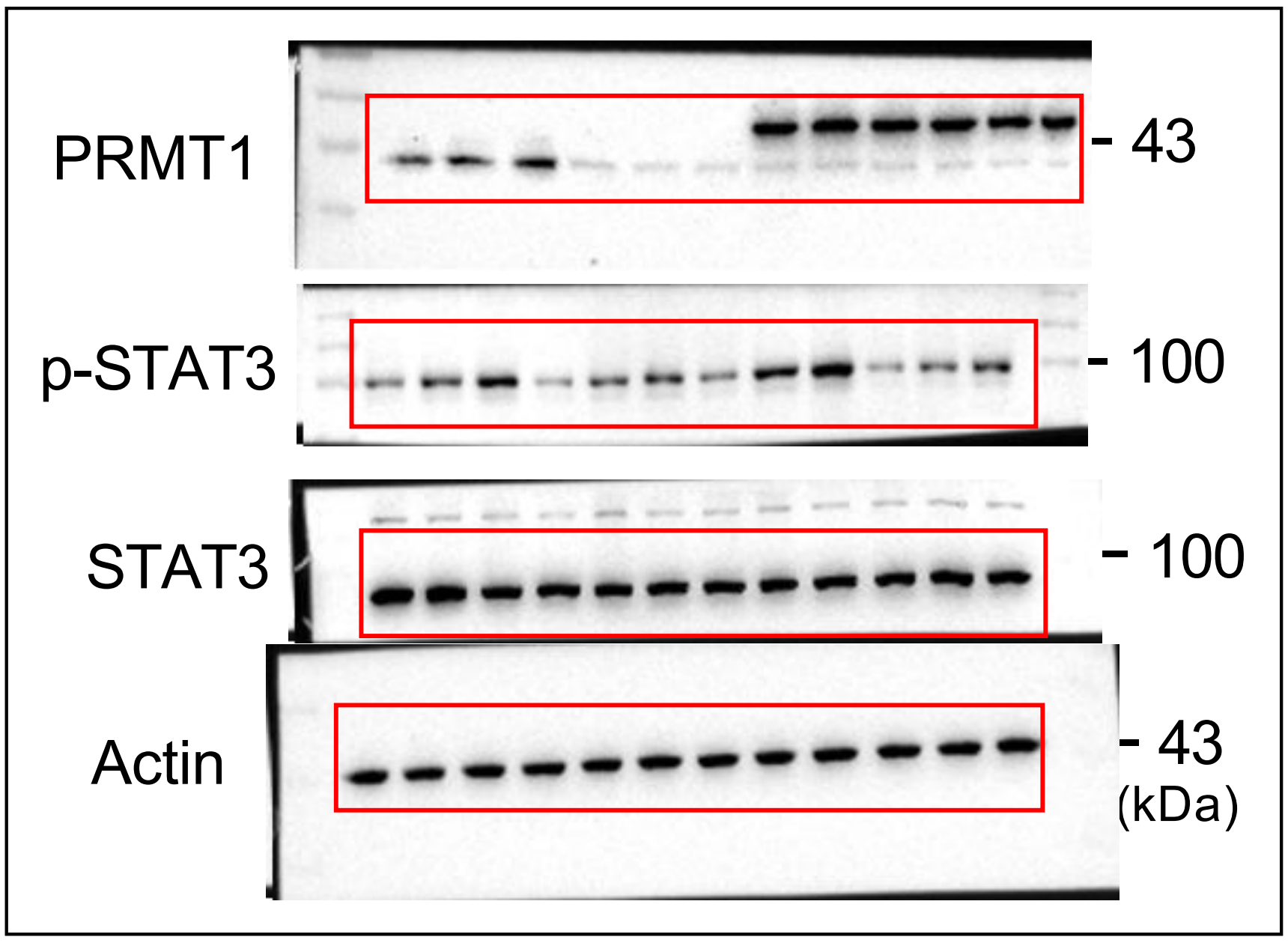

Figure S4c

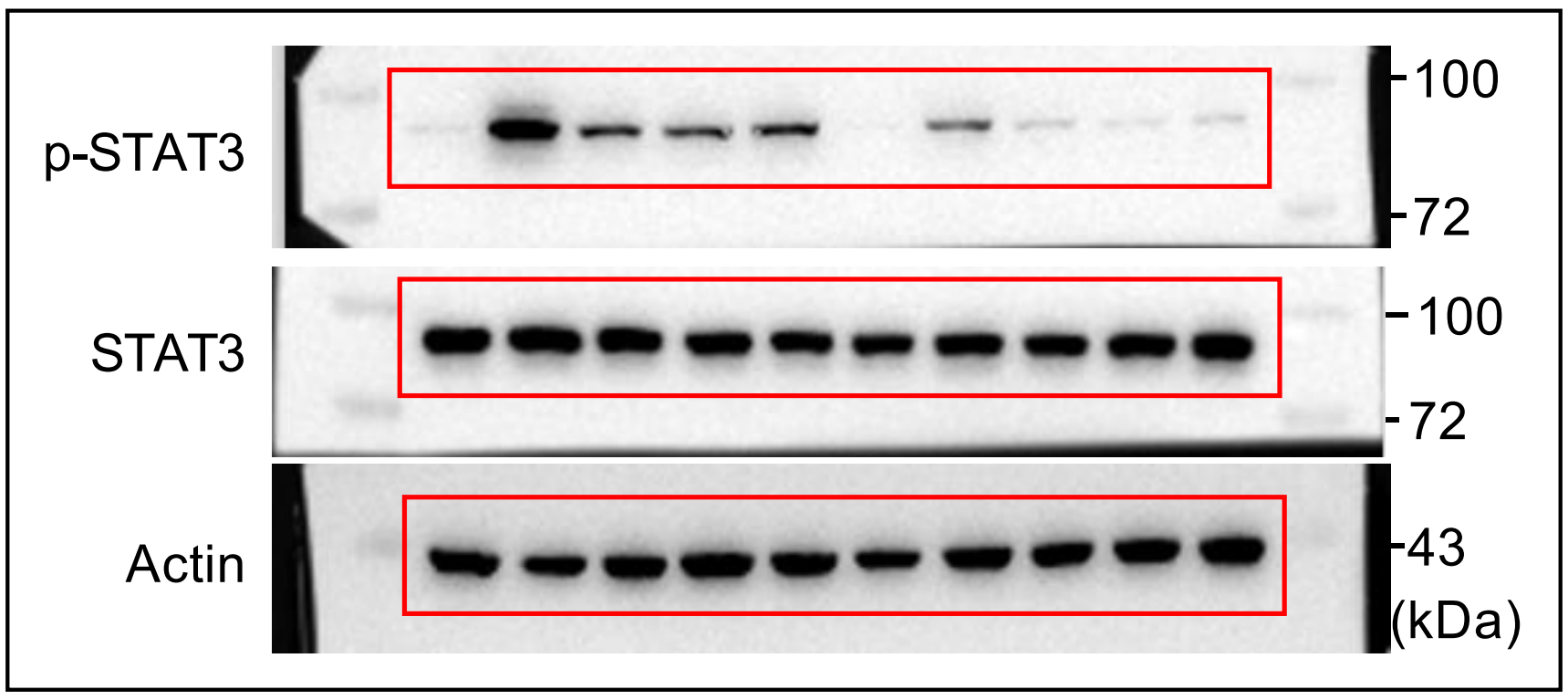

Figure S16 (Related to Figure S4)

Figure S5b

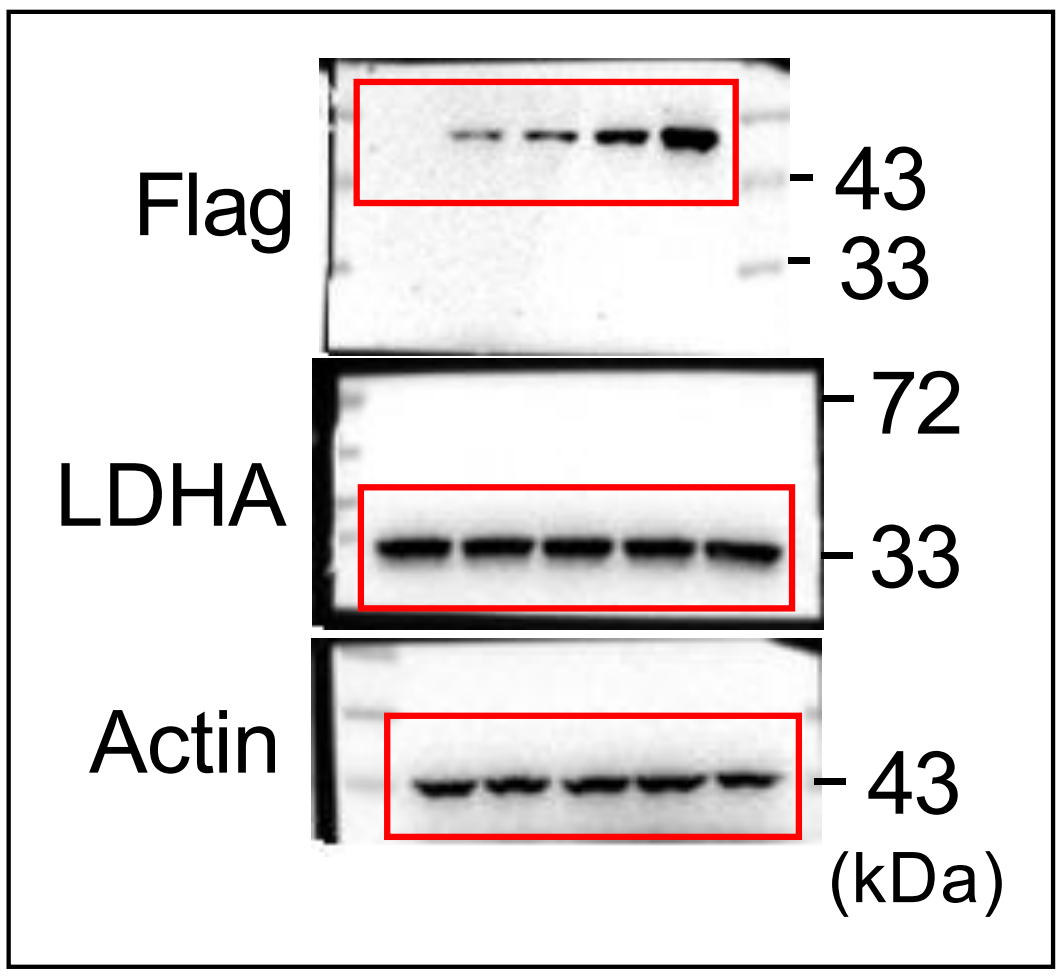

Figure S5c

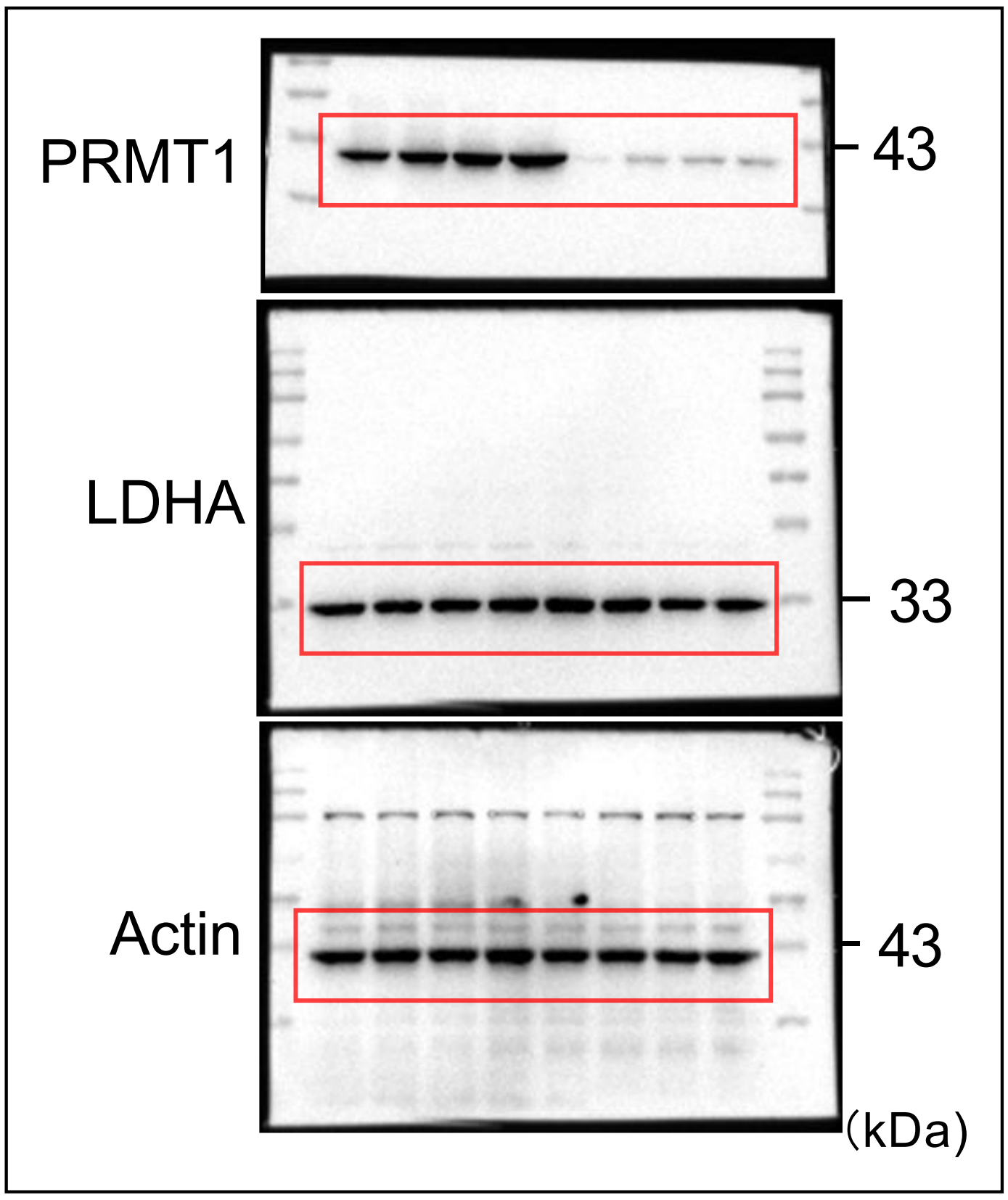

Figure S17 (Related to Figure S5)

Figure S6c

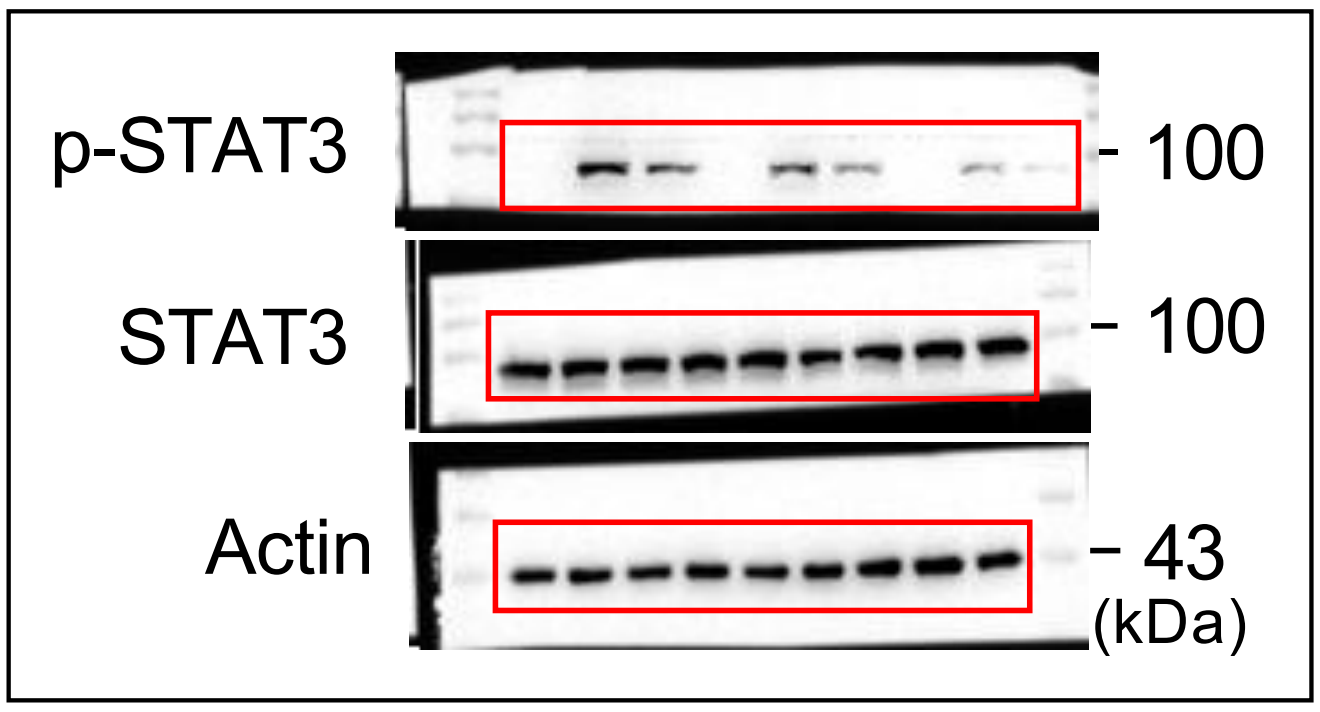

Figure S6d

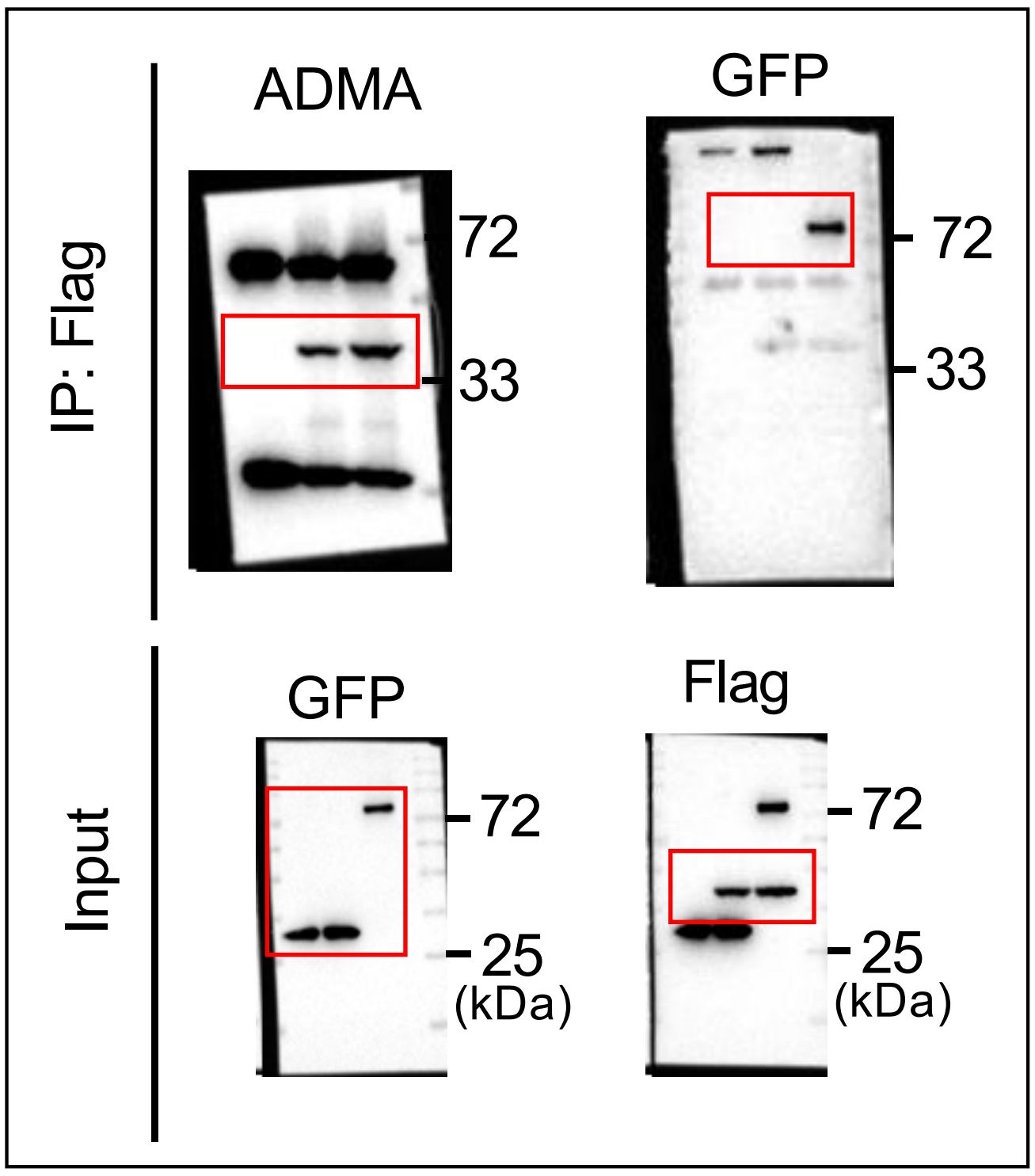

Figure S6e

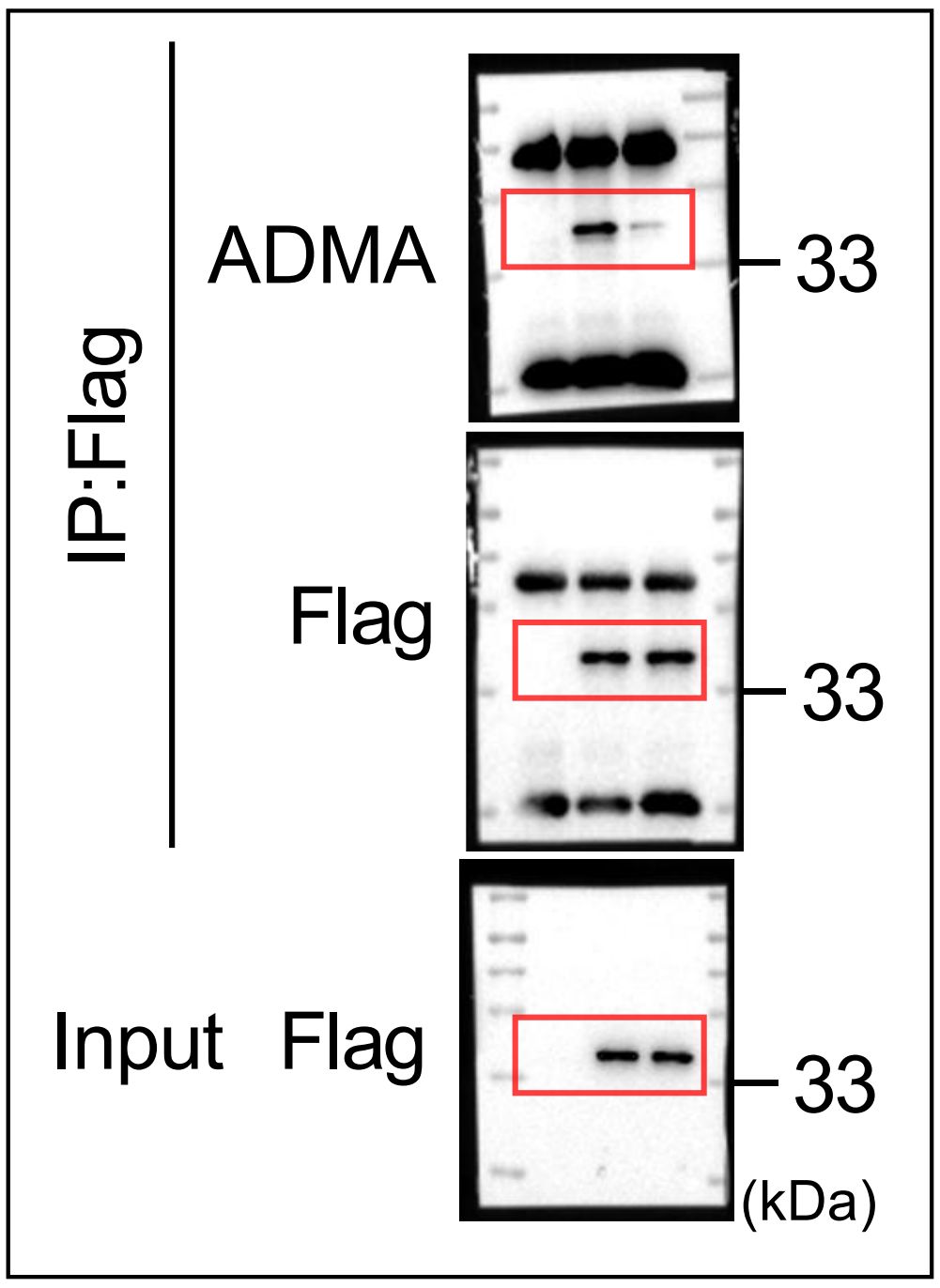

Figure S18 (Related to Figure S6)

Figure S7b

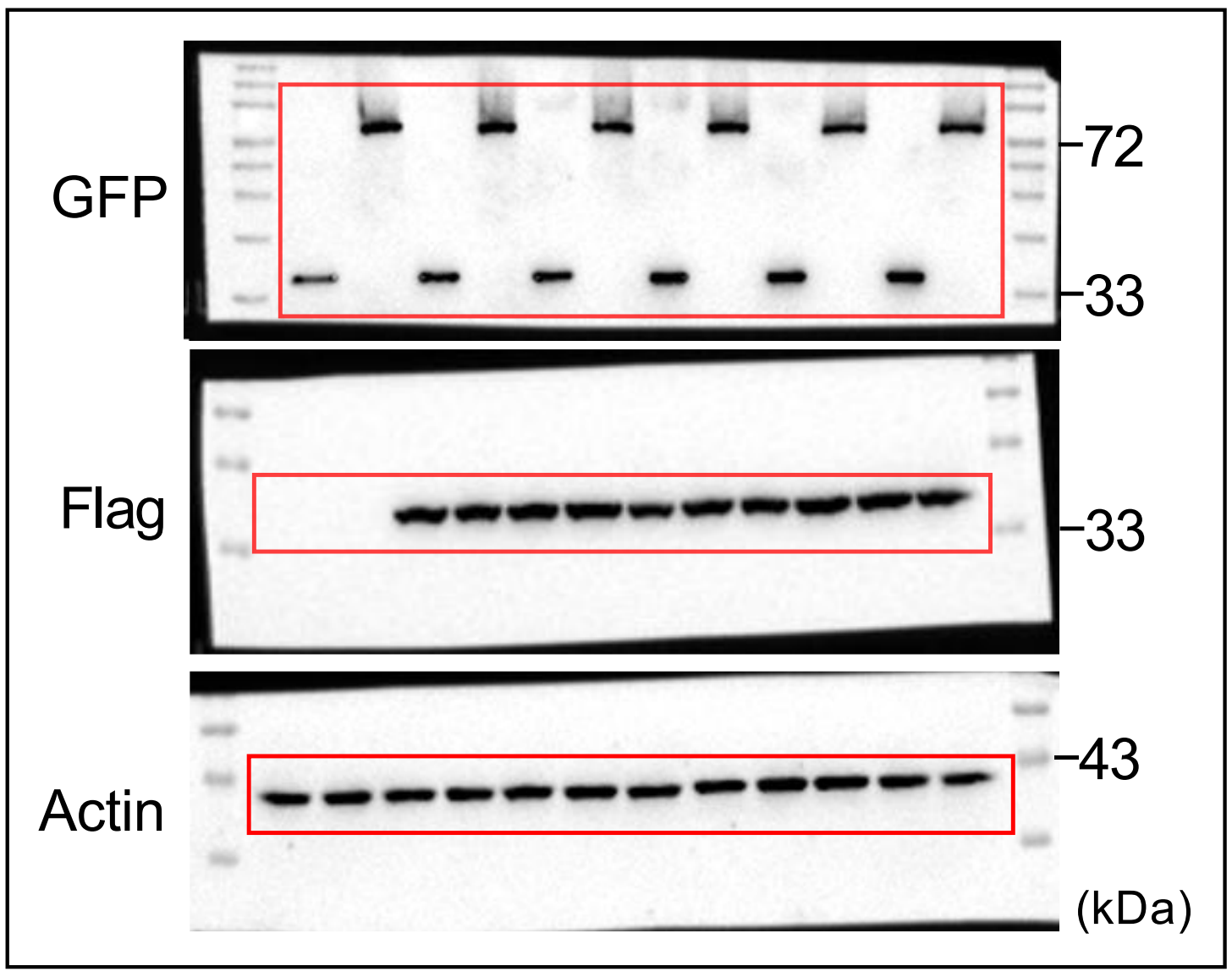

Figure S7f

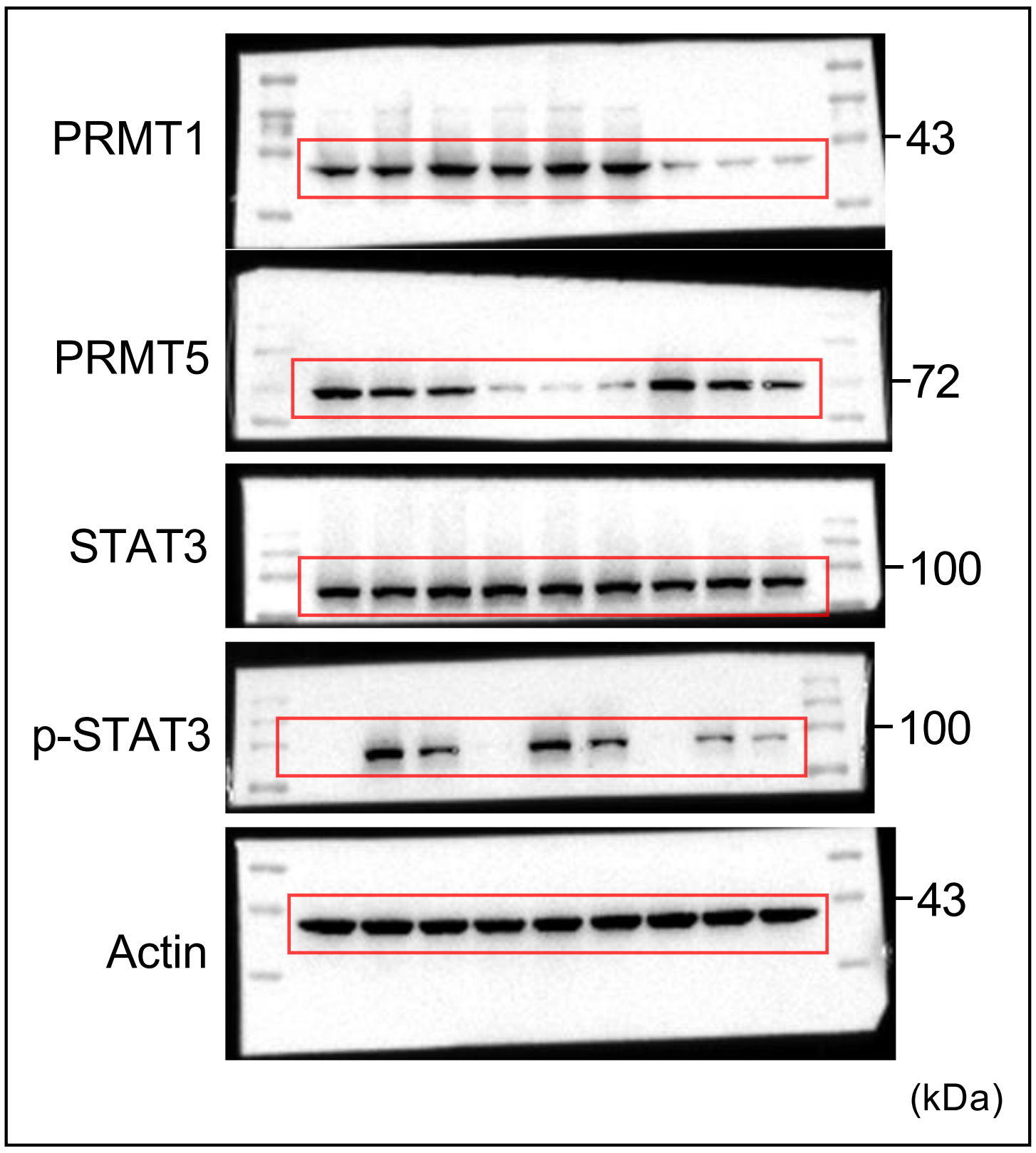

Figure S7g

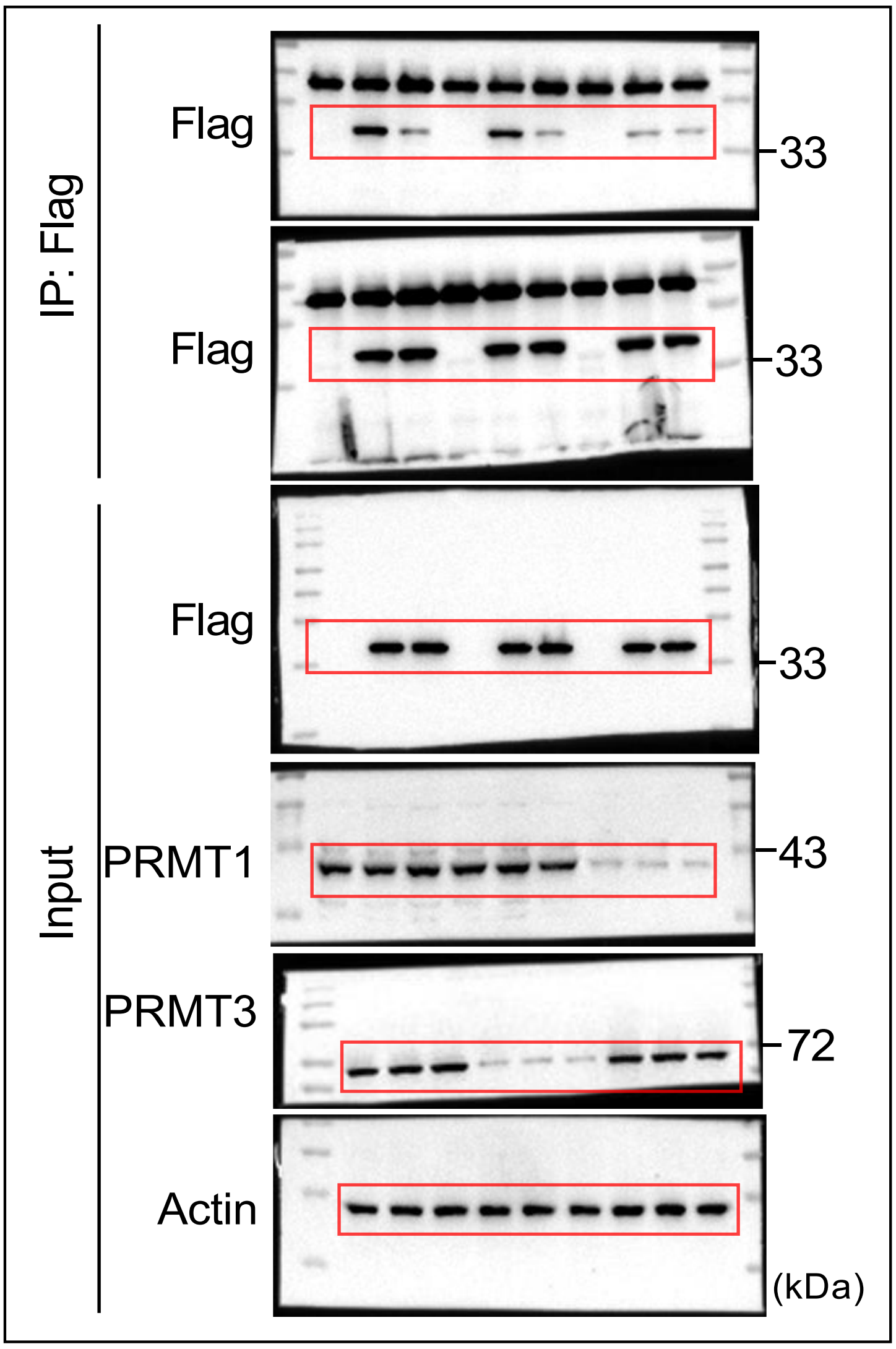

Figure S7h

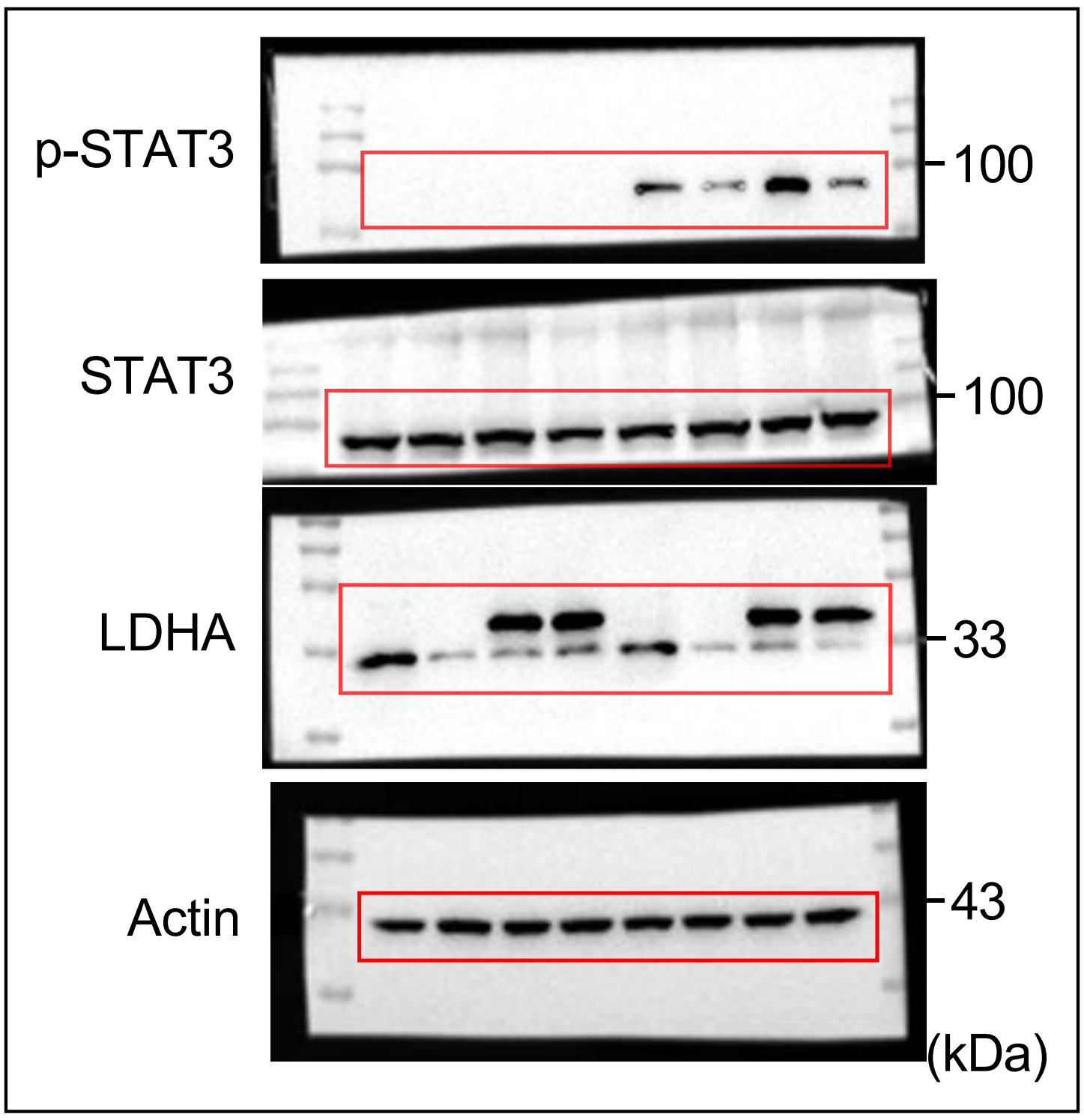

Figure S19 (Related to Figure S7)

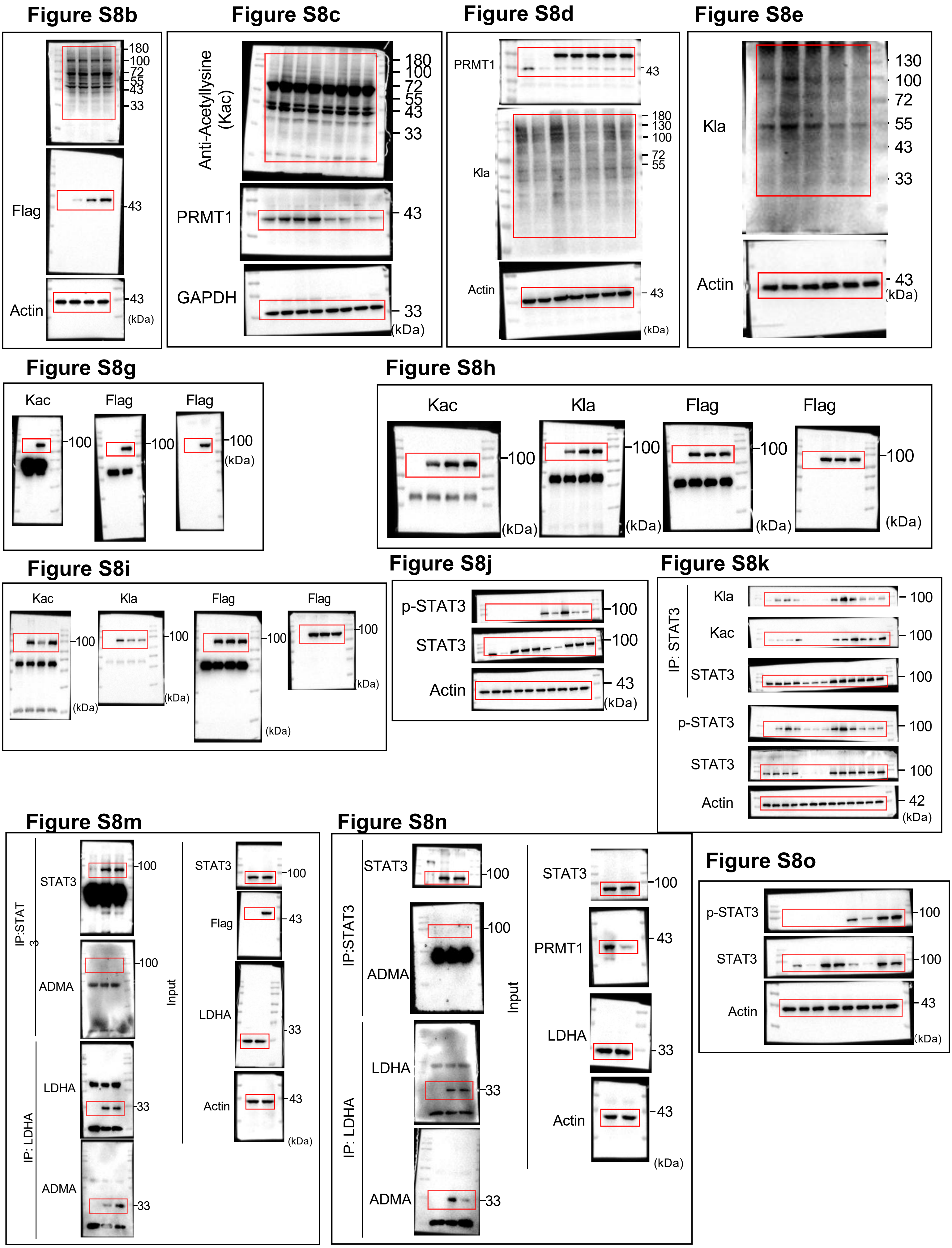

Figure S20 (Related to Figure S8)
